# AI-driven reclassification of multiple sclerosis progression

**DOI:** 10.1038/s41591-025-03901-6

**Published:** 2025-08-20

**Authors:** Habib Ganjgahi, Dieter A. Häring, Piet Aarden, Gordon Graham, Yang Sun, Stephen Gardiner, Wendy Su, Claude Berge, Antje Bischof, Elizabeth Fisher, Laura Gaetano, Stefan P. Thoma, Bernd C. Kieseier, Thomas E. Nichols, Alan J. Thompson, Xavier Montalban, Fred D. Lublin, Ludwig Kappos, Douglas L. Arnold, Robert A. Bermel, Heinz Wiendl, Chris C. Holmes

**Affiliations:** 1 Department of Statistics, University of Oxford, Oxford, UK; 2 Big Data Institute, Li Ka Shing Centre for Health Information and Discovery, Nuffield Department of Medicine, University of Oxford, Oxford, UK; 3 Novartis Pharma AG, Basel, Switzerland; 4 Roche, Basel, Switzerland; 5 Clinic and Polyclinic for Neurology, University Hospital Münster, Münster, Germany; 6 Novartis Biomedical Research, Cambridge, MA USA; 7 Department of Neurology, Medical Faculty, Heinrich Heine University, Duesseldorf, Germany; 8 National Hospital for Neurology and Neurosurgery (NHNN), London, UK; 9 Department of Neurology and Centre dʼEsclerosi Multiple de Catalunya (Cemcat), Vall d’Hebron University Hospital, Barcelona, Spain; 10 Corinne Goldsmith Dickinson Center for Multiple Sclerosis, Icahn School of Medicine at Mount Sinai, New York, NY USA; 11 Clinic and Policlinic for Neurology, and MS Center, Department of Head, Spine and Neuromedicine, University Hospital Basel, Basel, Switzerland; 12 Research Center for Clinical Neuroimmunology and Neuroscience (RC2NB), Departments of Biomedicine and Clinical Research, University Hospital and University of Basel, Basel, Switzerland; 13 Brain Imaging Centre, Montreal Neurological Institute and Hospital, McGill University, Montréal, QC Canada; 14 Department of Neurology, Mellen MS Center, Cleveland Clinic, Cleveland, OH USA; 15 Department of Neurology with Institute of Translational Neurology, University Hospital Münster, Münster, Germany; 16 Brain & Mind Institute, University of Sydney, Sydney, New South Wales Australia; 17 Ellison Institute of Technology, Oxford, UK; 18 Present Address: Department of Neurology and Neurophysiology, University of Freiburg, Freiburg, Germany

**Keywords:** Multiple sclerosis, Multiple sclerosis

## Abstract

Multiple sclerosis (MS) affects 2.9 million people. Traditional classification of MS into distinct subtypes poorly reflects its pathobiology and has limited value for prognosticating disease evolution and treatment response, thereby hampering drug discovery. Here we report a data-driven classification of MS disease evolution by analyzing a large clinical trial database (approximately 8,000 patients, 118,000 patient visits and more than 35,000 magnetic resonance imaging scans) using probabilistic machine learning. Four dimensions define MS disease states: physical disability, brain damage, relapse and subclinical disease activity. Early/mild/evolving (EME) MS and advanced MS represent two poles of a disease severity spectrum. Patients with EME MS show limited clinical impairment and minor brain damage. Transitions to advanced MS occur via brain damage accumulation through inflammatory states, with or without accompanying symptoms. Advanced MS is characterized by moderate to high disability levels, radiological disease burden and risk of disease progression independent of relapses, with little probability of returning to earlier MS states. We validated these results in an independent clinical trial database and a real-world cohort, totaling more than 4,000 patients with MS. Our findings support viewing MS as a disease continuum. We propose a streamlined disease classification to offer a unifying understanding of the disease, improve patient management and enhance drug discovery efficiency and precision.

## Abstract (web summary)

Using large cohorts from published clinical trials involving more than 8,000 patients with multiple sclerosis, a probabilistic machine learning model reconstructs the transition probabilities from data-derived diseases statuses, showing patterns that suggest how progression to severe stages occur and potential inversion of the process.

## Main

MS is a debilitating disorder of the central nervous system (CNS), affecting approximately 2.9 million individuals worldwide (one in 3,000 people)^1,2^. MS course descriptors, defined and revised by clinical consensus^3,4^, have aided patient–physician dialogue in the clinic and the design and recruitment of clinical trials. These clinical disease course descriptors—relapsing-remitting MS (RRMS), secondary progressive MS (SPMS) and primary progressive MS (PPMS)—have had a major impact on drug development, as they were used to delineate trial populations, and on the practical management of people living with MS, as they define treatment indications and patient access to approved MS therapies. However, these traditional course descriptors are based on clinical presentations of the disease rather than on the underlying disease biology. Their value for prognostication is limited to the observation that patients with a progressive disease course (PPMS or SPMS) tend to have a worse prognosis than those with a relapsing-remitting disease course^5^, and responsiveness to treatments in the progressive spectrum of the disease depends on the presence of radiological disease activity rather than the subtype of MS^6^. Recent evidence from real-world studies and clinical trials shows that disease progression independent of relapse activity (PIRA) is common in RRMS^7–10^ and is associated with poor long-term prognosis^11^. Additionally, current MS course descriptors are categorical and mutually exclusive (with implications for patient access to approved medications), whereas, in reality, relapsing and progressive disease features often overlap^10^. Pathophysiological differences between relapsing and progressive MS are more quantitative than qualitative in nature, with many features associated with disease worsening, such as slowly expanding lesions, meningeal and compartmentalized inflammation, neuroaxonal injury and brain and spinal cord atrophy, being present from disease onset and shared between subtypes of MS^12–15^. This raises the important question of whether the current MS categorization into distinct subtypes is justified or whether MS would be better described as a disease continuum from a focal inflammatory to a progressive disease course.

**Extended Data Fig. 1:**
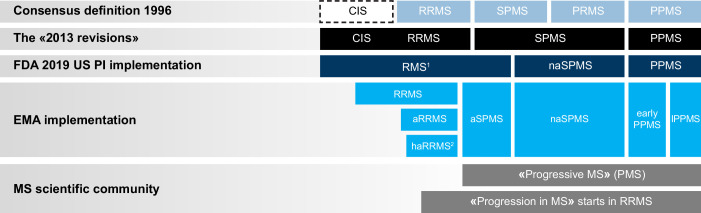
Disease classification of multiple sclerosis. Consensus definitions from 1996 and in its ‘2013 revisions’ and variants of it as used in indication statements in US packet inserts, summaries of product characteristics by the European Medicines Agency and in scientific publications. Relapsing forms of multiple MS include CIS, RRMS, and aSPMS in adults. aRRMS, active RRMS; aSPMS, active SPMS; CIS, clinically isolated syndrome; haRRMS, highly active RRMS; IPPMS, late PPMS (as opposed to ‘early PPMS’); MS, multiple sclerosis; naSPMS, non-active SPMS (as opposed to ‘active SPMS’); PMS progressive MS (SPMS + PPMS); PPMS, primary progressive MS; PRMS, progressive relapsing MS; RRMS relapsing remitting MS; SPMS, secondary progressive MS; «progression in MS » refers to the process of progression, which occurs in all subtypes of MS.

Over the last three decades, progress has been made in the development of efficacious therapies, which have markedly improved the outlook for people living with MS^10,16^. Current therapies are predominantly licensed for the relapsing phase of MS, with demonstrated benefits in progressive MS (SPMS and PPMS) mostly confined to patients with recent disease activity. Based on heterogeneous treatment effects observed within progressive subtypes of MS and benefit–risk considerations, regulators in the United States and Europe have created complex subclassifications to tailor indications (for example ‘active SPMS’ and ‘early PPMS’) in recent approvals, thereby deviating from the original consensus definitions (Extended Data Fig. 1). Such inconsistent views of MS pose challenges in the drug development process, especially in the progressive spectrum of the disease where there remains a high unmet medical need. Differences in definitions between jurisdictions and deviations from the consensus definitions also have the potential to create confusion in clinical practice in terms of patient access to approved treatments^17^. To address these fundamental issues, it is crucial to reevaluate the current MS categorization using a data-driven and evidence-based approach^15,18,19^.

With the goal of achieving a data-driven reclassification of MS, we adopted an AI-based approach, more specifically a bespoke probabilistic machine learning method, to reclassify the disease trajectories of more than 8,000 patients from the Novartis-Oxford MS (NO.MS) database, which is currently the largest and most comprehensive MS clinical trial database^20^. Our methodology—a scalable probabilistic factor analysis hidden Markov model (FAHMM) agnostic to the traditional MS subtypes—uses a probabilistic latent factor analysis (PFA) to represent multimodal clinical and radiological trial data. This approach simplifies complex data by capturing the correlation between variables into composite scores, which are then assumed to follow a hidden Markov model (HMM). By applying this model, we report homogeneous multivariate disease states and the transition probability matrix between these states, thus providing quantitative insights into transition pathways and a data-driven view of the evolution of MS over time. Drawing from our findings and existing literature, we propose a reclassification of MS, which offers an opportunity to unify the understanding of this disease with implications for drug development and the practical management of people living with MS.

## Results

### Discovery and validation

A total of 8,023 patients with up to 15 years of follow-up (118,235 visits) from nine phase 2/3 MS clinical trials in the NO.MS database were included in the main analysis. Diagnoses at trial entry were RRMS (*n* = 5,761), SPMS (*n* = 1,550) or PPMS (*n* = 712). Baseline characteristics largely overlapped across studies, with more disabled patients in progressive MS trials (Supplementary Fig. 1.1). The dataset was split into discovery (*n* = 6,419) and replication (*n* = 1,604) samples. Results were externally replicated in an independent Roche clinical trial dataset^21,22^ (*N* = 2,243; see Extended Data Table 1 for source studies) and a real-world cohort, Multiple Sclerosis Partners Advancing Technology and Health Solutions (MS PATHS)^23^ (*N* = 2,080), based on predefined validation criteria (Methods). Demographics and MS feature distributions are compared across datasets in Extended Data Table 2 and Supplementary Fig. 1.2.

### Four latent dimensions to characterize MS

**Fig. 1:**
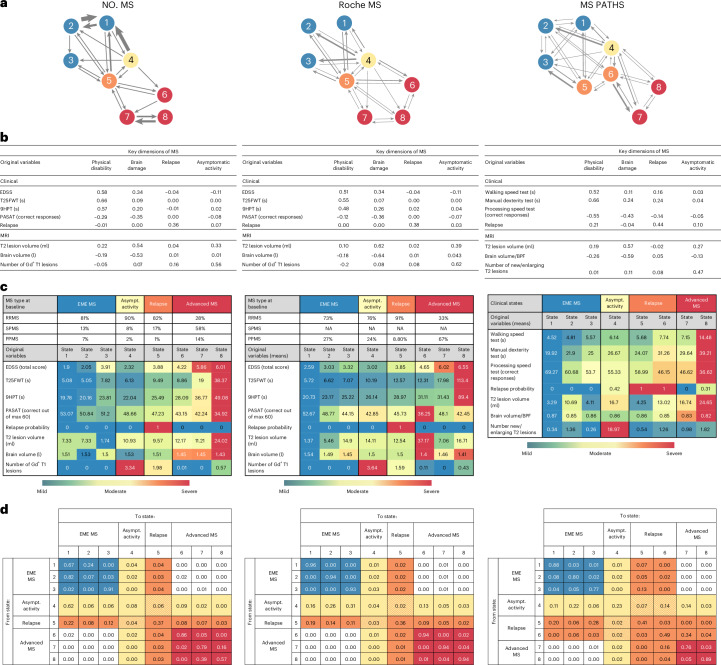
Disease evolution of MS based on the transition probabilities among the eight states of MS as proposed by the FAHMM model for NO.MS (main result), the independent validation dataset (Roche MS) and the real-world cohort (MS PATHS). **a**, Graphical summary of the eight statistical states of MS and the transition probabilities among them. **b**, Estimated loading matrices to identify ‘key dimensions of MS’. Bolded numbers refer to measures significantly (positively or negatively) associated with the dimensions based on the posterior probability of belonging to the slab component. Asymptomatic MS disease activity is identified based on the presence of Gd^+^ T1 lesions, in the absence of relapses. **c**, Descriptive summary of the percentage of patients with an MS subtype diagnosis (RRMS, SPMS or PPMS) and empirical means of the original variables characterizing the eight states; for more complete summary statistics, see Supplementary Table 2.1 and Extended Data Fig. 3. Note that, for MS PATHS, the diagnosis was self-reported by the patient and was missing for most (53%) and, therefore, not reported here. For a more detailed comparison of baseline features of the patients in NO.MS, Roche MS and MS PATHS datasets, see Supplementary Fig. 1.2 and Extended Data Table 2. **d**, Transition matrix between disease states as estimated by FAHMM, where each cell indicates the probability of a patient transitioning from their current state (row) to a subsequent state (column) over the course of 1 month. Patients may transition in any order between disease states. The thickness of the arrows in **a** is proportional to the probability of the transition between states as described in the transition probability matrix in **d**. In all figures, the color code refers to the clinical meta-states: blue indicates EME MS; yellow indicates asymptomatic MS disease activity; orange indicates relapse; and red indicates advanced MS. BPF, brain parenchymal fraction; Gd^+^, gadolinium-enhancing; s, seconds.

The FAHMM model, which uses latent variable modeling, is presented alongside the validation results in Fig. 1 and is illustrated in Fig. 1a. Probabilistic latent variable modeling of all clinical and radiological features measured in the clinical trials identified four latent dimensions of MS (Table 1): physical disability, brain damage, relapse and asymptomatic radiological activity. These were named based on clinical and radiological features in the loading matrix (Fig. 1b).

**Table 1:** Four dimensions, eight states and four clinical (meta-)states to characterize MS as identified by probabilistic latent variable analysis with a description of the patient features and transition probabilities

MS dimensions	Description	
Physical disability	Associated with the EDSS score, the T25FWT and the 9HPT, which are objective assessments of physical impairment.	
Brain damage (new)	Quantifies cumulative radiological disease burden, assessed by total T2 lesion volume and normalized brain volume on MRI. Brain damage, although associated with cognitive deficits and physical impairment, provides information about the integrity of the CNS not captured by the level of disability alone.	
Relapse	Describes whether a patient is in a relapse. The relapse can occur at any time; in our database, it was captured in unscheduled visits at the time patients experienced new or worsening neurological symptoms.	
Asymptomatic radiological disease activity (new)	Characterized by Gd-enhancing T1 lesions without patient-reported or physician-reported new or worsening symptoms. Our clinical trial databases (NO-MS and Roche MS) collect MRI scans at set times, regardless of symptoms, which is ideal for identifying and quantifying subclinical radiological disease activity. Asymptomatic radiological disease activity was frequently observed with varying levels of intensity across all traditional MS subtypes.	
**Clinical states of MS**	**Patient description**	**Transition probabilities**
EME (states 1–3), 59,810 instances observed^a^	Ambulatory MS patients with no or mild cognitive impairment. Clinically stable (that is, 100% not in a relapse, no or very few Gd lesions). Mean brain volume >1.5 l and mean T2 lesion volume <10 cm^3^. Mostly young, two-thirds female, most with an RRMS diagnosis. Notably, state 3 also comprises older ambulatory patients with longer-standing disease: 16% with a diagnosis of PPMS and 31% with a diagnosis of SPMS.	Patients in an EME state usually remain in an EME state with a high probability (>90%) from one visit to the next. Transition to state 4 or state 5 can occur by developing high disease activity, which can occur subclinically (state 4) or be accompanied by new or worsening neurological symptoms (relapse, state 5). Close to zero probability of direct transitions to advanced states (without going to state 4 or state 5).
Asymptomatic radiological MS activity (state 4), 3,709 instances observed^a^	Characterized by multiple Gd-enhancing T1 lesions in absence of any new or worsening clinical symptoms. Detected via contrast-enhanced MRI indicating blood–brain barrier breakdown. High subclinical activity is most typically seen in the youngest patients. Two-thirds female, >90% RRMS diagnosis. However, also seen in some SPMS (7.4%) and PPMS (2.4%) patients. Upper age quartile: 43 years.	Patients in state 4 typically come from an EME state (states 1–3) or from a previous inflammatory state (state 4 or state 5). Patients in state 4 are likely to remain in an inflammatory state (state 4 or state 5) or to recover to an EME MS state (states 1–3). However, direct transitions from state 4 to advanced MS (states 6–8) are possible with an 11% probability, indicating that asymptomatic MS disease activity poses a risk for further disease evolution.
Relapse (state 5), 5,504 instances observed^a^	State 5 defines the relapse as a distinct state of MS based on the presence of acute neurological symptoms. Mean of 1.98 Gd lesions during the relapse. Two-thirds were female, and 82% had a diagnosis of RRMS. However, 17% had a diagnosis of SPMS, and 1% had a diagnosis of PPMS. Although relapses occur most frequently early in MS in young and ambulatory patients, they can also happen at older ages (upper quartile at 47 years of age), in more advanced stages (upper quartile at EDSS 5) and late in the disease (upper quartile of MS disease duration: 21 years of MS).	Patients can experience relapses from any state of MS, most commonly after a previous relapse (that is, symptoms persist from one monthly visit to next, 37% probability^b^) or after asymptomatic activity (that is, patients who previously had Gd lesions develop symptoms, 6% probability). Relapses pose a risk for further disease evolution. Transitions from the relapse to advanced states of the disease are possible with 18% probability.
Advanced (states 6–8), 25,668 instances observed^a^	Characterized by moderate to high physical disability, cognitive deficits, high levels of brain damage and low focal inflammation. Mean T2 lesion volume, >10 cm^3^; brain atrophy, <1.5 l. On average, patients are 6 years older than those in the EME states with a more balanced sex ratio. Most (72%) have a diagnosis of progressive MS (either SPMS or PPMS) without distinction between SPMS and PPMS being made by the model.	Patients in advanced states experience moderate to high disability and brain damage. Recovery to earlier states is improbable, and patients usually remain in advanced states.

^a^Includes repeated counting of patients who were in one of these states on multiple occasions in our longitudinal dataset.

^b^In the NO.MS database, the average physician-reported duration of a relapse is 47 days, with many lasting up to 3 months or longer.

### MS disease states and evolution over time

**Extended Data Fig. 2:**
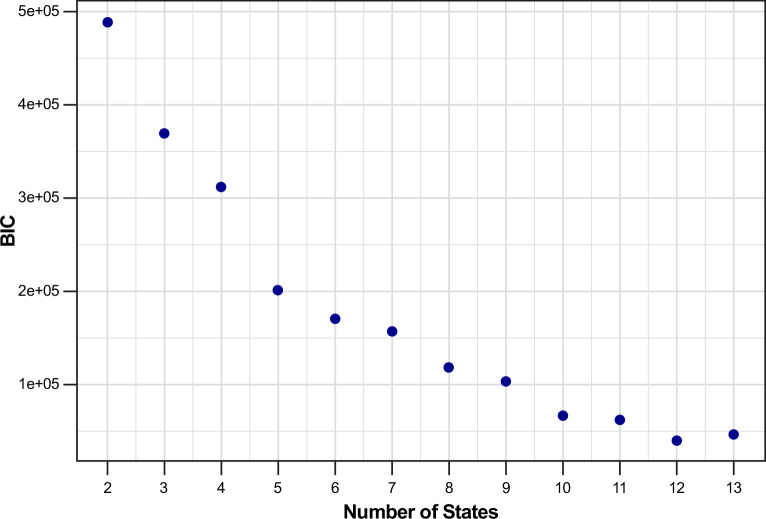
Selecting the number of states. BIC, Bayesian Information Criterion. A local minimum would indicate the optimal number of states. However, no single best number of states was identified. Models with more and more states with overlapping disease features (as illustrated in Extended Data Fig. 3) – representing a gradient in disease severity features – were found to be a better representation of the data than simpler models.

**Extended Data Fig. 3:**
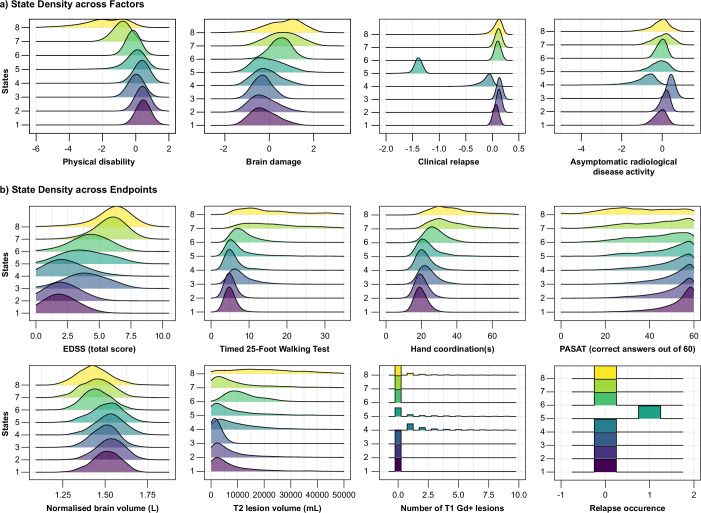
Graphical illustration of disease states and MS as a gradient. The states represent a gradient of disease severity based on physical disability and brain damage with distinct inflammatory states without accompanying symptoms (state 4) or with such symptoms, that is relapse (state 5). **a**, Density plots in the eight-state model: Latent factor distribution in the eight states. Overlapping distributions form a gradient of disease severity based on physical disability and brain damage. Distinct inflammatory states for the clinical relapse (state 5), and for asymptomatic lesions (state 4) **b**, Endpoint distribution of the original clinical and radiological variables in the eight states. EDSS, Expanded Disability Status Scale; Gd, gadolinium-enhancing; MS, multiple sclerosis; PASAT, Paced Auditory Serial Addition Test.

**Extended Data Fig. 4:**
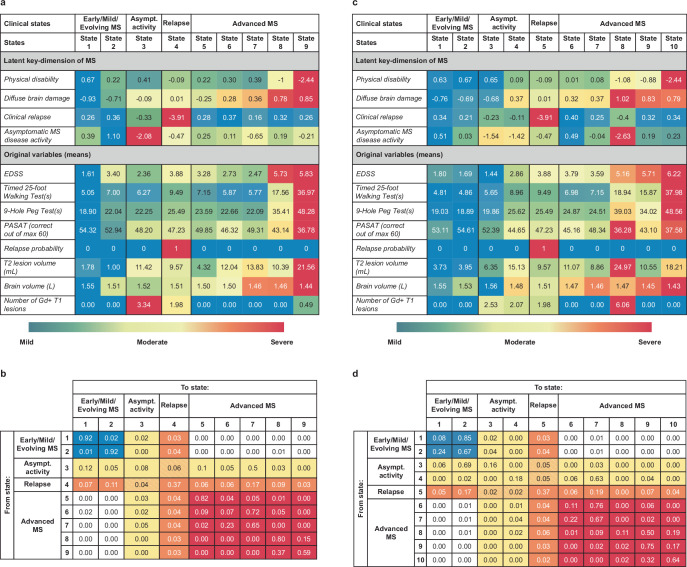
Alternative models with nine or ten states. **a, b**, model with nine states. **c, d**, model with 10 states. Composite score of MS dimensions and empirical means of the original variables characterising the states (**a** and **c**). Transition probability matrix from FAHMM (**b** and **d**). The transition probabilities refer to the probability of changing from one disease state to another one within a period of 1 month; the colour code refers to the clinical disease states as described in Fig. 1. Asympt., asymptomatic; EDSS, Expanded Disability Status Scale; Gd + , gadolinium-enhancing; PASAT, Paced Auditory Serial Addition Test.

The longitudinal composite scores of the four latent dimensions (from the probabilistic model) were used to identify disease states (Fig. 1c). The number of states was selected using the Bayesian information criterion (BIC; Extended Data Fig. 2), which favored models with more states over simpler alternatives, particularly over the traditional MS subtypes (RRMS, SPMS and PPMS), which would resemble a three-state model with a single one-way transition from RRMS to SPMS and PPMS as a distinct, static entity. The more complex models revealed overlapping feature distributions between states that, in fact, represent a disease severity gradient in terms of disability and brain damage and identified distinct states only for relapses and for periods during which patients have high asymptomatic radiological disease activity (Extended Data Fig. 3). Models with eight or more states had similarly low BIC and adequately represented the data. We present an eight-state model and its external validation in the main text in Fig. 1 (with feature distributions in Supplementary Table 2.1), and nine-state and 10-state variants are detailed in Extended Data Fig. 4 and Extended Data Tables 3 and 4. The disease states were successfully reproduced in the holdout data (Supplementary Fig. 3.1).

The FAHMM model provides a data-driven, probabilistic assessment of how individuals with MS transition between disease states over time. This dynamic aspect of the FAHMM model is captured in the transition probability matrix (Fig. 1a,d), which allows patients to remain in or move between states from one visit to the next. Patients may transition between disease states in any order. We did not restrict the transitions before fitting the model to the data, allowing the discovery of transition patterns in a data-driven manner (see Methods for details).

A key finding is that there is no direct monthly transition from states 1–3 to states 6–8; patients must first pass through one of the active states (4 or 5) (Fig. 1a,d). Furthermore, once in states 6–8, patients do not return to earlier states (1–3). Hence, solely based on those transition patterns observed in the NO-MS dataset, the eight states can be grouped into four clinical (meta-)states, which we named by consensus of the co-authors and the disease characteristics of the patients in these states as ‘EME’ (states 1–3), ‘asymptomatic radiological MS disease activity’ (state 4), ‘relapse’ (state 5) and ‘advanced’ state of MS (states 6–8). Table 1 provides a clinical characterization of the patients in each clinical (meta-)state of MS, along with the transition probabilities. A summary is as follows:

- EME MS (states 1–3) represents clinically stable, ambulatory patients with MS with low disability, minimal cognitive impairment and limited brain damage. Patients with EME MS are most likely to remain in an EME state from one visit to the next. Transitions to active states are possible by developing asymptomatic radiological activity (state 4) or by experiencing a relapse (state 5). Direct transition to advanced states of MS (6–8) without going to an activity state is highly unlikely (close to zero probability).
- Asymptomatic radiological MS disease activity (state 4): a radiologically active but clinically silent state, marked by multiple gadolinium (Gd)-enhancing lesions in the absence of reported new or worsening neurological symptoms. Most patients in this state are young and have a diagnosis of RRMS, but those with SPMS or PPMS also reach the asymptomatic radiological activity state, as revealed by our clinical trial datasets, in which magnetic resonance imaging (MRI) scans are collected at set times irrespective of symptoms. Patients often enter from EME states (1–3) or after a relapse (state 5). They may remain in this state, return to EME states (1–3) or relapse by developing acute neurological develop symptoms (state 5) or transition to advanced MS (states 6–8). Notably, there is a measurable risk (approximately 11%) of progressing via asymptomatic disease activity directly to advanced states (6–8) of MS, showing that subclinical disease activity is a risk factor for disease evolution.
- Relapse (state 5): characterized by acute new or worsening neurological symptoms—can occur from any state. Although relapses are more common early in the disease, they also occur at older ages and in patients with higher disability; they are seen most commonly in patients with a diagnosis of RRMS but are also experienced by patients with a diagnosis of SPMS or PPMS. A relapse is a risk factor for further disease evolution, as direct transitions from the relapse state to one of the advanced MS states (that is, state 5 to states 6–8) are possible with a 18% probability.
- Advanced MS (states 6–8) is defined by higher levels of physical and cognitive impairment, greater brain atrophy and reduced focal inflammation. Once in an advanced state, patients are unlikely to return to earlier states. Although relapses and lesions can still occur, transitions out of the advanced meta-state are rare; most patients remain within these states. The model does not differentiate between SPMS and PPMS in these advanced stages—that is, patients with SPMS and patients with PPMS are distributed with similar frequency across states 6–8.

Demographics and MS disease characteristics from NO.MS are provided for the four clinical (meta-)states in Table 2.

**Table 2:** Demographics and disease characteristics for the four clinical (meta-)states of MS based on NO.MS

Variable	EME MS	Asymptomatic radiological disease activity	Relapse	Advanced MS
	*n* = 59,810	*n* = 3,709	*n* = 5,594	*n* = 25,668
Demographics, MS subtype and disease duration				
Age				
Mean (s.d.)	41 (10)	37 (9)	40 (9)	47 (9)
Median (IQR)	41 (34–48)	36 (30–43)	40 (33–47)	48 (41–54)
Sex				
Female	41,029 (69%)	2,597 (70%)	3,984 (71%)	15,177 (59%)
Male	18,781 (31%)	1,112 (30%)	1,610 (29%)	10,491 (41%)
MS subtype				
RRMS	48,416 (80.9%)	3,345 (90.2%)	4,595 (82.1%)	7,215 (28.1%)
SPMS	7,513 (12.6%)	274 (7.4%)	937 (16.8%)	14,837 (57.8%)
PPMS	3,881 (6.5%)	90 (2.4%)	62 (1.1%)	3,616 (14.1%)
Years since first symptom				
Mean (s.d.)	12 (8)	10 (8)	13 (9)	17 (9)
Median (IQR)	9 (5–21)	8 (4–20)	9 (5–21)	20 (9–23)
Original MS measures				
EDSS (total score)				
Mean (s.d.)	2.44 (1.47)	2.32 (1.40)	3.88 (1.63)	5.26 (1.41)
Median (IQR)	2 (1.5–3.5)	2 (1.5–3.5)	4 (2.5–5)	6 (4.5–6.5)
T25FWT (s)				
Mean (s.d.)	5.87 (2.38)	6.13 (3.56)	9.49 (11.03)	18.45 (19.43)
Median (IQR)	5.25 (4.40–6.50)	5.20 (4.35–6.55)	6.15 (4.81–9.25)	11.90 (7.95–20.60)
9HPT (s)				
Mean (s.d.)	21.03 (4.45)	22.04 (5.45)	25.49 (10.88)	35.72 (18.38)
Median (IQR)	20.25 (18.08–23.18)	21.00 (18.50–24.15)	22.50 (19.52–27.12)	30.70 (25.68–39.06)
PASAT (number correct out of maximum 60)				
Mean (s.d.)	51.93 (9.04)	48.66 (10.88)	47.23 (12.11)	40.96 (14.15)
Median (IQR)	55 (48–59)	52 (43–57)	51 (40–57)	43 (30–53)
Volume T2 lesions (ml)				
Mean (s.d.)	5.84 (7.91)	10.93 (11.00)	9.57 (11.57)	16.18 (14.45)
Median (IQR)	2.92 (1.18–6.98)	7.58 (3.39–14.66)	5.38 (1.89–12.62)	12.07 (6.55–21.62)
Normalized brain volume (l)				
Mean (s.d.)	1.52 (0.08)	1.53 (0.09)	1.51 (0.09)	1.45 (0.09)
Median (IQR)	1.52 (1.46–1.58)	1.53 (1.47–1.58)	1.52 (1.45–1.57)	1.44 (1.38–1.51)
Number of Gd^+^ T1 lesions				
Mean (s.d.)	0.00 (0.00)	3.34 (5.22)	1.98 (5.05)	0.24 (0.97)
Median (IQR)	0 (0–0)	2 (1–3)	0 (0–2)	0 (0–0)

Demographic characteristics and MS variables are summarized by clinical disease states across all visits, counting patients each time that they were in a specific state; *n* represents the number of such visits to the specific state.

Gd^+^, gadolinium-enhancing; IQR, interquartile range.

The clinical (meta-)states were replicated in the independent external clinical trial and real-world datasets based on predefined validation (Methods) criteria as follows:

Validation in the Roche ocrelizumab phase 3 program (Roche MS) clinical trial dataset (*N* = 2,243) confirmed the four MS dimensions—physical disability, brain damage, relapse and asymptomatic disease activity—and showed that progression from EME to advanced MS occurred primarily through active states, despite the absence of patients with SPMS. The clinical interpretation aligned with the main findings and met validation criteria (Fig. 1).

Validation in the real-world MS PATHS cohort (*N* = 2,280). Despite using different measures (for example, new/enlarging T2 lesions, brain parenchymal fraction and no Expanded Disability Status Scale (EDSS)), lower-frequency assessments (6–12 monthly visits) and some patient-reported rather than physician-assessed measurements, the four MS dimensions and the EME-to-advanced disease gradient were also reproduced in the MS PATHS cohort. Transitions to advanced MS typically passed through active states, and direct worsening from early to advanced MS was confirmed to be rare. Minor differences emerged: only two advanced states were identified (versus three in NO.MS), and relapse was split into two relapsing states (one in earlier patients and one in more advanced patients). This is due to the fact that, in MS PATHS, which reflects real-world practice, visits were more symptom driven rather than scheduled independently, as is typically the case in clinical trials (Fig. 1).

#### Sensitivity analyses

**Extended Data Fig. 5:**
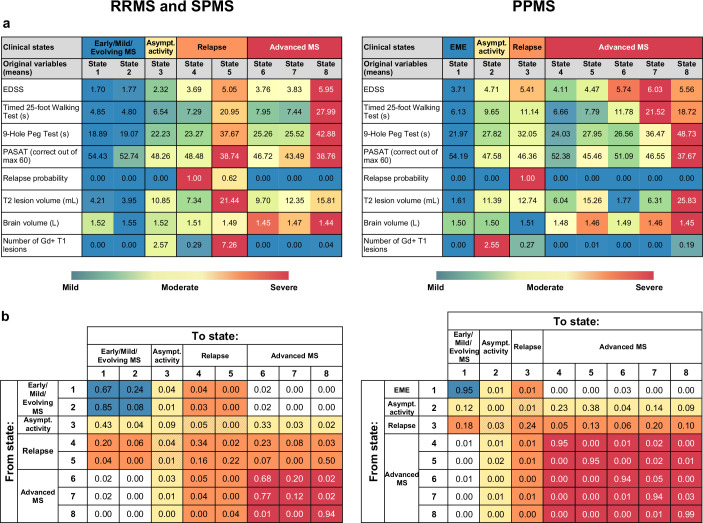
Eight-state modelling by MS subtypes. Clinical states (**a**) and transition matrices between states (**b**) for bout-onset MS (RRMS, SPMS) and PPMS separately. The disease severity gradient from EME to advanced states of MS, as well as the relapse and asymptomatic disease activity states, were re-discovered for PPMS, and similar to those observed for bout-onset MS. As a minor difference, only one EME state and several advanced states were discovered when fitting the model only to PPMS patients. This is expected, as studies in PPMS systematically excluded patients with an EDSS < 3.5. Overall, disease states and transition patterns were similar between RRMS-SPMS and PPMS patients.

An analysis for ‘bout-onset MS’ (RRMS and SPMS) and PPMS separately revealed similar disease states and transition patterns (Extended Data Fig. 5). The FAHMM requires complete data at each visit. In the main analysis, missing data were imputed only for partially observed visits, scheduled clinical visits and unscheduled relapse visits.

The missing data rates and mean absolute errors for imputed values on heldout data were as follows: Timed 25-Foot Walking Test (T25FWT): 32.3%, 0.14; 9-Hole Peg Test (9HPT): 43.6%, 0.17; Paced Auditory Serial Addition Test (PASAT): 53.9%, 0.30; T2 lesion volume: 72.5%, 0.08; brain volume: 69.9%, 0.08; and Gd-enhancing lesions: 73.1%, 0.08. These metrics suggest a reasonable level of imputation accuracy across variables.

**Extended Data Fig. 6:**
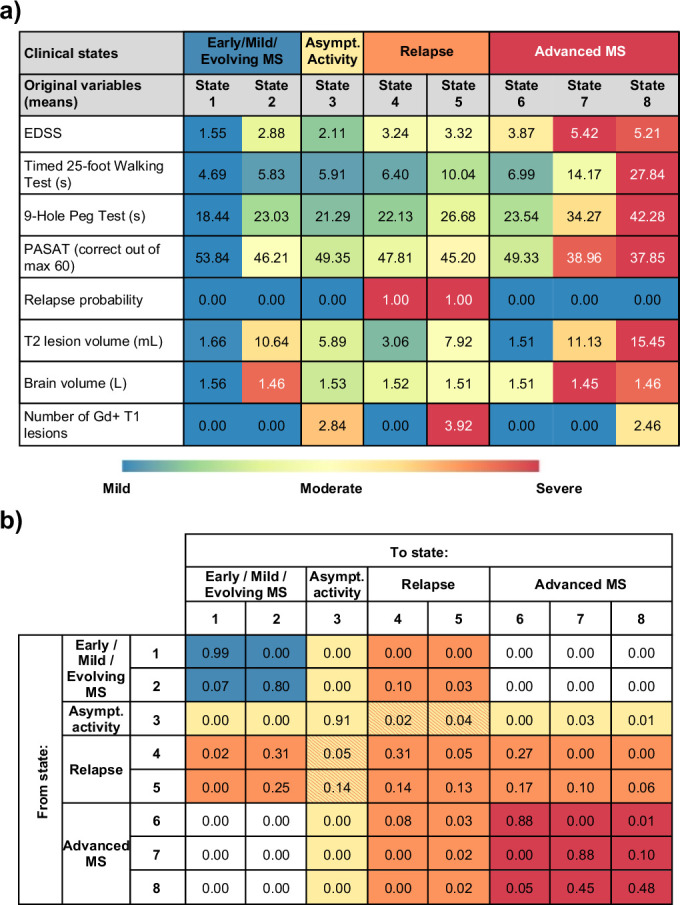
Eight-state modelling with no imputation of missing data. Analysis based on non-missing data, that is analysis without data imputation performed by mapping data to annual visits (based on the availability of annual MRI scans): (**a**) clinical states and (**b**) transition matrix between states. The frequency of subclinical disease activity is underestimated in this model due to the remapping of relapses from other timepoints were they occurred to the annual visits where MRI scans are available. Overall, the disease states and transition patterns observed were similar to those in the main model.

EDSS and relapse data were complete. Results of a further sensitivity analysis without data imputation, mapping available assessments to annual visits based on the availability of annual brain scans, are given in Extended Data Fig. 6. It showed an EME MS to advanced MS disease severity gradient with transitions either via relapses or asymptomatic radiological disease activity, consistent with the main model despite a substantial loss of longitudinal information (88.5% of all patient visits and 87.1% of the relapses were missed due to the remapping to annual visits).

### Progression independent of relapse activity by disease state

**Fig. 2:**
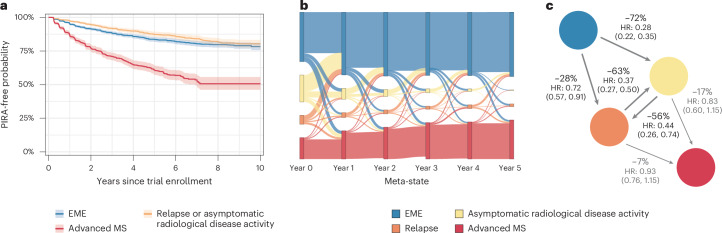
Disease progression and effect of treatments based on NO.MS. **a**, Time to first 3-month PIRA as a function of the clinical state in which the patient started at trial baseline. Kaplan–Meier estimates with shaded area representing 95% confidence intervals. **b**, Sankey plot of individual patient trajectories among the four clinical states of MS over a timeframe of 5 years. At year 0, patients are shown in the disease state in which they entered into a clinical trial: patients were in an EME state (blue), in a state of asymptomatic radiological disease activity (yellow), in a relapse (orange) or in an advanced state of MS (red). From left to right, the plot illustrates the proportion of patients who remain in the same disease state or move to another disease state. Please note that, for clarity of the graphic, only the yearly status of the patients is shown, and transitions between yearly visits are not displayed to avoid overcrowding the figure. If patients had relapses or radiological inflammation at these annual visits, this is correctly presented in orange and yellow, respectively. However, patients may have experienced relapses or asymptomatic radiological inflammation states between these annual points that contributed to their worsening, which cannot appear in this graphical representation; this explains why the figure displays blue connection lines between EME and advanced disease states in the figure even though the probability of a direct transition between EME and advanced states without passing through the inflammatory states is, in fact, low (see underlying transition matrix in Fig. 1d). **c**, Effect of (any) DMTs (versus placebo) on the transition probabilities among the four clinical states of MS. ‘Any DMT’ includes one of the following: interferon beta-1, glatiramer acetate, teriflunomide, fingolimod, siponimod or ofatumumab, which were compared to ‘no DMT’ (that is, placebo). The numbers refer to the percentage risk reduction (1 − HR, where HR refers to the hazard ratio between treated and untreated (placebo) patients and is reported with 95% confidence limits).

The time to the first PIRA event was analyzed by the disease state that patients were in at the baseline of a clinical trial (Fig. 2a). Patients starting in EME or active states (relapse or asymptomatic activity) had a lower risk and longer time to PIRA than those starting in advanced states, indicating that, although progression risk exists early, it increases markedly in advanced MS.

### Prognostication of individual trajectories

Individual patient journeys through the clinical disease states over 5 years are visualized in a Sankey plot (Fig. 2b). At baseline of the clinical trials, most patients are in an EME MS state (blue) or in an active disease state (yellow or orange); only a minority of patients are already in an advanced MS disease state (red), reflecting the composition of the NO.MS database and the eligibility criteria of the various trials. Patients transition between states annually, with both worsening and improvement observed. Each visit to an inflammatory state (relapse or asymptomatic activity) increases the risk of progressing to advanced MS. Over time, more patients accumulate disease burden and enter advanced states. Similar patterns were observed across RRMS, SPMS and PPMS (Supplementary Fig. 4.1), with consistent transition pathways from early to advanced states via inflammatory activity, regardless of MS subtype.

We evaluated the predictive performance of the proposed classification for the prognostication of an individual patient’s risk to transition into an advanced state of MS and the effect of disease-modifying therapies (DMTs) based on the estimated FAHMM model (Methods). The analysis revealed that, overall, the model predicts individual patient trajectories with high concordance in independent holdout data, with a good out-of-sample performance (C-score = 0.82, Brier score = 0.06).

### Impact of DMTs on MS disease evolution

Treating with a DMT significantly reduces the risk of patients with EME MS transitioning into the highly active asymptomatic MS disease state as well as their risk of transitioning into a relapse state (Fig. 2c). Compared to placebo, DMTs also lower the chance of remaining in an active state across visits. By interrupting the accumulation of damage to the CNS through these high-risk states, DMTs are associated with a higher probability of patients staying longer in the EME phase of MS.

## Discussion

We developed a data-driven classification of MS by applying bespoke probabilistic machine learning methodology to the longitudinal multimodal disease trajectories of more than 8,000 patients, covering all classical subtypes of MS. The main analysis in NO.MS is based on approximately 120,000 standardized neurological assessments and more than 35,000 MRI scans.

Our study was conducted in a clinical trial dataset with protocol-defined eligibility criteria as previously described^20^, resulting in a more narrowly defined population than typically encountered in clinical practice. We addressed this possible limitation by validating and reproducing our findings not only on a holdout dataset but also in an independent clinical trial dataset (Roche MS dataset)^21,22^ and in a real-world cohort (MS PATHS)^23^. The population included in our analysis is broadly similar to the NO.MS full dataset and to the real-world cohort based on reported demographics and disease features (Extended Data Table 2 and Supplementary Fig. 1.2). By including MS PATHS and Roche MS, we markedly expanded the range of treatments used.

The model is agnostic to the diagnosed clinical subtype of MS, allowing us to contrast it with the consensus-based MS course descriptors (Table 3). By contextualizing our findings within the traditional MS classification and existing literature, we propose revisions to the classification and recommend actions that could positively impact patient management and clinical trials alike.

**Table 3:** A comparison between the MS disease classification as proposed by the FAHMM model and the consensus-based clinical disease course descriptors

	Traditional course descriptors of MS^4^	FAHMM disease states
Dimensions to define MS subtypes/states	Two dimensions 1. Disability progression (mechanism) 2. Relapse	Four dimensions 1. Physical disability (absolute level) 2. Brain damage (reserve capacity) 3. Relapse 4. Asymptomatic radiological disease activity The first two dimensions describe a disease severity gradient; the third and fourth dimensions can move the patient along on this gradient.
Main classification	Distinct subtypes (static model) 1. RRMS 2. SPMS 3. PPMS The classical subtypes of MS can be denoted as a static three-state model with only a single transition from RRMS to SPMS possible.	Disease continuum (dynamic model) 1. EME MS 2. Asymptomatic radiological disease activity 3. Relapse 4. Advanced MS Patients move in and out of inflammatory disease states, thereby increasing the disease burden over time.
Modifiers of phenotypes	To express temporal status of patients 1. Inflammatory activity (MRI lesions or relapse) 2. Clinical progression No evidence of the quantitative impact of these disease course modifiers on the disease evolution has been provided in the consensus definitions^4^.	No modifiers needed (data-driven dynamic model of MS). Data from approximately 8,000 patients were used to estimate the transition pathways and probabilities between disease states in the form of a transition probability matrix between MS disease states, providing quantitative insights into MS disease evolution. These probabilities have been validated based on independent clinical trial data and real-world data from an additional more than 4,000 patients with MS.

Our FAHMM model results are more compatible with the view of MS as a disease continuum^10,13,15^ than with the traditional view of distinct phenotypes. A three-state model of static MS subtypes (RMS, SPMS and PPMS) was found to be inferior to more complex and dynamic models. In these dynamic models, the frequency of disease activity varies between individuals, explaining some of the individual differences in the accumulation of damage to the CNS and the acquisition of physical and cognitive impairment over time. Based on four dimensions—physical disability, radiological disease burden (that is, focal and diffuse brain damage), relapse and subclinical disease activity—and the transition matrix between disease states, the FAHMM has good performance for predicting disease course at the individual level (C-score > 0.8). The model thus enables prognostication of time to advanced MS based on the frequency of visits into one of the active disease states and the accumulation of disease burden. The results of this approach support the proposal by Lublin^24^ that the time framing—that is, the frequency of disease activity and progression within a set time window (for example, within a year)—should be considered in the practical management of MS.

The disease continuum was identified by our modeling in the form of multiple overlapping states that represent a gradient in terms of disease severity as defined by the level of disability and damage to the brain. Thus, the accumulation of a radiological disease burden is a manifestation of disease worsening, which can be reduced (or even prevented) with efficacious DMTs. We identified the meta-states EME MS (states 1–3) versus advanced MS (states 6–8) as two poles of a disease severity gradient. Models with more than eight states show greater resolution of the same gradient with no additional value for the clinical interpretation. Separate models for ‘bout-onset MS’ (RRMS and SPMS) versus PPMS were essentially similar.

The FAHMM results indicate that focal inflammatory and progressive forms of MS form a continuum in their phenotypic presentation. The model grouped RRMS, ‘active SPMS’ and ‘early PPMS’ together in the EME stages of MS without distinction, whereas fully evolved PPMS was grouped with SPMS in the advanced stages, also without distinction. The primary transition pathways from EME MS to advanced MS were through focal inflammatory states, either with accompanying neurological symptoms or stealthily via clinically silent radiological disease activity. The FAHMM considers the highly active disease states (4 and 5) as distinct disease states set apart from all other states by the occurrence of acute neurological symptoms or the unequivocal evidence of high levels of inflammation on the MRI scan (Gd-enhancing T1 lesions mean (s.d.): 3.34 (5.22)), respectively. This differs from the traditional view of MS that attributes lesions and relapses primarily to RRMS and is justified based on abundant evidence that lesions and relapses are not exclusive to any specific subtype of MS but can occur across the entire MS spectrum, including in patients with PPMS^10,20^.

The finding that ‘early PPMS’—that is, relatively young patients with low to moderate levels of disability and/or evidence of radiological disease activity—can benefit from anti-inflammatory treatment is consistent with subgroup results of the first trials of B-cell-depleting antibodies in PPMS^25,26^. More recently, a meta-analysis of 12 studies in the progressive spectrum of the disease (SPMS or PPMS) in a total of 8,659 patients showed that patients with recent disease activity could benefit from available DMTs, irrespective of whether their disease course was diagnosed as SPMS or PPMS, whereas patients without such recent disease activity could not^6^.

The 2013 revisions by Lublin et al.^4^ to the definition of the MS disease course introduced disease activity measures (MRI lesions and relapses) and progression as ‘modifiers’ of the classical static phenotypes (RRMS, SPMS and PPMS), without substantiating the quantitative relevance of such modifiers for disease evolution. Our FAHMM results enhance understanding of MS by offering a data-driven examination of the latent dimensions for disease characterization and a probabilistic quantification of the transition pathways between disease states. In our analysis, focal inflammation, with or without accompanying neurological symptoms, is the key driver of worsening. This finding was validated in the independent clinical trial dataset as well as in the real-world cohort. Our FAHMM shows that clinical and radiological assessments provide complementary information that differs from the initial consensus-based classification of MS, which focused on only two clinical dimensions (relapses and disability progression)^3^. It also differs from a more recent data-driven classification of MS that used only MRI data based on the premise that this would better reflect the pathophysiology of MS^27^. FAHMM results suggest that, in addition to the level of disability and relapse, two MRI-based latent dimensions of MS are relevant and should be considered for disease characterization and disease evolution:

1. Brain damage: assessed by the total T2 lesion volume and the level of brain atrophy as cumulative measures of focal and diffuse radiological disease burden. As the disease evolves, disease-related damage to the brain gradually accumulates, progressively lowering the patient’s chances for disability improvement^28^ and increasing the risk of further disability worsening^29^ and progression^30,31^. This decreasing ability to compensate and improve as the disease advances will similarly contribute to the clinical presentation of a progressive disease course, much like active drivers of worsening would, albeit likely through a different pathophysiological mechanism^28^. Thus, the accumulation of a radiological disease burden is a form of disease worsening, associated with poorer long-term outcomes; it should be minimized through the use of efficacious DMTs.
2. Asymptomatic radiological disease activity was revealed as a major pathway for patients to worsen, as shown by the FAHMM transition matrix. Our results from the much larger NO.MS dataset confirm the finding of a previous report by Thorpe et al.^32^, showing that more than 90% of the MS disease activity visible on regularly scheduled MRI scans (brain and spinal cord) is not accompanied by new or worsening neurological symptoms. High lesion activity is predominantly, but not exclusively, seen in young patients and occurs in all subtypes of MS^20^ and contributes to neuronal injury^33–35^ and brain tissue loss^36^ and, thereby, to the overall accumulation of damage to the brain. Enhancing lesions contribute to disease worsening in the same direction as clinical relapses^28^. Such damage is, in turn, associated with a lower chance of improvement and an increased risk of disease worsening and progression^28^ (see the FAHMM-proposed brain damage-related latent dimension of MS). Therefore, monitoring and preventing subclinical disease activity to protect reserve capacities should be a priority in MS management, considering that brain reserve influences how structural damage translates into clinical symptoms^37^. However, this approach may require novel strategies considering the current era of low relapse rates and safety concerns associated with regular use of Gd^38^.

Our FAHMM model confirms that PIRA starts early in MS and becomes the dominant feature in the advanced disease states when brain reserve is depleted, in line with the findings of Kappos et al.^9^ and Lublin et al.^10^. Patients in all states of MS can experience PIRA events, but the absolute risk of PIRA is higher in the advanced states compared to the EME states of the disease. In relative terms, PIRA is the most frequent manifestation of disability accumulation across the full spectrum of traditional MS phenotypes, as summarized in a recent literature review^39^. Perhaps surprisingly, FAHMM did not identify any direct pathway from EME to advanced states of the disease without passing through focal inflammatory states (4–5). Among the various biological mechanisms for MS evolution that have been proposed^40^, this finding supports the view that inflammation is central to the pathogenesis of MS and that degenerative processes in MS are secondary in nature. The risk of PIRA was estimated to be highest in the advanced states of MS, when the amount of damage to the CNS is typically substantial and response to currently available treatments is least likely.

Regarding the advanced states of MS, our results do not support maintaining a distinction between SPMS and PPMS; patients with SPMS and patients with PPMS were similarly distributed among the advanced states of the disease, both in the NO.MS dataset and in the real-world validation set. Once patients reach the advanced states of the disease, the risk of progression is high, and the chances of a treatment response are low, regardless of whether the previous disease course was characterized by relapses or not. Our results align well with the accumulating evidence that the apparent evolution from EME states to advanced states of the disease reflects a partial shift from predominantly localized acute injury to widespread chronic inflammation and secondary neurodegeneration^13,14,41–46^. Aging has a modulating effect; it decreases the likelihood of focal inflammation, increases neural susceptibility to injury and reduces resilience^15,20^. Abandoning the distinction between primary and secondary progressive MS in the advanced spectrum would acknowledge that these patients likely progress for the same reasons. Treating advanced PPMS and advanced SPMS as a uniform population would facilitate trial recruitment and drug discovery and would simplify drug indications and access in the progressive spectrum of the disease (that is, advanced MS is seen as one indication).

One may consider how the classical disease course descriptors (at presentation) would map to the FAHMM states and how this relates to current and potentially future disease classification. Radiologically isolated syndrome (RIS), which, in specific situations, is now MS^47^, would be detected based on asymptomatic lesions (state 4). Clinically isolated syndrome (CIS) would be identified based on a first clinical episode (state 5). Fully ambulatory patients with MS with limited damage to the brain who are not in a relapse and not in a highly inflammatory state would be considered in one of the EME disease states (1–3), irrespective of whether they have a relapsing or a progressive disease onset. Patients who have progressed in their disease, are impaired in their walking ability, have cognitive deficits and/or have accumulated a substantial lesion load or brain atrophy would likely qualify as being in an advanced disease state (6–8). It should be noted that, under the FAHMM, the combinations of physical and cognitive impairment and radiological damage to the brain that determine the classification of patients to a particular disease state can vary, reflecting inter-individual differences. Such differences render it impossible to provide simple thresholds that would demark movement between states and be applicable to all patients with MS. For the practical management of patients with MS and for the conduct of clinical trials, we propose it to be adequate and sufficient to consider the two ends of the disease continuum (EME MS versus advanced MS) while acknowledging that the shift in pathobiology is gradual rather than sudden, with considerable overlap of focal inflammatory and degenerative biology.

EME MS would include RIS, CIS and RRMS as well as ambulatory and cognitively functional patients with SPMS or PPMS who had recent imaging features characteristic of inflammatory activity. The primary goal would be to prevent relapses and asymptomatic disease activity by addressing the evolving disease pathology^15^. Meta-analyses suggest that such patients can benefit from currently available anti-inflammatory drugs^6,16,48^.

Advanced MS would include SPMS and PPMS (without distinction) and patients with RRMS who progressed in absence of relapse activity (PIRA) and who have moderate to high levels of disability with evidence of brain damage as shown by T2 lesion burden or evidence of disease-related brain atrophy. The primary goal would be to minimize the risk of further progression and to maintain or restore function. Secondary goals could be to minimize the risk of lesion expansion and brain atrophy. Developing treatments for advanced MS remains an urgent unmet medical need.

We summarized our recommendations for clinical practice and for clinical trials based on our findings and the literature in Table 4.

**Table 4:** Summary of recommendations for clinical practice and for clinical trials

Recommendation	Explanation
Define maintenance of brain integrity as a treatment goal in MS.	Our analysis identified focal and diffuse damage to the brain as a key dimension of MS. The analysis revealed that accumulating radiological disease burden is an aspect of disease worsening. As the disease evolves, disease-related damage to the brain gradually accumulates, progressively lowering the patient’s chances for disability improvement^28^ and increasing the risk of further disability worsening^29^ and progression^30,31^. Maintaining brain integrity by use of efficacious medications should be a treatment target.
Prioritize monitoring for subclinical disease activity.	Subclinical radiological disease activity was identified by our analysis as a dimension to characterize MS and as a major pathway by which patients worsen from EME MS states to advanced MS, both in our dataset and in real-world data. Monitoring for and prevention of such subclinical disease activity should be a priority in the treatment of MS. Effective monitoring of subclinical disease activity may require novel strategies given that the frequent use of contrast-enhancing MRI is discouraged for safety reasons^38^.
Consider MS as a disease continuum rather than distinct clinical subtypes, with EME MS and advanced MS as the two poles of this continuum.	This approach aligns with our results and the cumulative evidence indicating a gradual shift in pathobiology from predominantly focal inflammatory states to chronic inflammatory and degenerative conditions^12–15^.
EME MS would include RIS, CIS and RRMS as well as ambulatory patients with SPMS or PPMS who had recent imaging features characteristic of inflammatory activity.	Current DMTs reduce the probability of inflammatory events and slow disease worsening in EME states. Evidence suggests that patients with recent disease activity imaging features, including patients with SPMS with recent activity and patients with early PPMS, benefit from DMTs^6,51^.
Advanced MS would include SPMS and PPMS (without distinction) and patients with RRMS who progressed in absence of relapsing disease activity (PIRA) and who have moderate to high levels of disability with evidence of damage to the brain as shown by T2 lesion burden or evidence of disease-related brain atrophy.	We recommend discontinuing the distinction between primary and secondary progressive disease in the advanced spectrum of MS. We discovered that patients with SPMS and patients with PPMS are similarly distributed through the advanced disease states in clinical trial and real-world datasets. Pathophysiological evidence suggests that SPMS and PPMS are more alike than different and that progression, as a pathophysiological process, may be seen as a homogenous target for new medications focusing on the progressive aspects of MS. This could simplify drug discovery and the conduct of trials and facilitate drug access in an indication with a high unmet medical need.

We acknowledge the limitations in our dataset in terms of imaging frequency (typically only available in annual visits) and lack of advanced MRI features, including spinal cord imaging, cortical lesions and slowly expanding and paramagnetic rim lesions, which might account for some discordance between MRI disease activity and clinical outcomes. The overall limited data from spinal cord MRI in our database do not allow for definite or generalizable conclusions regarding its added value as a disease descriptor. Although there is evidence to show that spinal cord volume loss is significantly associated with present and future disability^49–54^, its potential added value over brain atrophy has not been studied or demonstrated. Regarding spinal cord lesions, based on a recent review^55^ systematic assessment of such lesions for monitoring of disease activity seems to have limited added value over monitoring of brain lesions and is considered optional based on current guidelines^38^.

To conclude, our model is a potentially important stepping stone to a data-driven disease characterization of MS. Although more comprehensive clinical information, such as spinal cord abnormalities or biological data, will be required before it can be considered definite, we think that if more advanced MRI measures and fluid biomarker data, such as proteomic or metabolomic data from banked samples, could be made available in this or any other large MS database, the FAHMM model would be suitable for a more in-depth pathophysiological description of MS and, provided availability of respective data, of any other diseases with similar complexities. With this comprehensive analysis, we hope to contribute to a more unified understanding of MS.

## Methods

### Analysis set from the NO.MS database

The NO.MS database was previously described^20^. In brief, it comprises 39 clinical trials from 2003 to April 2021, approved by institutional review boards (IRBs) or ethics committees (‘Ethics’ subsection and Supplementary Table 5.2) and conducted following the principles of the Declaration of Helsinki and Good Clinical Practice. All patients from all 39 trials provided written informed consent. Trial protocols prospectively defined the objectives, eligibility, endpoints, assessments and statistical analyses. The individual study results were previously published. Data were deidentified in a risk-based approach as reported elsewhere^56,57^. For this analysis, all phase 2 and 3 studies conducted in RRMS, SPMS or PPMS and their corresponding open-label extensions were selected based on the availability of protocol-defined standardized clinical assessments and regular MRI acquisitions. Studies contributing to the analysis are listed in Extended Data Table 1. In addition, an analysis of the wider NO.MS database, including all patients with clinical assessments of relapses and EDSS, investigated the risk of relapses and progression from pediatric MS to adult MS and from RRMS to SPMS and PPMS and identified a decreasing gradient of focal inflammation and an increasing gradient of the risk of progression^10^.

### Ethics

The ethics committees and IRBs used in the nine NO.MS source studies included: Alta Bates Summit IRB; Asahikawa Medical Center IRB; Ascension Wisconsin IRB; Aurora IRB; Baltimore IRB; Biomedical IRB; CentraState IRB; Central Ethics Committee; Chiba University Hospital IRB; Christiana Care IRB Helen F.; Copernicus Group IRB; Crescent City IRB; Dean IRB; Ebara Hospital IRB; Ehime University Hospital IRB; Georgetown University IRB; Health Sciences Institutional Review Boards; Health Sciences Campus IRB; Health System IRB; Healthcare -IRB; Henry Ford Hospital IRB; Hospital IRB; IRB University of California Davis; IRB of Beijing Hospital; IRB of West China Hospital; IRB-WB2; IRB/OSA; IRBMED; Institutional Ethics Committee, Bakirkoy; Institutional Ethics Committee, Dokuz Eylul; Institutional Ethics Committee, Ege; Institutional Ethics Committee, Gazi; Institutional Ethics Committee, Gaziantep; Institutional Ethics Committee, Hacettepe; Institutional Ethics Committee, Istanbul; Institutional Ethics Committee, Mersin; Institutional Ethics Committee, Uludag; Iwate Medical University Hospital IRB N/A; Johns Hopkins IRB; Keio University Hospital IRB; Kyoto Min-iren Chuo Hospital IRB; Lifespan IRB; Local Ethics Committee of AHEPA; Multicentric Ethics Committee IKEM; NIMS Institutional Ethics Committee; National Ethics Committee; Network IRB; Osaka University Hospital IRB; Pro Health Care IRB Research; Providence Health & Service IRB; Providence Health & Services IRB; Psychiatry IRB; Quorum Review IRB; Research Ethics Committee; Saitama Medical Center IRB; Schulman Associates, IRB; Sone Clinic IRB; The Ethics Committee of Sri; University IRB; University of Colorado Health IRB; University of Utah IRB; WIRB; WakeMed IRB; Wayne State University IRB; and Wheaton Franciscan Healthcare IRB N/A (see Supplementary Table 5.2 for full list and further details).

### Clinical assessments in NO.MS

For all the trials included in the NO.MS analysis set, the following clinical assessments, which are commonly used in MS clinical trials, have been regularly monitored (typically every 3 months or 6 months; for details, see the individual protocols and study designs) by specifically trained healthcare professionals:

- EDSS^58,59^: a standard tool for assessing the neurological disability status and disability progression, ranging from 0 (neurologically normal) to 10 (death due to MS).
- T25FWT^60^: an objective quantitative measure of neurological function (patient’s walking speed).
- 9HPT^60^: an objective quantitative measure of upper extremity (arm and hand) function.
- PASAT^61^: an objective measure of cognitive function that specifically assesses auditory information processing speed and flexibility as well as calculation ability.
- Relapse occurrence: defined as the appearance of a new neurological abnormality or worsening^62^, as experienced by the patients and reported by the study investigator. Patients who experienced new or worsening symptoms were instructed to come for an unscheduled visit where symptoms were assessed (with an EDSS assessment performed), and onset as well as end date were recorded by the physician. Patients were transferred to an EDSS rater (in phase 3 trials) and an independent physician for the EDSS assessment. In the present analysis, all patient-experienced and physician-reported new or worsening symptoms are considered, irrespective of the EDSS confirmation.

It should be noted that such visits could happen at any time and would typically occur in unscheduled visits between the regular scheduled visits. For this reason, to capture the timing of events adequately, a monthly grid was used for modeling purposes.

### Radiological assessments in NO.MS

In NO.MS, all images obtained according to study-specific standardized protocols were reanalyzed centrally by the Big Data Institute in Oxford, United Kingdom, using a harmonized MRI pipeline on standard MRI outcomes in MS (normalized brain volume using SIENAX^63^, part of FSL 6.0; percentage brain volume change using SIENA^64^, part of FSL 6.0). Gd-enhancing lesions and T2 lesion volume were used as reported in the original trials.

### Variables in NO.MS

The clinical and radiological variables used in our modeling are presented in Table 2, and their assessment is described in the previous two Methods subsections. Demographic and disease-related features are updated longitudinally. For each visit, the patient’s age is updated, and the normalized brain volume is calculated based on the normalized brain volume measured at baseline (using SIENAX^63^) and the percentage change from baseline measured at post-baseline visits (using SIENA^64^). Lesion assessments were done centrally as previously reported for each of the original trials.

Demographic features and the diagnosed phenotype of MS (RRMS, SPMS or PPMS) were not used in the modeling but are reported across visits for the disease states newly identified by the model to characterize the patients in a specific state and to help establish the link between the newly proposed FAHMM states and the traditional classification of MS. PIRA was derived as a 3-month EDSS-confirmed irreversible worsening of disability in the absence of relapses^10^.

### FAHMM

The proposed hierarchical model uses a PFA^65^ model to find a parsimonious representation of data. It exploits the shared information among elements of observed data to find MS dimensions (loading matrix) and corresponding composite scores (latent variables) that are continuous and a posteriori following a normal distribution. The spike and slab prior with Laplace components on the loading matrix favors sparsity that helps with the interpretation of MS dimensions. The number of MS dimensions is determined in a data-driven manner by putting an Indian buffet process prior on the inclusion/exclusion binary variables of spike and slab prior. Moreover, it helps with assigning observed variables to the MS dimensions by using a posterior probability of inclusion to the slab component greater than 0.5.

Next, our model assumes that the composite scores follow an HMM with multivariate normal emission distribution^66^. For modeling purposes, only the time gap between two consecutive visits is assumed to be 1 month (except for MS PATHS where it is assumed to be 6 months; see Supplementary Information Section 5 for more details). The HMM models MS evolution over time by (1) finding homogeneous disease states (latent unobserved) where the distribution of longitudinal composite scores is similar in terms of mean and covariance and (2) characterizing the progression between states by a transition probability matrix where all transitions are a priori possible (we are not restricting the transition probability matrix or assuming any structure). The number of states is determined by using the BIC.

Our proposed probabilistic multivariate model for disease evolution using longitudinal data is capable of handling mixed data modalities (binary, count and continuous) and missing data. The allocation of the observed variables to the MS dimensions does not change over time, which translates into a fixed loading matrix across visits. However, the composite scores are changing over time. The PFA uses baseline data where there are no missing data to estimate the loading matrix, and then the rest of the model parameters are estimated conditionally on the estimated loading matrix.

The model parameters are estimated using an expectation–maximization algorithm^67^. To evaluate the proposed classification’s predictive performance for the prognostication of an individual patient’s risk to transition into an advanced state of MS, the effect of DMTs and the characterization of individual states, the estimated FAHMM model is used to assign each visit to the corresponding disease and clinical states using the Viterbi algorithm^67^. The states are characterized by calculating the mean and s.d. of the corresponding variables. The disease states and transition probabilities are illustrated in Supplementary Fig. 5.1.

A discrete time-to-event analysis using Bayesian Additive Regression Trees (BART)^68^ was used to evaluate the prognostication performance of the clinical (meta-)states (Results). Time to first transition to one of the advanced states for patients who are in the early, relapse or asymptomatic states at baseline was predicted using baseline radiological and clinical features and demographic characteristics, including age, sex, treatment, relapses and number of relapses before entering the trial.

A continuous-time Markov model was used to assess the association between the use versus non-use of a DMT on the transition probabilities between clinical (meta-)states (msm package^69^).

### Replication in holdout data from NO.MS

The analysis was based on a total dataset of 8,023 patients that was randomly divided into a discovery set (6,419 patients) for analysis purposes and a holdout set (1,604 patients) for validation purposes. The *k*-means clustering method was used to identify a homogeneous group of patients using the average of longitudinal composite scores per patient. The clustering method found five different groups using the elbow approach, where 80% of patients in each group are randomly assigned to the discovery set and the remaining 20% to the validation set.

More methodological details can be found in Supplementary Information Section 5.

### Sensitivity analysis

A sensitivity analysis was conducted to check whether the disease states and transition pattern for ‘bout-onset MS’ (RRMS and SPMS) is similar to that of PPMS. A separate model was fit to RRMS/SPMS (excluding patients with PPMS) and to patients with PPMS alone.

Another sensitivity analysis was conducted without data imputation. This approach presents inherent complexities as the model requires complete data for all visits, whereas relapses can occur at any time, and other assessments are often unavailable at these timepoints. To conduct an analysis without data imputation, it was, therefore, necessary to remap all available data to annual visits based on the availability of MRI scans. This approach has the limitation that all data points collected between these annual visits are either ignored or shifted in time. After remapping the data to annual visits, the model was fit to these ‘complete case’ data.

### External validation on independent datasets

After submitting the initial version of this paper to *Nature Medicine* based on the NO.MS data, we sought to ensure the reproducibility and generalizability of our findings through validation using independent external datasets where we established predefined validation criteria prior to accessing these datasets. The model was then fitted to each external dataset, including an independent clinical trial dataset (Roche MS dataset) and a real-world cohort (MS PATHS), confirming the reproducibility of our results following the data preparatory steps described further in this section.

### Validation step 1: replication of MS dimensions

The PFA part of FAHMM^65^ uses baseline data to find MS dimensions (loading matrix) and corresponding composite scores (latent variables). The FAHMM model was fitted to all datasets where validation was evaluated by examining whether the same disease dimensions would emerge in the external datasets. Specifically, we determined whether the same or similar sets of variables from the primary analysis were assigned to the corresponding latent variables in the validation datasets. The validation of the latent dimensions of MS would be considered successful (validation criterion 1) if we could re-identify four dimensions related to (1) physical disability, (2) brain damage, (3) relapse and (4) asymptomatic MS disease activity.

### Validation step 2: replication of disease evolution modeling

In the main analysis, the MS evolution modeling using FAHMM discovered eight states that were grouped in four meta-states based solely on the patterns of the transition probability matrix using NO.MS data: EME MS, asymptomatic radiological MS disease activity, relapse and advanced MS. To replicate the main findings from the NO.MS dataset, we fit the FAHMM to either the Roche MS or the MS PATHS data with eight states as in the main model. A successful validation would entail finding meta-states with similar clinical interpretation and similar transition probability to NO.MS (validation criterion 2): the validation would be considered successful if we could re-identify an EME MS versus an advanced state of MS with a disease severity gradient and if the transition from the first to the second would primarily be through focal inflammatory disease states—that is, through a relapse or an asymptomatic radiological disease state—with little to no probability for patients to worsen from EME MS to advanced states without passing through these focal inflammatory states.

As described above, the validation focused on the qualitative similarity of the clinical interpretability rather than on numerical thresholds. By applying these predefined validation criteria to unseen data, we aimed to show the generalizability and robustness of our findings across independent datasets, including real-world data.

### Variables in external datasets

In the Roche MS dataset, the same clinical and radiological variables as in the NO.MS dataset were available. As in the NO.MS dataset, the clinical measurements were collected by trained neurologists, and MRI assessments (lesions and brain volume change) were measured by a central reading center. All assessments were used as reported in the original trials.

For the real-world dataset from MS PATHS, data assertation was different than in NO.MS and in Roche MS. Specifically, no EDSS assessments were available (patient determined disease steps (PDDS) measurements were collected rather than EDSS, but this was not used in the modeling). For most other variables in NO.MS, corresponding similar measures in MS PATHS could be identified: an iPad version of the 9HPT test was used (labeled as ‘manual dexterity test’); the walking test (noted as ‘walking speed test’) was found to be similar to the T25FWT; and an iPad version of a cognitive text (noted as ‘processing speed test’) similar to the Symbol Digit Modality Test (SDMT) was used. In MS PATHS, brain parenchymal fraction^70^ was calculated instead of normalized brain volume, and the number of new/enlarging T2 lesions was used instead of the number of Gd-enhancing T1 lesions. Other differences between MS PATHS and the clinical trial datasets were a lower frequency of visits (typically every 6–12 months) and the fact that visit occurrence was not independent of the occurrence of clinical symptoms (scanning frequency seemed to depend on the occurrence of relapses). Therefore, whereas, in the NO.MS and the Roche MS datasets, the transition probabilities refer to the probability of changing from one disease state to another one within a period of 1 month, in MS PATHS they refer to the probability of changing from one disease states to another within 6 months.

### Data preparatory steps

In each of the respective independent external validation datasets, visits were mapped to a regular grid to capture the timing of regularly scheduled visits as well as of unscheduled visits (for example, due to new or worsening neurological symptoms). For the Roche MS dataset, this was a monthly grid, similar to that of NO.MS, whereas, for MS PATHS, due to the lower visit frequency, this was a six-monthly visit grid (subsequently, probabilities in the transition matrix refer to monthly versus six-monthly transitions, respectively). To account for incomplete records and missing post-baseline data, the observed variable’s trajectory over time was used to impute missing values using generalized additive models. Such data imputation was done only at scheduled or unscheduled patient visits where partial patient data were available (Supplementary Information Section 5). The percentage of imputed values overall and for each variable is reported together with the mean absolute error for the imputation. In the clinical trial dataset, baseline was defined as the last assessment prior to randomization, whereas, in MS PATHS, baseline was defined as the first timepoint that patients had all the necessary measurements required for modeling; furthermore, the availability of serial post-baseline assessments was required for inclusion into the analysis set, which led to the total sample size of 2,080 patients from MS PATHS.

### Reporting summary

Further information on research design is available in the Nature Portfolio Reporting Summary linked to this article.

## Online content

Any methods, additional references, Nature Portfolio reporting summaries, source data, extended data, supplementary information, acknowledgements, peer review information; details of author contributions and competing interests; and statements of data and code availability are available at 10.1038/s41591-025-03901-6.

## Supplementary information

The supplementary file contains five sections as follows: Section 1 (Data sources): Fig. 1.1 and Fig. 1.2. Section 2 (Main model based on NO.MS): Table 2.1. Section 3 (Replication results in holdout NO.MS data): Fig. 3.1 and Table 3.1. Section 4 (Individual patient trajectories): Fig. 4.1. Section 5 (Methodology).

Reporting Summary

## Figures and Tables

**Extended Data Table 1:**
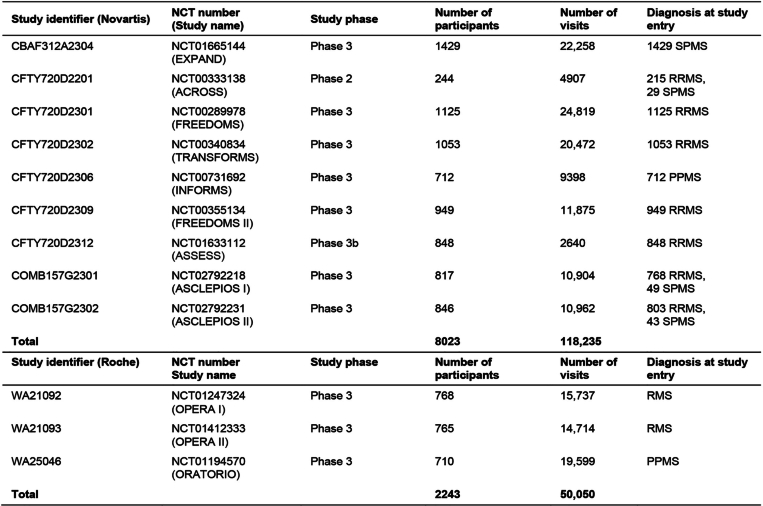
Number of participants and timepoints per study in NO.MS and Roche MS

For NO.MS, all longitudinal data from the same participants in corresponding extension studies are also included in the analysis. For Roche MS, all longitudinal data from the same participants in corresponding extension studies were also included in the analysis for ORATORIO. For OPERA I and II, the Multiple Sclerosis Functional Composite (MSFC) with its components T25FWT, 9HPT and PASAT were collected only in the core study but not the extension studies. Therefore, only the core phase from OPERA I and II could be used for the validation.

**Extended Data Table 2:**
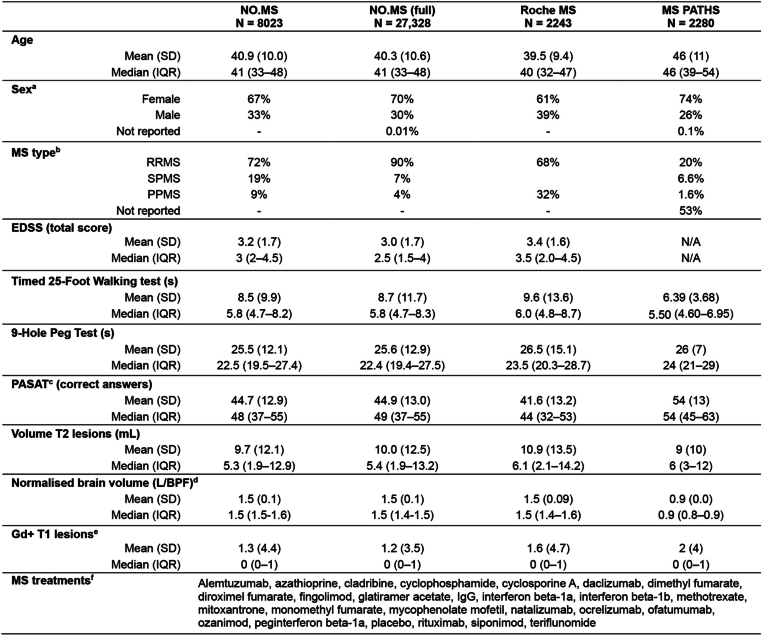
Demographics and baseline MS variables for the different datasets

^a^Two patients have missing data on sex in NO.MS (full) and three patients in MS PATHS.

^b^MS PATHS also includes patients with CIS (16%) and patients with progressive relapsing MS (2.5%). The RRMS value for Roche MS here is reported as RMS.

^c^PASAT was used in NO.MS and Roche MS (maximum of 60 correct answers); the cognitive test used for MS PATHS was similar to the SDMT.

^d^For MS PATHS, brain parenchymal fraction was calculated instead of the normalized brain volume.

^e^Number of Gd^+^ T1 lesions is not reported in MS PATHS, so the number of new/enlarging T2 lesions is used for this dataset instead.

^f^Fingolimod, glatiramer acetate, interferon beta-1, ofatumumab, siponimod, teriflunomide and placebo were used in NO.MS. Interferon beta-1a, placebo and ocrelizumab were used in the Roche MS dataset based on the ocrelizumab phase 3 program. All the active treatments listed were used in MS PATHS. BPF, brain parenchymal fraction; Gd^+^, gadolinium-enhancing; IQR, interquartile range.

**Extended Data Table 3:**
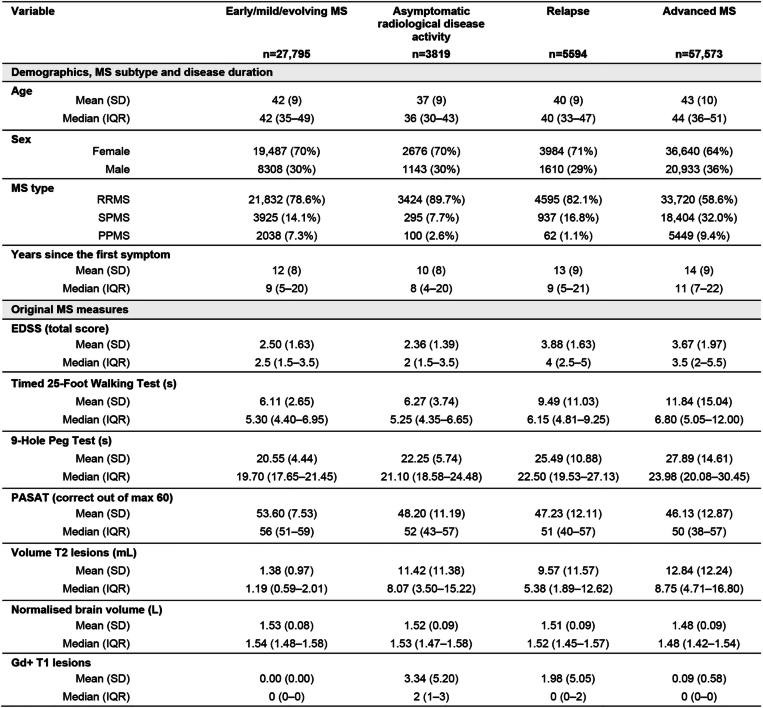
Demographics and disease characteristics for the nine-state model

Demographic characteristics and MS variables are summarized by clinical disease states across all visits, counting patients each time that they were in a specific clinical state; *n* represents the number of such visits to the specific state. Gd^+^, gadolinium-enhancing; IQR, interquartile range.

**Extended Data Table 4:**
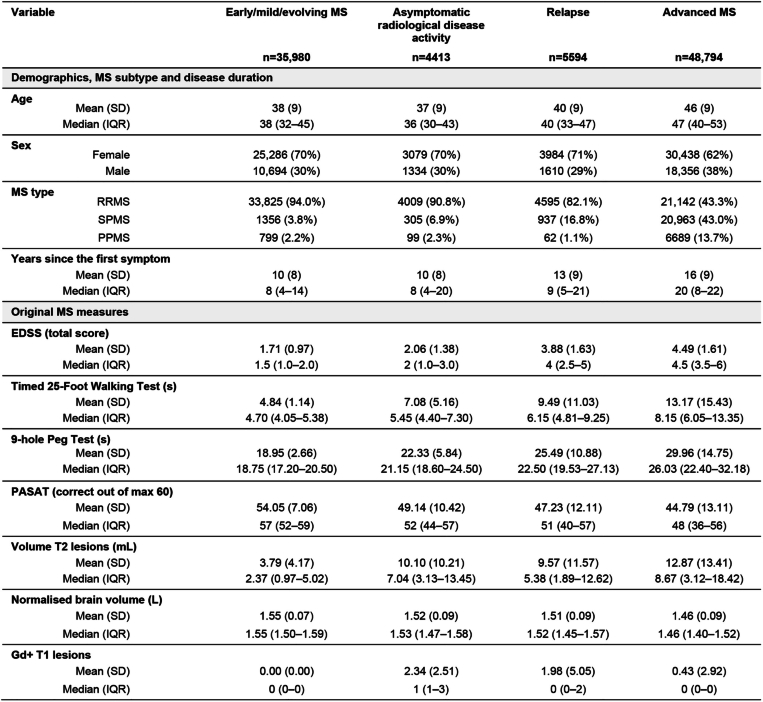
Demographics and disease characteristics for the 10-state model

Demographic characteristics and MS variables are summarized by clinical disease states across all visits, counting patients each time that they were in a specific clinical state; *n* represents the number of such visits to the specific state. Gd^+^, gadolinium-enhancing; IQR, interquartile range.

## Data Availability

For NO.MS, the reader is able to request the raw data (anonymized) and related documents (for example, protocol, reporting and analysis plan and clinical study report) of all the studies that underlie the modeling results reported in this article by connecting to CSDR (https://www.clinicalstudydatarequest.com) and signing a data-sharing agreement with Novartis. The data will be made available to researchers, with requests reviewed and approved by an independent review panel of CSDR. For Roche MS, including phase 3 ocrelizumab trial data used for the clinical trial validation, qualified researchers can request access to patient-level data by making a request via https://vivli.org/. The anonymized MS PATHS dataset used for the real-world validation can be obtained for purposes of replicating the findings of this study by contacting H.W. at heinz.wiendl@uniklinik-freiburg.de.

## Code availability

SIENA/X, part of FSL 6.0, available at https://fsl.fmrib.ox.ac.uk/fsl/, was used to derive normalized brain volume and percentage of brain volume change. The FAHHM code used in this work is available at https://github.com/habib61/FAHMM.
