# Supplementary material for: AI-driven reclassification of multiple sclerosis progression
Source: Nat Med. 2025 Aug 20;31(10):3414–24. doi: 10.1038/s41591-025-03901-6 (PMC12532606; doi:10.1038/s41591-025-03901-6)
Supplement: Supplementary file 1 — The supplementary file contains five sections as follows: Section 1 (Data sources): Fig. 1.1 and Fig. 1.2. Section 2 (Main model based on NO.MS): Table 2.1. Section 3 (Replication results in holdout NO.MS data): Fig. 3.1 and Table 3.1. Section 4 (Individual patient trajectories): Fig. 4.1. Section 5 (Methodology). [file 41591_2025_3901_MOESM1_ESM.pdf]

nature medicine

Supplementary information https://doi.org/10.1038/s41591-025-03901-6

AI-driven reclassification of multiple sclerosis progression

In the format provided by the authors and unedited

Supplementary material

Section 1: Data sources

The main analysis was done based on all the phase 2 and phase 3 trials in the Novartis-Oxford multiple sclerosis (NO.MS) database as summarized in the **Extended Data Table 2.1**. All the studies were approved approved by institutional review boards or ethics committees (**Supplementary Table 5.2**). The baseline features of patients in the trials from NO.MS database are compared in **Supplementary Figure 1.1**.

Our results were validated in an independent clinical trial dataset (Roche ocrelizumab phase 3 program [Roche MS]; N=2243; source studies listed in **Extended Data Table 2.1** and baseline features summarized in the **Extended Data Table 2.2)** and based on real-word data from the MS PATHS (Multiple Sclerosis Partners Advancing Technology and Health Solutions) database (N=2280). The comparison of the baseline features of the NO.MS database to these independent databases is provided in **Supplementary Figure 1.2**.

MS PATHS is a collaborative research network initiated and sponsored by Biogen. It focuses on collecting and analysing real-world data from patients with multiple sclerosis (MS) to advance the understanding and treatment of the disease. The project involves various sites across the United States and Europe, with over 20,000 patients enrolled. For the external validation of this work, only patients who had clinical and radiological data for the relevant parameters at multiple visits could participate, which reduced the number of patients to N=2280. Data assertation differed in MS PATHS from that in the NO.MS database or the Roche MS dataset; in particular, patients had no EDSS assessments in MS PATHS, and the majority of patients had no assessments for gadolinium (Gd)-enhancing lesions. For the external validation, we chose the closest proxies in the MS PATHS database to those in the clinical trial datasets. These were: the walking speed test (similar to the Timed 25-Foot Walking Test), the manual dexterity test (similar to the 9-Hole Peg Test), a cognitive test (similar the Symbol Digit Modalities Test), patient-reported relapses, the T2 lesion volume, the brain parenchymal fraction (as a proxy for the normalised brain volume) and the number of new or enlarging lesions (as a proxy for Gd-enhancing lesions).

Fig. 1.1 Comparison of baseline features between trials in NO.MS

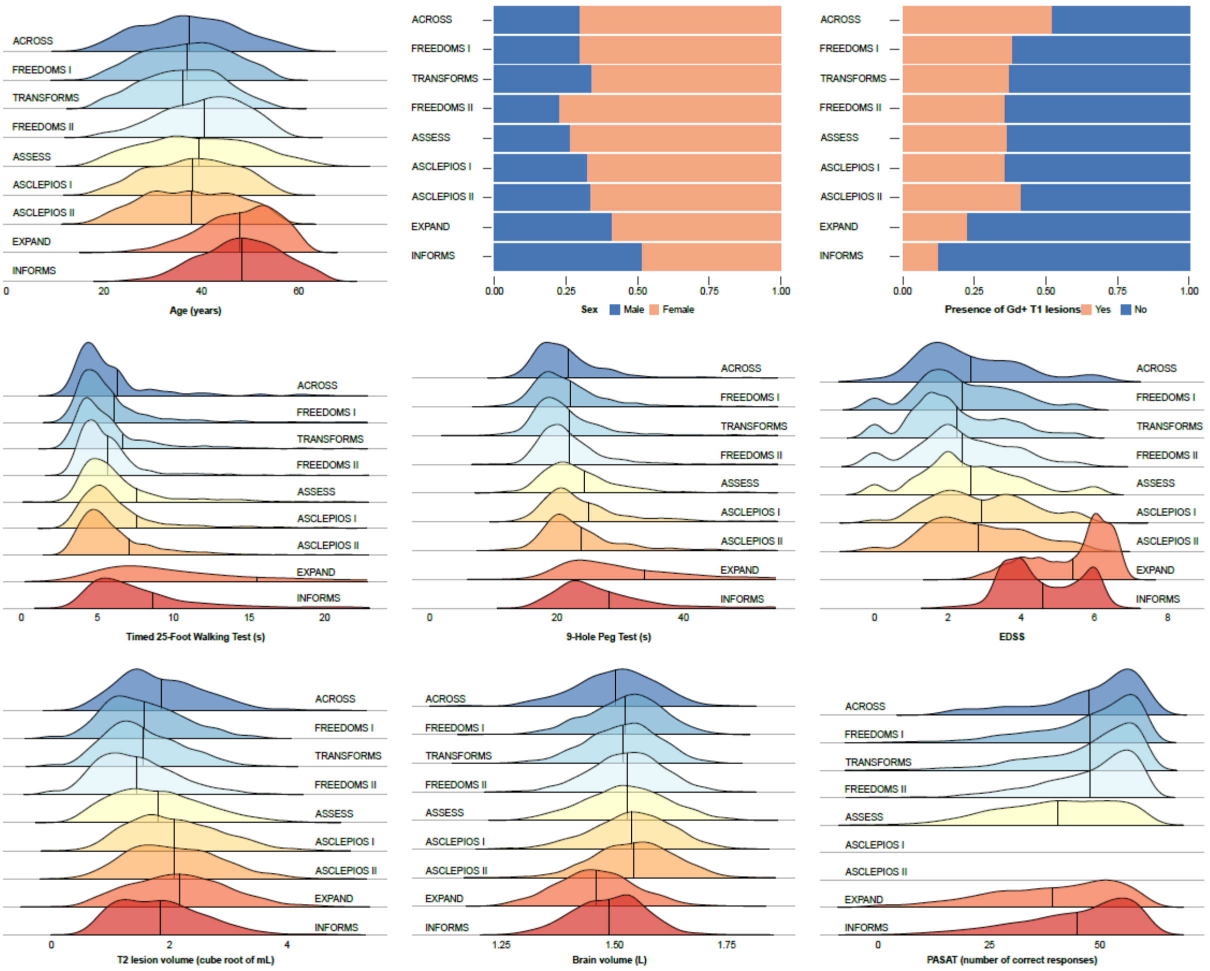

Study NCT numbers from top to bottom are: NCT00333138, NCT00289978, NCT00340834, NCT00355134, NCT01633112, NCT02792218, NCT02792231, NCT01665144, NCT00731692.

NO.MS, Novartis-Oxford MS dataset

Fig. 1.2 Comparison of baseline features of NO.MS with independent clinical trial and real-world datasets

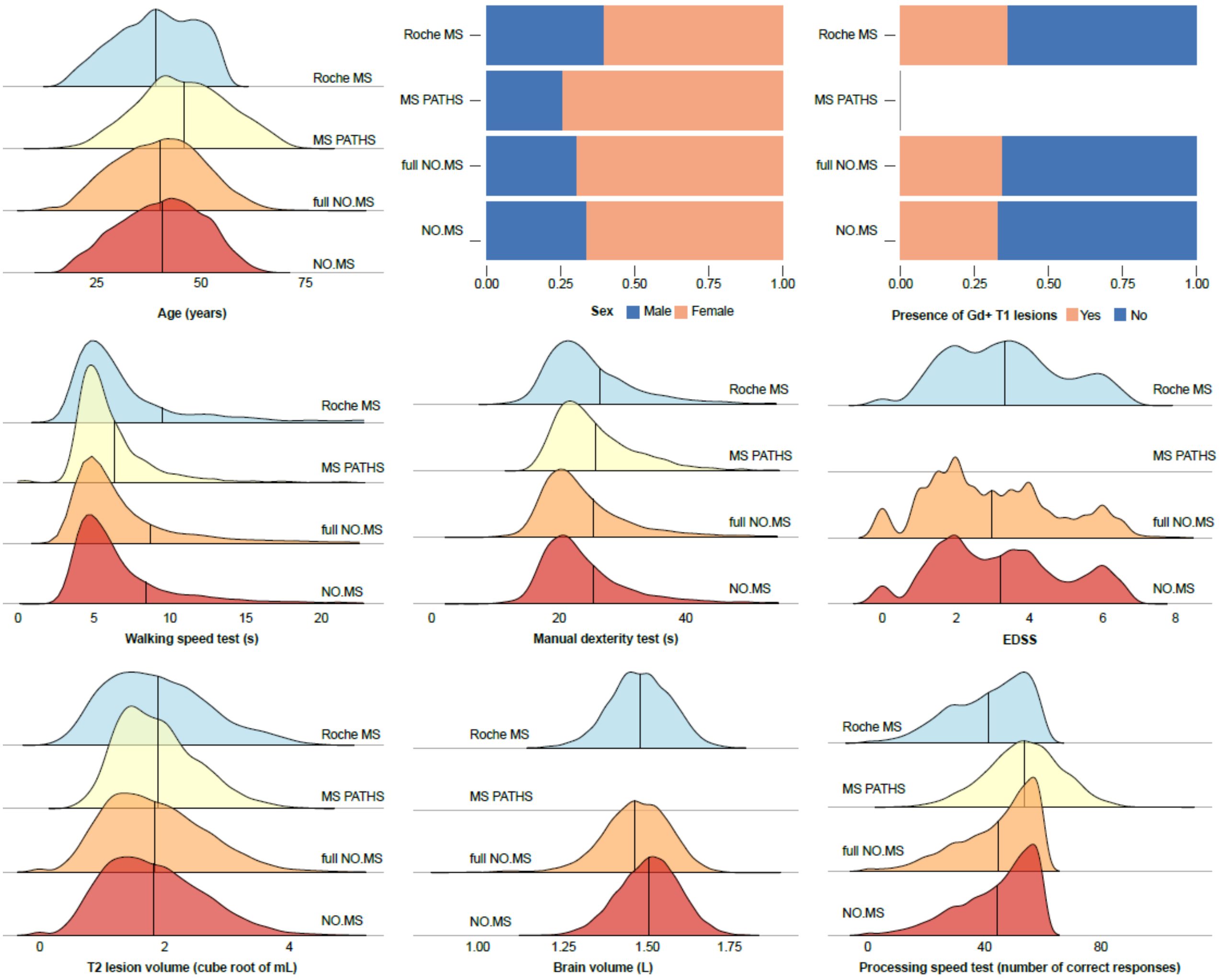

NO.MS is the dataset used for the main results (N=8023); full NO.MS considers all studies that reported relapses and collected EDSS consistent with the dataset previously reported Lublin et al., 2022 (N=27,328); Roche MS refers to the ocrelizumab phase 3 program used for independent validation in a clinical trial dataset (N=2243). MS PATHS is the dataset used for independent validation in real-world data (N=2280); EDSS scores, gadolinium-enhancing T1 lesions and normalized brain volume assessments were not captured in MS PATHS.

EDSS, Expanded Disability Status Scale; Gd+, gadolinium enhancing; MS. Multiple sclerosis; MS PATHS, Multiple Sclerosis Partners Advancing Technology and Health Solutions; NO.MS. Novartis-Oxford MS.

Section 2: Main model based on NO.MS

Table 2.1 The eight statistical states of MS, summaries of demographics and disease characteristics

Variable	State 1 n=33,512	State 2 n=11,045	State 3 n=15,253	State 4 n=3709	State 5 n=5594	State 6 n=9802	State 7 n=11,007	State 8 n=4859
**Demographics, MS subtype and disease duration**								
**Age**								
Mean (SD)	39 (9)	39 (9)	47 (9)	37 (9)	40 (9)	46 (9)	48 (9)	47 (9)
Median (IQR)	39 (32–46)	39 (32–46)	47 (41–53)	36 (30–43)	40 (33–47)	47 (40–53)	49 (42–55)	47 (41–54)
**Sex**								
Female	22991 (69%)	7702 (70%)	10,336 (68%)	2597 (70%)	3984 (71%)	5924 (60%)	6361 (58%)	2892 (60%)
Male	10521 (31%)	3343 (30%)	4917 (32%)	1112 (30%)	1610 (29%)	3878 (40%)	4646 (42%)	1967 (40%)
**MS Type**								
RRMS	30,390 (90.7%)	10,039 (90.9%)	7987 (52.4%)	3345 (90.2%)	4595 (82.1%)	4763 (48.6%)	1670 (15.2%)	782 (16.1%)
SPMS	2129 (6.3%)	587 (5.3%)	4797 (31.4%)	274 (7.4%)	937 (16.8%)	3686 (37.6%)	7596 (69.0%)	3555 (73.2%)
PPMS	993 (3.0%)	419 (3.8%)	2469 (16.2%)	90 (2.4%)	62 (1.1%)	1353 (13.8%)	1741 (15.8%)	522 (10.7%)
**Years since first symptom**								
Mean (SD)	11 (8)	10 (8)	14 (9)	10 (8)	13 (9)	16 (9)	18 (9)	19 (9)
Median (IQR)	9 (5–20)	8 (4–20)	11 (8–22)	8 (4–20)	9 (5–21)	20 (8–22)	21 (10–23)	21 (10–23)
**Original MS measures**								
**EDSS (total score)**								
Mean (SD)	1.90 (1.07)	2.05 (1.11)	3.91 (1.48)	2.32 (1.40)	3.88 (1.63)	4.22 (1.32)	5.86 (0.90)	6.01 (1.29)
Median (IQR)	2 (1.0–2.5)	2 (1.5–3.0)	4 (3.0–5.0)	2 (1.5–3.5)	4 (2.5–5.0)	4 (3.5–5.0)	6 (5.5–6.5)	6.5 (6.0–6.5)
**Timed 25-Foot Walking Test (s)**								
Mean (SD)	5.08 (1.29)	5.05 (1.26)	7.82 (3.20)	6.13 (3.56)	9.49 (11.03)	8.86 (3.76)	19.00 (10.69)	38.37 (35.77)
Median (IQR)	4.90 (4.20–5.70)	4.85 (4.15–5.70)	7.00 (5.60–9.10)	5.20 (4.35–6.55)	6.15 (4.81–9.25)	7.95 (6.35–10.25)	16.30 (10.95–24.75)	23.25 (11.50–57.04)
**9-Hole Peg Test (s)**								
Mean (SD)	19.78 (3.33)	20.16 (3.53)	23.81 (5.46)	22.04 (5.45)	25.49 (10.88)	28.09 (6.40)	36.77 (13.42)	49.08 (30.72)
Median (IQR)	19.35 (17.50–21.70)	19.68 (17.75–22.08)	22.85 (20.13–26.38)	21.00 (18.50–24.15)	22.50 (19.53–27.13)	27.13 (23.82–31.40)	33.35 (27.73–42.50)	38.73 (29.89–56.16)
**PASAT (correct out of max 60)**								
Mean (SD)	53.07 (8.38)	50.84 (9.73)	51.20 (9.12)	48.66 (10.88)	47.23 (12.11)	43.15 (12.67)	42.24 (13.92)	34.92 (15.35)
Median (IQR)	56 (50–59)	54 (47–58)	54 (47–58)	52 (43–57)	51 (40–57)	46 (34–54)	45 (31–54)	35 (24–48)
**Volume T2 lesions (mL)**								
Mean (SD)	7.33 (8.36)	7.33 (8.84)	1.74 (1.44)	10.93 (11.00)	9.57 (11.57)	12.17 (6.18)	11.21 (13.23)	24.02 (17.85)
Median (IQR)	4.34 (1.76–9.65)	4.08 (1.76–9.20)	1.33 (0.61–2.49)	7.58 (3.39–14.66)	5.38 (1.89–12.62)	10.93 (7.55–15.78)	6.45 (2.57–14.91)	20.75 (11.29–33.07)
**Normalised brain volume (L)**								
Mean (SD)	1.51 (0.08)	1.53 (0.08)	1.50 (0.08)	1.53 (0.09)	1.51 (0.09)	1.45 (0.08)	1.45 (0.09)	1.43 (0.10)
Median (IQR)	1.51 (1.45–1.57)	1.53 (1.48–1.59)	1.50 (1.45–1.55)	1.53 (1.47–1.58)	1.52 (1.45–1.57)	1.45 (1.39–1.51)	1.45 (1.39–1.51)	1.43 (1.37–1.49)
**Gd+ T1 lesions**								
Mean (SD)	0.00 (0.00)	0.00 (0.00)	0.00 (0.00)	3.34 (5.22)	1.98 (5.05)	0.01 (0.08)	0.00 (0.05)	0.57 (1.43)
Median (IQR)	0 (0–0)	0 (0–0)	0 (0–0)	2 (1–3)	0 (0–2)	0 (0–0)	0 (0–0)	0 (0–1)

Demographic characteristics and MS variables are summarised by states across all visits, counting patients each time they were in a specific state; n represents the number of such visits to the specific state. EDSS, Expanded Disability Status Scale; Gd+, gadolinium-enhancing; IQR, interquartile range; MS, multiple sclerosis; PASAT, Paced Auditory Serial Addition Test; PPMS, primary progressive multiple sclerosis; RRMS, relapsing-remitting multiple sclerosis; SD, standard deviation; SPMS, secondary progressive multiple sclerosis.

Section 3: Replication results in hold-out NO.MS data

Fig. 3.1 Eight-state model based on the hold-out data (replication samples from NO.MS)

a

Clinical states	Early/Mild/Evolving MS			Asympt. activity	Relapse	Advanced MS		
States	State 1	State 2	State 3	State 4	State 5	State 6	State 7	State 8
**Latent key-dimension of MS**								
Physical disability	0.66	0.52	0.21	0.49	-0.04	-0.44	-0.48	-2.37
Brain damage	-0.53	-0.53	-0.25	-0.34	-0.06	0.70	0.83	0.61
Relapse	0.19	0.35	0.27	-0.35	-4.07	0.42	0.26	0.21
Asymptomatic MS disease activity	-0.01	0.52	0.35	-1.81	-0.50	0.44	-0.07	-0.23
**Original variables (means)**								
EDSS	1.71	2.14	3.26	2.02	3.72	4.74	4.88	5.84
Timed 25-foot Walking Test (s)	4.88	5.26	6.85	6.41	8.91	10.41	11.89	36.78
9-Hole Peg Test (s)	19.12	20.20	23.23	21.58	24.93	29.52	30.40	49.65
PASAT (correct out of max 60)	53.75	50.71	49.68	48.56	46.70	40.28	43.29	40.37
T2 lesion volume (mL)	4.89	4.79	3.77	9.70	11.83	16.08	12.36	21.85
Brain volume (L)	1.51	1.53	1.48	1.54	1.51	1.45	1.43	1.46
Number of Gd+ T1 lesions	0.00	0.00	0.00	3.76	2.75	0.00	0.01	0.98
Relapse probability (%)	0.00	0.00	0.00	0.00	1.00	0.00	0.00	0.00

Mild Moderate Severe

b

From state:			To state:							
			Early/Mild/Evolving MS			Asympt. activity	Relapse	Advanced MS		
			1	2	3	4	5	6	7	8
	Early/ Mild/ Evolving MS	1	0.63	0.24	0.06	0.04	0.03	0.00	0.00	0.00
		2	0.64	0.09	0.22	0.01	0.03	0.00	0.00	0.00
		3	0.13	0.18	0.56	0.04	0.04	0.01	0.03	0.00
	Asympt. activity	4	0.49	0.07	0.20	0.11	0.04	0.01	0.08	0.00
	Relapse	5	0.20	0.09	0.09	0.04	0.37	0.04	0.15	0.03
	Advanced MS	6	0.00	0.00	0.02	0.00	0.03	0.11	0.80	0.03
		7	0.00	0.00	0.00	0.00	0.03	0.18	0.72	0.05
		8	0.00	0.00	0.00	0.00	0.02	0.02	0.15	0.81

a, composite score of MS dimensions and empirical means of the original variables characterising the eight states **b**, transition probability matrix from FAHMM. The transition probabilities refer to the probability of changing from one disease state to another one within a period of 1 month; the colour code refers to the clinical disease states as described in **Fig. 1.**

Asympt., asymptomatic; EDSS, Expanded Disability Status Scale; Gd+, gadolinium-enhancing; PASAT, Paced Auditory Serial Addition Test.

Table 3.1 Demographics and disease characteristics for the four clinical (meta-) states of MS based on the hold-out data (replication samples from NO.MS)

Variable		Early/evolving MS	Asymptomatic radiological disease activity	Relapse	Advanced MS
		n=12,779	n=913	n=1272	n=8490
**Demographics, MS subtype and disease duration**					
**Age**					
	Mean (SD)	40 (10)	36 (9)	39 (10)	48 (9)
	Median (IQR)	41 (33–47)	36 (29–43)	39 (31–46)	48 (41–54)
**Sex**					
	Female	8711 (68%)	677 (74%)	923 (73%)	4820 (57%)
	Male	4068 (32%)	236 (26%)	349 (27%)	3670 (43%)
**MS type**					
	RRMS	10,934 (85.6%)	845 (92.5%)	1055 (82.9%)	2654 (31.3%)
	SPMS	1279 (10.0%)	49 (5.4%)	205 (16.2%)	4684 (55.1%)
	PPMS	566 (4.4%)	19 (2.1%)	12 (0.9%)	1152 (13.6%)
**Years since first symptom**					
	Mean (SD)	12 (8)	10 (8)	12 (9)	17 (9)
	Median (IQR)	9 (5–20)	8 (4–20)	9 (5–20)	20 (9–22)
**Original MS measures**					
**EDSS (total score)**					
	Mean (SD)	2.14 (1.21)	2.02 (1.31)	3.72 (1.62)	5.04 (1.43)
	Median (IQR)	2 (1.5–3.0)	2 (1.0–3.0)	3.5 (2.5–5)	5.5 (4.0–6.0)
**Timed 25-Foot Walking Test (s)**					
	Mean (SD)	5.41 (1.61)	6.41 (4.23)	8.91 (9.82)	15.85 (16.61)
	Median (IQR)	5.05 (4.35–6.00)	5.25 (4.40–6.50)	6.05 (4.80–8.75)	10.30 (7.25–17.35)
**9-Hole Peg Test (s)**					
	Mean (SD)	20.29 (3.42)	21.58 (5.17)	24.93 (10.29)	33.78 (16.52)
	Median (IQR)	19.90 (17.90–22.33)	20.63 (18.35–23.45)	22.79 (19.15–26.89)	28.96 (24.33–36.34)
**PASAT (correct out of max 60)**					
	Mean (SD)	51.92 (9.10)	48.56 (10.64)	46.70 (12.80)	41.84 (14.21)
	Median (IQR)	55 (48–59)	51 (43–57)	51 (39–57)	44 (32–54)
**Volume T2 lesions (mL)**					
	Mean (SD)	4.72 (5.13)	9.70 (10.76)	11.83 (16.29)	16.59 (17.09)
	Median (IQR)	3.00 (1.14–6.33)	6.04 (2.71–12.28)	5.93 (1.96–15.41)	11.45 (4.75–22.68)
**Normalised brain volume (L)**					
	Mean (SD)	1.53 (0.08)	1.54 (0.08)	1.51 (0.10)	1.45 (0.09)
	Median (IQR)	1.53 (1.47–1.58)	1.54 (1.49–1.60)	1.51 (1.44–1.58)	1.44 (1.38–1.51)
**Gd+ T1 lesions**					
	Mean (SD)	0.00 (0.00)	3.76 (7.81)	2.75 (7.40)	0.24 (1.10)
	Median (IQR)	0 (0–0)	2 (1–3)	0 (0–2)	0 (0–0)

Demographic characteristics and MS variables are summarised by clinical disease states across all visits, counting patients each time they were in a specific clinical state; n represents the number of such visits to the specific state.

EDSS, Expanded Disability Status Scale; Gd+, gadolinium-enhancing; IQR, interquartile range; MS, multiple sclerosis; PASAT, Paced Auditory Serial Addition Test; PPMS, primary progressive multiple sclerosis; RRMS, relapsing-remitting multiple sclerosis; SD, standard deviation; SPMS, secondary progressive multiple sclerosis.

Section 4: Individual patient trajectories

Fig. 4.1 Individual patient trajectories of patients diagnosed with RRMS, SPMS or PPMS over a time frame of 5 years

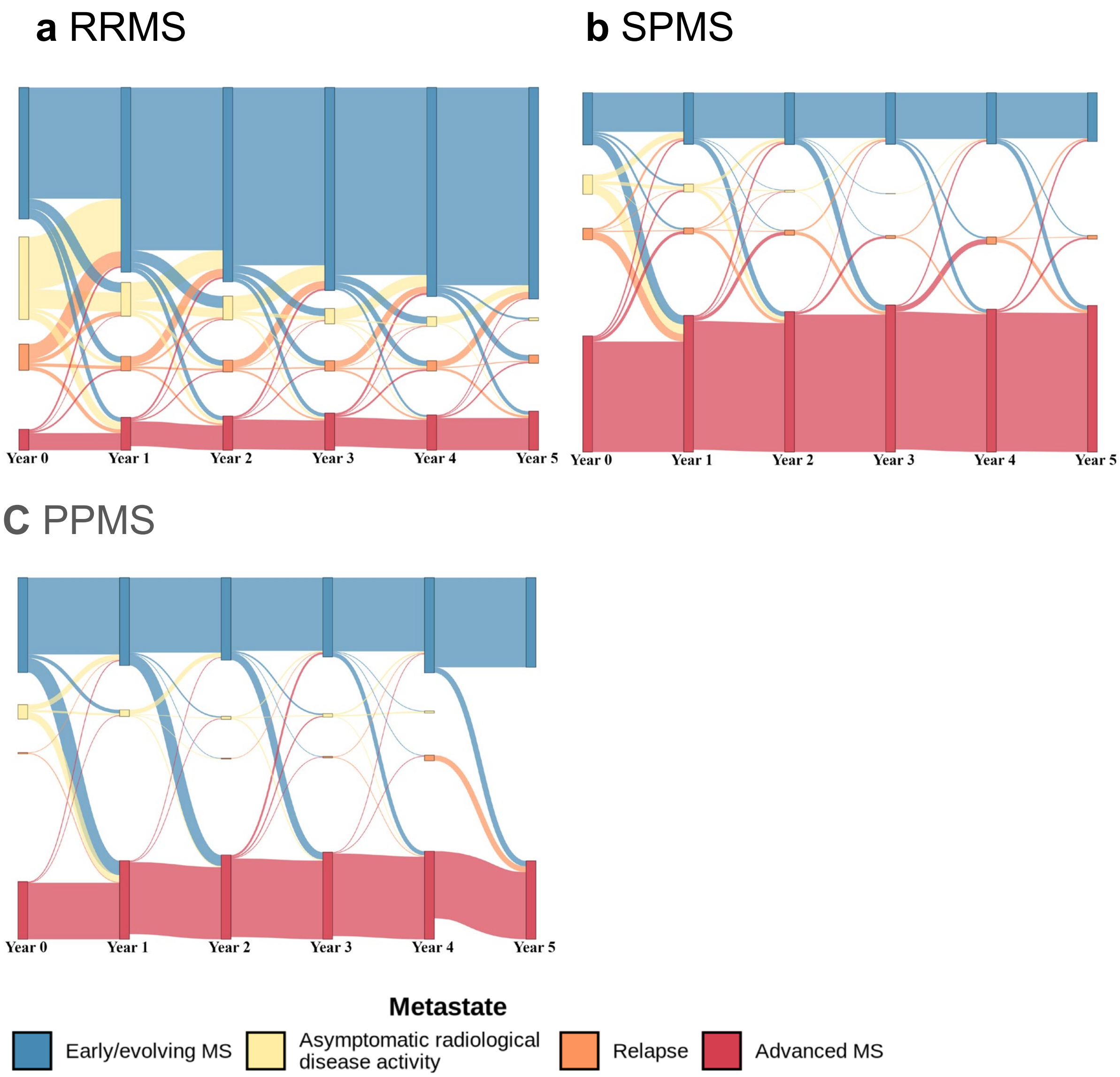

Each plot illustrates (from left to right) the proportion of patients that remain in the same disease state or move to another disease state. Please note that for clarity of the graphic, only the yearly status of the patients is shown and transitions between yearly visits are not displayed to avoid overcrowding the figure. If patients had relapses or radiological inflammation at these annual visits, this is correctly presented in orange and yellow, respectively. However, patients may have experienced relapses or asymptomatic radiological inflammation states between these annual points that contributed to their worsening, which cannot appear in this graphical representation; this explains why the figure displays blue connection lines between EME and advanced disease states in the figure even though the probability of a direct transition between EME and advanced states without passing through the inflammatory states is, in fact, low (the underlying transition matrix is shown in **Fig. 1d**.

Section 5: Methods

5.1 Description of FAHMM

5.1.1 Probabilistic Factor Analysis: MS Dimensions

The motivation here is to combine probabilistic latent factor analysis (PFA) models and hidden Markov models together to address different data modalities and missing data. The PFA exploits the shared information among elements of data at baseline (Y_0) to find MS dimensions (loading matrix) and project the data into a low-dimensional space (composite scores). In this setting, the loading matrix does not change over time. Our PFA mode is

$$Y_0|F_0, \Lambda, \Psi \sim \mathcal{N}(F_0\Lambda', \Psi), \quad (1)$$

$$F_0 \sim \mathcal{N}(0, I_K), \quad (2)$$

where Y_0 is the $N \times P$ vector of observed variables; F_0 is the $N \times M$ vector of latent variables; Λ is the $P \times M$ loading matrix; $\Psi = \text{diag}(\sigma_1^2, \cdots, \sigma_P^2)$ is the residual covariance matrix; I_K is the $K \times K$ diagonal covariance matrix; P is the number of observed variables; M is the number of latent variables ($M < P$); N is the number of subjects. The PFA model in Eq 1 is identifiable only up to an orthogonal transformation of both latent variables (F_0) and loading matrix (Λ). Thus we are using a recently introduced probabilistic factor model (Ročková and George, 2016) where a sparsity constraint is imposed via continuous spike and slab with Laplace components prior on each

element of the loading matrix ($\lambda_{ij} = \Lambda_{ij}$) independently; this achieves model identifiability and greatly improves interpretation of latent variables. The model is

$$\begin{aligned} \lambda_{ij} &\sim (1-\gamma_{ij})\psi(\lambda_{ij}|\omega_{0j}) + \gamma_{ij}\psi(\lambda_{ij}|\omega_1), \\ \gamma_{ij}|\theta_j &\sim \text{Bernoulli}(\theta_j), \quad (3) \\ \theta_k &\sim \mathcal{B}\left(\frac{\alpha}{M}, 1\right) \quad (4) \end{aligned}$$

where $\psi(\lambda|\omega)$ is a Laplace prior with mean zero and variance $2/\omega^2$ where $\omega_{0j} \gg \omega_1 > 0$. Equations 3 and 4 represent a prior on the binary feature allocation matrix $\Gamma = \gamma_{ij}$ where the Indian buffet process (IBP) is adapted (beta-Bernoulli prior). The IBP is a non-parametric Bayesian prior that helps to find the number of latent variables (M) by specifying only an upper bound limit on the number of latent variables. An expectation-maximization algorithm is used to obtain a maximum a posteriori (MAP) estimate of the PFA model parameters using only baseline data where there are no missing data.

To account for missing data post-baseline the observed variable's trajectories over time were modeled using generalized additive models (GAMs) with random effects via the `bam` package in R to impute missing time points. Each variable was modeled marginally using GAM, incorporating follow-up time and subject as random slope and intercept, respectively. Covariates such as age, follow-up time, gender, duration since the first symptom, MS subtypes, number of relapses during the past year before entering the clinical trial, being on active treatment (yes/no), and experiencing relapse (yes/no) were included as fix effects where continuous variables and time were mod-

eled using smoothing splines. The estimated marginal trajectories (fitted models) were used to impute missing time points of all variables except for the number of gadolinium-enhancing lesions where the PFA model is used. Next, the follow-up data are projected to the low-dimensional space using the estimated PFA model as follows:

$$
\begin{aligned}
\mathrm{Cov}(Y_t) = \hat{\Sigma} &= \hat{\Lambda}\hat{\Lambda}' + \hat{\Psi}, \\
Y_t &= \left[Y_{tc}, Y_{tm}\right], && (5) \\
\hat{Y}_{tm} = E\left[Y_{tm}|Y_{tc}, \hat{\Sigma}\right] &= \hat{\Sigma}_{mc}\hat{\Sigma}_c^{-1}Y_c, && (6) \\
F_t^* = E\left[F_t|Y_t, \hat{\Lambda}, \hat{\Psi}\right] &= \left(\hat{\Lambda}'\hat{\Psi}^{-1}\hat{\Lambda} + I_M\right)^{-1}\hat{\Lambda}'\hat{\Psi}^{-1}Y_t', && (7)
\end{aligned}
$$

where Y_{tc} and Y_{tm} are the complete and missing vectors of the observed variables at time point t respectively; F_t^* is a $1 \times M$ latent variable vector, and Σ_{mc} and Σ_c correspond to missing and complete part of the full covariance matrix:

$$
\hat{\Sigma} = \begin{bmatrix} \hat{\Sigma}_m & \hat{\Sigma}_{mc} \\ \hat{\Sigma}_{cm} & \hat{\Sigma}_c \end{bmatrix}
$$

5.1.2 Hidden Markov Model: MS Phenotypes

The estimated composite scores (latent variable vector) are fed into a hidden Markov model (HMM). A continuous-time HMM might be an ideal model choice when the visit times are irregular. However, it can be computationally expensive to fit into large-scale data sets. To address the scalability issue, we discretized continuous time by fitting a discrete-time HMM using

month as a time unit while treating the time points without measurements as missing data and integrating them out. Our proposed method can be written as follows:

$$
\begin{aligned}
F_t^*|z_t = k &\sim \mathcal{N}(\mu_k, \Sigma_k), \\
P(z_t = i|z_{t'} = j) &= (A^{t'-t})_{ij}, \\
P(z_1 = k) &= \pi_k
\end{aligned} \tag{8}
$$

where μ_k is the $1 \times M$ mean vector; Σ_k is the $M \times M$ full covariance matrix; A is a $K \times K$ transition probability matrix; $t' - t$ is the number of time steps between t and t'; and z_t is the latent discrete variable that corresponds to state allocation. The model specification is completed by introducing priors on the rest of the parameters. We use a sparse prior on each row of the transition probability matrix and initial probability matrix to shrink small transitions towards zero to improve the interpretation of model outputs:

$$
\begin{aligned}
A_{i\cdot} &\sim \mathcal{D}(1/K, \cdots, 1/K), \\
\pi &\sim \mathcal{D}(1/K, \cdots, 1/K), \\
\mu_k &\sim \mathcal{N}(b0, B0), \\
\Sigma_k &\sim \mathcal{IW}(c0, C0), \\
C0 &\sim \mathcal{W}(g0, G0),
\end{aligned}
$$

where $A_{i\cdot}$ is the i-th row of the transition probability matrix; π is the initial probability vector; the hyperparameters of emission distribution parameters are set based on Malsiner-Walli et al. (2016) to have weakly informative priors. The expectation-maximization algorithm is used to obtain a MAP estimate of the HMM parameters using the standard forward-backward algorithm (Bishop, 2006).

Figure 5.1 Illustration of (a) the identification of new dimensions to classify MS using probabilistic latent variable modelling and (b) identification of disease states and transition probabilities between these disease states using a multistate model

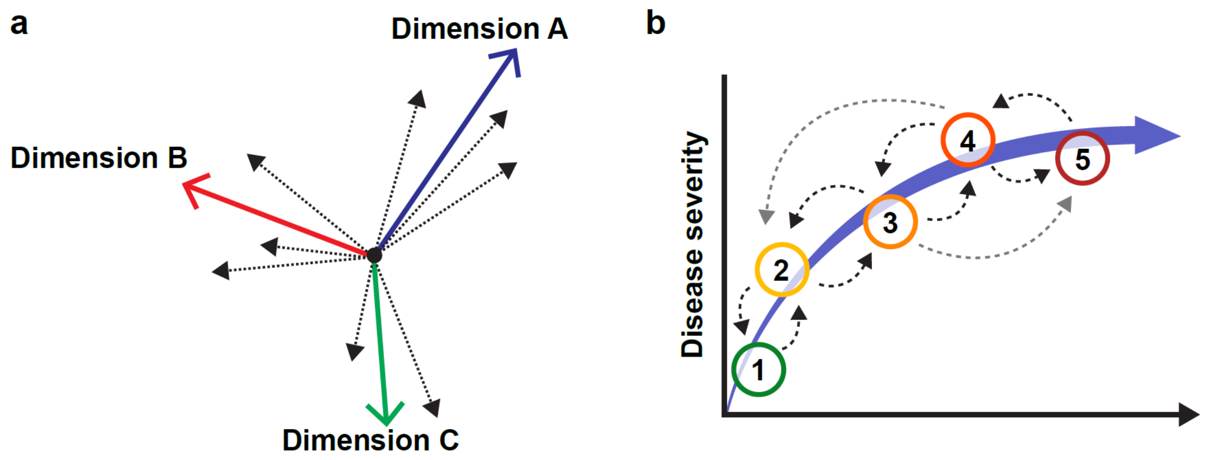

a, The new dimensions (in colour) to classify MS are identified from the original clinical trial variables (in black); they can be calculated from the original measurements and are correlated with those; the composition of these new dimensions is identified in a data-driven way by the model using the spike-and-slab methodology.
b, Identification of disease states and transition probabilities between these disease states based on the longitudinal multimodal patient trajectories using FAHMM models and the new dimensions to classify MS.
FAHMM, factor analysis hidden Markov model; MS, multiple sclerosis.

References

1 Ročková, V. & George, E. I. Fast Bayesian Factor Analysis via Automatic Rotations to Sparsity. *Journal of the American Statistical Association* **111**, 1608-1622 (2016).
2 Malsiner-Walli, G., Frühwirth-Schnatter, S. & Grün, B. Model-based clustering based on sparse finite Gaussian mixtures. *Stat Comput* **26**, 303-324 (2016).
3 Bishop, C. M. & Nasrabadi, N. M. *Pattern recognition and machine learning*. Vol. 4 (Springer, 2006).

5.2 Ethics Committees or Institutional Review Boards

Table 5.2 Independent Ethics Committees or Institutional Review Boards by study

ACROSS (FTY720D 2201)

CANADA

Center 0001

Ottawa Hospital Research Ethics Board
751 Parkdale Ave., Room 106
Ottawa, Ontario
K1Y 1J7
Raphael Saginur, MD REB Chair

Center 0002

The University of British Columbia
Clinical Research Ethics Board
Office of Research Services and Administration
Room 210, Research Pavilion
828 West 10th Ave.
Vancouver, British Columbia
V5Z 1L8
Dr. P. Loewen, REB Chair

Center 0003

St-Michael's Hospital Research Ethics Board
30 Bond St.
Toronto, Ontario
M5B 1W8
C.A. Ottaway, MD, PhD, REB Chair

Center 0004

Montreal Neurological Hospital/ Institute (MNI/H) Research Ethics Board
3801 University Street
Montreal, Quebec
H3A 2B4
Eugene Bereza, MD CSM, CCFP, REB chair

Center 0005

Comité d'éthique de la recherche – Centre Hospitalier de l'Université de Montréal
1560 Sherbrooke Street East
Montreal, Quebec
H2L 4M1
M. André Lavoie, REB Chair

DENMARK

Center 0023

Den Videnskabsetiske Komité for Kobenhavns og Frederiksberg Kommuner
Sundhedsforvaltningen
Sjallandsgaade 40
2200 København N

Center 0025

Den Videnskabsetiske Komité for Københavns Amt
Amtsgaarden
Stationsparken 27
2600 Glostrup

FINLAND

Centers 0029, 0030 and 0031

Helsingin ja Uudenmaan sairaanhoitopiiri
NSK eettinen toimikunta
P.O. Box 220
FIN-00029 HUS
Helsinki

FRANCE

Centers 0037, 0038
Comité consultatif de protection des personnes dans la recherche biomedicale
Assistance Publique Hôpitaux de Marseille
Hôpital SALVATOR
Bâtiment du centre anti-poison
249, boulevard St Marguerite
13274 MARSEILLE CEDEX 9

GERMANY

Center 0043
Universitätsklinikum Würzburg
Ethik-Kommission der Medizinischen Fakultät
Prof. Dr. Graefe
Josef-Schneiderstr. 11
97080 Würzburg

Center 0044
Landesärztekammer BadenWürtemberg
Ethik-Kommission
Jahnstr. 40
70597 Stuttgart

ITALY

Center 0049
Ospedale San Raffaele
Istitutto di ricovero e cura a charattere scientifico
Universita Vita e Salute
Via Olgettina 48
20132-Milano

Center 0050
Comitato Etico Locale
Ospedale S Antonio Abate
Largo A. Boito 2
21013-Gallarate

Center 0051
Comitato Etico DIMI
Ospedale S Martino
Universita degli Studi di Genova
Viale Benedetto XV, 6
16132-Genova

Center 0052
Comitato Etico
Ospedale St Andrea
Universita di Roma "La Sapienza"
Via Grottarossa, 1035-1039
00189-Roma

POLAND

Center 0011
Komisja Bioetyczna
Instytut Psychiatrii i Neurologii
Sobieskiego 9
02-957 Warszawa, Poland

Center 0012
Komisja Bioetyczna
Akademii Medycznej
Zwirki i Wigury 61
02-097 Warszawa, Poland

PORTUGAL

Center 0017
Centro de Estudios Egas Moniz
Faculdade de Medicina de Lisboa
Hospital de Santa Maria
Av. Prof Egas Moniz
1649-028 Lisboa

Center 0018
Conselho de Administraçao
Hospital S Antonio dos Capuchos
Servicio de Neurologia
Alameda S Antonio dos Capuchos
Lisboa, 1169-050

Center 0019

Comissão de Ética para a Saúde
Hospitais da Universidade de Coimbra
Departamento de Educação e Investigação
Gabinete de Apoio à Investigação
Av. Bissaya Barreto e Praceta Prof. Mota Pinto
3000-075 Coimbra

SPAIN

Center 0061

Hospital Universitari Vall D'Hebron
Comite etico de investigation clinica
Dirección de Investigación, 2ª planta
Edificio de Laboratorios de Investigación
Paseo Valle de Hebron 119
08035 Barcelona

Center 0062

Hospital universitario de Bellvitge
Dr. Pau Ferrer Salvans
Comité ético de investigation clinica
C/ Feixa Llarga, s/n
08907 L'Hospitalet de Llobregat
Barcelona

Centers 0063, 0064

Comité autonomico de ensayos clinicos de Andalucia (CAEC)
Att. Dra. Isabel Fernández Fernández
Consejería de Salud
Avda. Innovación, s/n. Edif.. Arena 1
41020 - Sevilla

Center 0065

Comite etico de investigation clinica
Att. Dr Alfonso Moreno González
Hospital Clinico San Carlos
C. Dr Martin Lagos
28040 Madrid

Center 0066

Comite etico de investigation clinica
Att. Dr. Miguel Gobernado Serrano
Hospital Universitario de Fe Valencia
Av Campanar 21
46009 Valencia

SWITZERLAND

Center 0071

Ethikkomission beider Basel
Hebelstrasse 53
4056 Basel

Center 0072

Kantonale Ethikkomission des Kantons Zuerich
SPUK für Neurologie
Klinik Schlössli
8618 Oetwil am See

UK

Center 0074

Len Key
Newcastle and North Tyneside Local Research Ethics Committee
Room G14
The Dental School
Framlington Place
Newcastle upon Tyne
NE2 4BW

ASCLEPIOS I (COMB157G2301)

Independent Ethics Committees or Institutional Review Boards by study center

Center No.	Ethics Committee or Institutional Review Board	Department / Organization	Address
6021	Comité Independiente de Ética Para Ensayos En Farmacologia Clinica	Fundación De Estudios Farmacologia y de Medicamentos FEFyM	Buenos Aires C1027AAP Argentina
6023	Comité de Ética en Investigacion Biomédica Fundación Rosarina de Neurorehabilitación		Rosario Santa Fe S2000BZL Argentina
6025	Comité Institucional de Etica de la Investigacion en Salud del Adulto - CIEIS		Cordoba Cordoba X5004CDT Argentina
1000	Austin Health HREC		Heidelberg VIC 3084 Australia
1001	Austin Health HREC		Heidelberg VIC 3084 Australia
1021	Ethische Commissie Onderzoek UZ/KU Leuven		Leuven 3000 Belgium
1022	Ethische Commissie Onderzoek UZ/KU Leuven		Leuven 3000 Belgium
1023	Ethische Commissie Onderzoek UZ/KU Leuven		Leuven 3000 Belgium
1025	Ethische Commissie Onderzoek UZ/KU Leuven		Leuven 3000 Belgium
1040	Ethics Committee for Multicentre Trials		Sofia 1000 Bulgaria
1041	Ethics Committee for Multicentre Trials		Sofia 1000 Bulgaria
1042	Ethics Committee for Multicentre Trials		Sofia 1000 Bulgaria
1061	MUCH Research Ethics Board		Montreal Quebec H3A 2B4 Canada

Center No.	Ethics Committee or Institutional Review Board	Department / Organization	Address
1062	Institutional Review Board (IRB) Services		Aurora<br>ON L4G 0A5<br>Canada
1063	University of British Columbia	Clinical Research Ethics Board	Vancouver<br>British Columbia V5Z 1L8<br>Canada
1064	MUHC REB Neupsy	Research Ethics Board	Montreal<br>Quebec H3A 2B4<br>Canada
1081	Središnje etičko povjerenstvo	Agencija za lijekove i medicinske proizvode	Zagreb 10000<br>Croatia
1082	Središnje etičko povjerenstvo	Agencija za lijekove i medicinske proizvode	Zagreb 10000<br>Croatia
2000	Eticka komise, Pardubicka krajska nemocnice a.s.	Kyjevska 44	Pardubice 532 03<br>Czech Republic
2001	Fakultní nemocnice v Motole	Eticka komise pro multicentricka hodnoceni	Praha 5 150 06<br>Czech Republic
2002	Multicentric Ethics Committee IKEM and TN		Prague 4 140 59<br>Czech Republic
2003	Eticka komise, Fakultni nemocnice Hradec Kralove		Sokolsak 581<br>Hradec Kralove 500 05<br>Czech Republic
2004	Eticka komise, Nemocnice Jihlava		Vrchlickeho 59<br>Jihlava 586 33<br>Czech Republic
2005	Eticka komise FN a LF UP v Olomouci		I.P. Pavlova 6 O<br>lomouc 775 20<br>Czech Republic
6060	De Videnskabsetiske Komiteer for Region Syddanmark	Regionshuset, Damhaven 12	Vejle 7100<br>Denmark
6061	De Videnskabsetiske Komiteer for Region Syddanmark	Regionshuset, Damhaven 12	Vejle 7100<br>Denmark
6063	De Videnskabsetiske Komiteer for Region Syddanmark	Regionshuset, Damhaven 12	Vejle 7100<br>Denmark
6080	Tallinn Medical Research Ethics Committee		Tallinn 11619<br>Estonia
6081	Tallinn Medical Research Ethics Committee		Tallinn 11619<br>Estonia
2020	CPP OUEST IV	CHU de Nantes -<br>5 allée de l'ile gloriette	Nantes Cedex 1 44093<br>France
2023	CPP OUEST IV	CHU de Nantes -<br>5 allée de l'ile gloriette	Nantes Cedex 1 44093<br>France

Center No.	Ethics Committee or Institutional Review Board	Department / Organization	Address
2024	CPP OUEST IV	CHU de Nantes - 5 allée de l'ile gloriette	Nantes Cedex 1 44093 France
2025	CPP OUEST IV	CHU de Nantes - 5 allée de l'ile gloriette	Nantes Cedex 1 44093 France
2051	Ethikkommission der Ärztekammer Westfalen-Lippe und der Medizinischen Fakultät der Westfälischen Wilhelms-Universität Münster		Münster 48147 Germany
2052	Ethikkommission der Ärztekammer Westfalen-Lippe und der Medizinischen Fakultät der Westfälischen Wilhelms-Universität Münster		Münster 48147 Germany
2053	Ethikkommission der Ärztekammer Westfalen-Lippe und der Medizinischen Fakultät der Westfälischen Wilhelms-Universität Münster		Münster 48147 Germany
2054	Ethikkommission der Ärztekammer Westfalen-Lippe und der Medizinischen Fakultät der Westfälischen Wilhelms-Universität Münster		Münster 48147 Germany
2055	Ethikkommission der Ärztekammer Westfalen-Lippe und der Medizinischen Fakultät der Westfälischen Wilhelms-Universität Münster		Münster 48147 Germany
2060	Ethikkommission der Ärztekammer Westfalen-Lippe und der Medizinischen Fakultät der Westfälischen Wilhelms-Universität Münster		Münster 48147 Germany
2063	Ethikkommission der Ärztekammer Westfalen-Lippe und der Medizinischen Fakultät der Westfälischen Wilhelms-Universität Münster		Münster 48147 Germany
2065	Ethikkommission der Ärztekammer Westfalen-Lippe und der Medizinischen Fakultät der Westfälischen Wilhelms-Universität Münster		Münster 48147 Germany
2067	Ethikkommission der Ärztekammer Westfalen-Lippe und der Medizinischen Fakultät der Westfälischen Wilhelms-Universität Münster		Münster 48147 Germany

Center No.	Ethics Committee or Institutional Review Board	Department / Organization	Address
2069	Ethikkommission der Ärztekammer Westfalen-Lippe und der Medizinischen Fakultät der Westfälischen Wilhelms-Universität Münster		Münster 48147<br>Germany
2073	Ethikkommission der Ärztekammer Westfalen-Lippe und der Medizinischen Fakultät der Westfälischen Wilhelms-Universität Münster		Münster 48147<br>Germany
7000	Local Ethics Committee of 401 General Military Hospital of Athens		Athens 11525<br>Greece
7001	Local Ethics Committee of Aeginition University General Hospital of Athens		Athens 11528<br>Greece
7004	Local Ethics Committee of AHEPA University General Hospital of Thessaloniki		Thessaloniki 54636<br>Greece
3000	Medical Research Council, Ethics Committee for Clinical Pharmacology		Budapest 1051<br>Hungary
3001	Medical Research Council, Ethics Committee for Clinical Pharmacology		Budapest 1051<br>Hungary
3002	Medical Research Council, Ethics Committee for Clinical Pharmacology		Budapest 1051<br>Hungary
3003	Medical Research Council, Ethics Committee for Clinical Pharmacology		Budapest 1051<br>Hungary
3051	Institutional Ethics Committee	AMRITA Institute of medical sciences and research center	Kochi<br>Kerala 682041<br>India
3052	Ethical Review Board	MS Ramaiah Medical College &Teaching Hospital	Bangalore<br>Karnataka 560054<br>India
3053	Institutional Ethics Committee, PGIMER	PGIMER	Chandigarh<br>Punjab 160012<br>India
3054	Max Healthcare Institute Limited		Saket New Delhi<br>Delhi 110017<br>India
3057	The Ethics Committee of Sri Aurobindo Seva Kendra	Sri Aurobindo Seva Kendra	Kolkata<br>West Bengal 700068<br>India
3058	Central Ethics Committee	Justice K s Hegde Hospital, Deralakatte	Mangalore<br>Karnataka 575018<br>India
7020	Sourasky MC Helsinki		Tel Aviv 64239<br>Israel

Center No.	Ethics Committee or Institutional Review Board	Department / Organization	Address
7021	Barzilai MC Helsinki		Ashkelon 78278<br>Israel
7022	Ziv MC Helsinki		Safed 13100<br>Israel
7023	Rambam MC Helsinki		Haifa 31096<br>Israel
3020	Comitato Etico Dell'irccs Ospedale San Raffaele Di		Milano 20132<br>Italy
3021	Comitato Etico Seconda Universita' Degli Studi Di		Naples 88138<br>Italy
3022	Comitato Etico Dell'insubria C/O Ospedale Di Circolo		Varese 21100<br>Italy
3023	Comitato Etico Universita' Federico Ii Di Napoli		Naples 80131<br>Italy
3029	Comitato Etico Dell'universita' Sapienza		Roma 00161<br>Italy
3083	Sanatorio Alcocer Pozo		Queretaro<br>Queretaro 76000<br>Mexico
7060	VU medisch centrum	METc	Amsterdam 1081 BT<br>Netherlands
7061	Zuyderland Medisch Centrum	Raad van Bestuur	Heerlen 6419 PC<br>Netherlands
7063	Erasmus MC	Raad van Bestuur	Rotterdam 6525 GA<br>Netherlands
4000	Komisja Bioetyczna przy Uniwersytecie Medycznym w Lodzi	Pl. Hallera 1B	Lodz Poland 90-647<br>Poland
4001	Komisja Bioetyczna przy Uniwersytecie Medycznym w Lodzi	Pl. Hallera 1B	Lodz Poland 90-647<br>Poland
4002	Komisja Bioetyczna przy Uniwersytecie Medycznym w Lodzi	Pl. Hallera 1B	Lodz Poland 90-647<br>Poland
4003	Komisja Bioetyczna przy Uniwersytecie Medycznym w Lodzi	Pl. Hallera 1B	Lodz Poland 90-647<br>Poland
4004	Komisja Bioetyczna przy Uniwersytecie Medycznym w Lodzi	Pl. Hallera 1B	Lodz Poland 90-647<br>Poland
4005	Komisja Bioetyczna przy Uniwersytecie Medycznym w Lodzi	Pl. Hallera 1B	Lodz Poland 90-647<br>Poland
4006	Komisja Bioetyczna przy Uniwersytecie Medycznym w Lodzi	Pl. Hallera 1B	Lodz Poland 90-647<br>Poland

Center No.	Ethics Committee or Institutional Review Board	Department / Organization	Address
4040	LEC Kemerovo Regional Clinical Hospital		Kemerovo 650066<br>Russia
4041	Local Ethic Committee of Siberian District Medical Centre of Federal Medical-Biological Agency of Russia	Siberian District Medical Centre of Federal Medical-Biological Agency of Russia	Novosibirsk 630007<br>Russia
4042	Military Medical Academy n.a. S.M.Kirov	LEC	Saint-Petersburg 194044<br>Russia
4043	LEC Independent Interdisciplinary Ethics Committee		Moscow 125468<br>Russia
4044	LEC Privolzhskyi Federal Medical Research Center		Nizhny Novgorod 603155<br>Russia
4046	LEC Republican Clinical Hospital 4		Saransk 430032<br>Russia
4047	LEC Research Center of Neurology		Moscow 125367<br>Russia
4048	Municipal hospital 40 of the Kurortnyi Region		Sestroretsk 197706<br>Russia
4060	Eticka komisia UN Bratislava	Nemocnica Ruzinov, Pazitkova 4	Bratislava 821 01<br>Slovakia (Slovak Republic)
4061	EK UNB Nemocnica akademika L.Dérera	Limbova 5	Bratislava Slovakia 83101 Slovakia (Slovak Republic)
4062	Eticka komisia FN Trnava	A.Zarnova 11	Trnava 917 75<br>Slovakia (Slovak Republic)
4063	Etická komisia pri FN Nitra	Špitálska 6	Nitra 950 01<br>Slovakia (Slovak Republic)
4064	EK pri FNsP F.D.Roosevelta	Nám. L.Svobodu 1	Banska Bystrica 97517<br>Slovakia (Slovak Republic)
4081	Comité de Ética de Virgen Macarena y Virgen del Rocio		Sevilla<br>Andalucia 41013<br>Spain
4083	Comité de Ética de Virgen Macarena y Virgen del Rocio		Sevilla<br>Andalucia 41013<br>Spain
4085	Comité de Ética de Virgen Macarena y Virgen del Rocio		Sevilla<br>Andalucia 41013<br>Spain
4086	Comité de Ética de Virgen Macarena y Virgen del Rocio		Sevilla<br>Andalucia 41013<br>Spain
4087	Comité de Ética de Virgen Macarena y Virgen del Rocio		Sevilla<br>Andalucia 41013<br>Spain

Center No.	Ethics Committee or Institutional Review Board	Department / Organization	Address
4088	Comité de Ética de Virgen Macarena y Virgen del Rocio		Sevilla<br>Andalucia 41013<br>Spain
4089	Comité de Ética de Virgen Macarena y Virgen del Rocio		Sevilla<br>Andalucia 41013<br>Spain
4090	Comité de Ética de Virgen Macarena y Virgen del Rocio		Sevilla<br>Andalucia 41013<br>Spain
4091	Comité de Ética de Virgen Macarena y Virgen del Rocio		Sevilla<br>Andalucia 41013<br>Spain
8000	Regionala etikprövningsnämnden i Stockholm	FE289	Stockholm 171 77<br>Sweden
8001	Regionala etikprövningsnämnden i Stockholm	FE289	Stockholm 171 77<br>Sweden
6000	Ethikkommission Nordwest- und Zentralschweiz (EKNZ)		Basel 4056<br>Switzerland
8040	The Khon Kaen University Ethics Committee for Human Research		Khon Kaen 40002<br>Thailand
8060	Kocaeli University Clinical Research Ethics Committee		Kocaeli 41380<br>Turkey
8061	Kocaeli University Clinical Research Ethics Committee		Kocaeli 41380<br>Turkey
8062	Kocaeli University Clinical Research Ethics Committee		Kocaeli 41380<br>Turkey
8064	Kocaeli University Clinical Research Ethics Committee		Kocaeli 41380<br>Turkey
8081	NRES Committee South Central - Southampton B	Bristol REC Centre, Level 3 Block B	Bristol BS1 2NT<br>United Kingdom
8083	NRES Committee South Central - Southampton B	Bristol REC Centre, Level 3 Block B	Bristol BS1 2NT<br>United Kingdom
8084	NRES Committee South Central - Southampton B	Bristol REC Centre, Level 3 Block B	Bristol BS1 2NT<br>United Kingdom
5001	Schulman Associates, IRB		Cincinnati<br>OH 45242<br>United States
5002	Schulman Associates, IRB		Cincinnati<br>OH 45242<br>United States
5003	Schulman Associates, IRB		Cincinnati<br>OH 45242<br>United States
5004	Schulman Associates, IRB		Cincinnati<br>OH 45242<br>United States

Center No.	Ethics Committee or Institutional Review Board	Department / Organization	Address
5005	Schulman Associates, IRB		Cincinnati<br>OH 45242<br>United States
5006	Schulman Associates, IRB		Cincinnati<br>OH 45242<br>United States
5007	Schulman Associates, IRB		Cincinnati<br>OH 45242<br>United States
5008	Schulman Associates, IRB		Cincinnati<br>OH 45242<br>United States
5010	Schulman Associates, IRB		Cincinnati<br>OH 45242<br>United States
5015	Washington University	Human Research Protection Office	St. Louis<br>MO 63110<br>United States
5016	IRB University of California Davis		Sacramento<br>CA 95817<br>United States
5017	Schulman Associates, IRB		Cincinnati<br>OH 45242<br>United States
5021	Schulman Associates, IRB		Cincinnati<br>OH 45242<br>United States
5022	University of Kansas Medical Center	Human Subjects Committee	Kansas City<br>KS 66160<br>United States
5023	WakeMed IRB		Raleigh<br>NC 27705<br>United States
5025	Schulman Associates, IRB		Cincinnati<br>OH 45242<br>United States
5026	Schulman Associates, IRB		Cincinnati<br>OH 45242<br>United States
5027	Cleveland Clinic Institutional Review Board		Cleveland<br>OH 44195<br>United States
5028	Schulman Associates, IRB		Cincinnati<br>OH 45242<br>United States
5029	Schulman Associates, IRB		Cincinnati<br>OH 45242<br>United States
5033	ADVARRA, Inc.		Columbia<br>Maryland 21046<br>United States

Center No.	Ethics Committee or Institutional Review Board	Department / Organization	Address
5034	Schulman Associates, IRB		Cincinnati<br>OH 45242<br>United States
5035	Schulman Associates, IRB		Cincinnati<br>OH 45242<br>United States
5037	Schulman Associates, IRB		Cincinnati<br>OH 45242<br>United States
5038	Schulman Associates, IRB		Cincinnati<br>OH 45242<br>United States
5039	Schulman Associates, IRB		Cincinnati<br>OH 45242<br>United States
5041	Schulman Associates, IRB		Cincinnati<br>OH 45242<br>United States
5042	St Joseph's Hospital and Medical Center	IRB Review Board Panel B	Phoenix<br>Arizona 85013<br>United States
5044	Georgetown University IRB		Washington<br>DC 20007<br>United States
5045	Sutter Health Institutional Review Board		Walnut Creek<br>CA 94596<br>United States
5046	Schulman Associates, IRB		Cincinnati<br>OH 45242<br>United States
5048	Rush University Medical Center		Chicago<br>IL 60612<br>United States
5049	Indiana University	IRB Office of the VP for Research Office of Research Compliance	Bloomington<br>IN 46202<br>United States
5050	Crescent City IRB		New Orleans LA 70119<br>United States
5052	Oklahoma Medical Research Foundation	IRB Office	Oklahoma City<br>Oklahoma 73104-5046<br>United States
5056	Quorum Review IRB		Seattle<br>WA 98101<br>United States
5057	Schulman Associates, IRB		Cincinnati<br>OH 45242<br>United States
5058	WIRB		Olympia<br>WA 98502-5010<br>United States

Center No.	Ethics Committee or Institutional Review Board	Department / Organization	Address
5061	Schulman Associates, IRB		Cincinnati<br>OH 45242<br>United States
5064	Wheaton Franciscan Healthcare IRB		Glendale<br>WI 53212<br>United States
5067	Schulman Associates, IRB		Cincinnati<br>OH 45242<br>United States
5068	Schulman Associates, IRB		Cincinnati<br>OH 45242<br>United States
5071	Cleveland Clinic Institutional Review Board		Cleveland<br>OH 44195<br>United States
5072	ADVARRA, Inc.		Columbia<br>Maryland 21046<br>United States
5076	Schulman Associates, IRB		Cincinnati<br>OH 45242<br>United States
5077	University of Texas Health Science Center San Antonio	Institutional Review Board	San Antonio<br>TX 78229<br>United States
5078	Schulman Associates, IRB		Cincinnati<br>OH 45242<br>United States
5079	Mercy Medical Center Des Moines		Des Moines<br>Iowa 50314<br>United States
5080	Schulman Associates, IRB		Cincinnati<br>OH 45242<br>United States
5081	Schulman Associates, IRB		Cincinnati<br>OH 45242<br>United States
5084	Schulman Associates, IRB		Cincinnati<br>OH 45242<br>United States
5086	Schulman Associates, IRB		Cincinnati<br>OH 45242<br>United States
5088	Schulman Associates, IRB		Cincinnati<br>OH 45242<br>United States
5089	Schulman Associates, IRB		Cincinnati<br>OH 45242<br>United States
5090	Wayne State University IRB		Detroit<br>MI 48201<br>United States

Center No.	Ethics Committee or Institutional Review Board	Department / Organization	Address
5091	Schulman Associates, IRB		Cincinnati<br>OH 45242<br>United States
5093	Schulman Associates, IRB		Cincinnati<br>OH 45242<br>United States
5094	Schulman Associates, IRB		Cincinnati<br>OH 45242<br>United States
5095	Schulman Associates, IRB		Cincinnati<br>OH 45242<br>United States
5098	Schulman Associates, IRB		Cincinnati<br>OH 45242<br>United States

ASCLEPIOS II (COMB157G2302)

Independent Ethics Committees (IECs) or Institutional Review Boards (IRBs) by study center

Center No.	Ethics Committee or Institutional Review Board	Department / Organization	Address
8062	CEIB - Comité de Etica en Investigaciones Biomedicas	FLENI	Buenos Aires<br>C1428AQK<br>Argentina
8064	Comité Independiente de Ética Para Ensayos En Farmacologia Clinica	Fundación De Estudios Farmacologia y de Medicamentos FEFyM	Buenos Aires<br>C1027AAP<br>Argentina
8065	Comite de Bioetica del Instituto de Investigaciones Clinicas Rosario - COBIIC		Rosario Santa Fe<br>S2000CVD<br>Argentina
1022	Austin Health HREC		Heidelberg<br>VIC 3084<br>Australia
6022	Ethikkommission Medizinische Universität Wien		Wien A-1090<br>Austria
6023	Ethikkommission Medizinische Universität Wien		Wien A-1090<br>Austria
1040	UCL Saint Luc Comité d'Ethique Hospitalo-Facultaire		Brussels 1200<br>Belgium
1041	UCL Saint Luc Comité d'Ethique Hospitalo-Facultaire		Brussels 1200<br>Belgium
1042	UCL Saint Luc Comité d'Ethique Hospitalo-Facultaire		Brussels 1200<br>Belgium
1045	UCL Saint Luc Comité d'Ethique Hospitalo-Facultaire		Brussels 1200<br>Belgium
1060	Ethics Committee for Multicentre Trials		Sofia 1000<br>Bulgaria
1062	Ethics Committee for Multicentre Trials		Sofia 1000<br>Bulgaria
1070	Ottawa Health Science Network	Research Ethics Board	Ottawa<br>Ontario K1Y 4E9<br>Canada
1071	IRB Services		Aurora<br>Ontario L4G 0A5<br>Canada
1073	Sunnybrook	Research Ethics Board	Toronto<br>Ontario M4N 3M5<br>Canada
1075	Western University Health Science	Research Ethics Board	London<br>Ontario N6G 1G9<br>Canada
1090	Središnje etičko povjerenstvo	Agencija za lijekove i medicinske proizvode	Zagreb 10000<br>Croatia
1091	Središnje etičko povjerenstvo	Agencija za lijekove i medicinske proizvode	Zagreb 10000<br>Croatia

Center No.	Ethics Committee or Institutional Review Board	Department / Organization	Address
1092	Središnje etičko povjerenstvo	Agencija za lijekove i medicinske proizvode	Zagreb 10000<br>Croatia
2000	Eticka komise,<br>FN u sv. Anny v Brne		Pekarska 53<br>Brno 656 91<br>Czech Republic
2001	Eticka komise,<br>Fakultni nemocnice Kralovske Vinohrady		Srobarova 50<br>Praha 10 100 34<br>Czech Republic
2002	Eticka komise,<br>Fakultni Nemocnice Kralovske Vinohrady		Srobarova 50<br>Praha 10 100 34<br>Czech Republic
2003	Eticka komise<br>Vseobecne Fakultni Nemocnice v Praze		Na Bojisti 1<br>Praha 2 128 08<br>Czech Republic
2004	Eticka komise,<br>Krajska zdravotni a.s. - Nemocnice Teplice o.z.		Duchcovska 53<br>Teplice 415 29<br>Czech Republic
7000	VSSHP Eettinen toimikunta		Turku 20520<br>Finland
7001	VSSHP Eettinen toimikunta		Turku 20520<br>Finland
2030	CPP Sud Mediterranée IV	Hôpital Saint Eloi	Montpellier 34295 Cedex 5<br>France
2031	CPP Sud Mediterranée IV	Hôpital Saint Eloi	Montpellier 34295 Cedex 5<br>France
2032	CPP Sud Mediterranée IV	Hôpital Saint Eloi	Montpellier 34295 Cedex 5<br>France
2033	CPP Sud Mediterranée IV	Hôpital Saint Eloi	Montpellier 34295 Cedex 5<br>France
2035	CPP Sud Mediterranée IV	Hôpital Saint Eloi	Montpellier 34295 Cedex 5<br>France
2036	CPP Sud Mediterranée IV	Hôpital Saint Eloi	Montpellier 34295 Cedex 5<br>France
2037	CPP Sud Mediterranée IV	Hôpital Saint Eloi	Montpellier 34295 Cedex 5<br>France
9000	Technische Universität Dresden	Medizinische Fakultät Carl Gustav Carus / Ethik-Kommission	Dresden 01307<br>Germany
9002	Technische Universität Dresden	Medizinische Fakultät Carl Gustav Carus / Ethik-Kommission	Dresden 01307<br>Germany
9003	Technische Universität Dresden	Medizinische Fakultät Carl Gustav Carus / Ethik-Kommission	Dresden 01307<br>Germany
9005	Technische Universität Dresden	Medizinische Fakultät Carl Gustav Carus / Ethik-Kommission	Dresden 01307<br>Germany
9006	Technische Universität Dresden	Medizinische Fakultät Carl Gustav Carus / Ethik-Kommission	Dresden 01307<br>Germany

Center No.	Ethics Committee or Institutional Review Board	Department / Organization	Address
9008	Technische Universität Dresden	Medizinische Fakultät Carl Gustav Carus / Ethik-Kommission	Dresden 01307<br>Germany
9011	Technische Universität Dresden	Medizinische Fakultät Carl Gustav Carus / Ethik-Kommission	Dresden 01307<br>Germany
9012	Technische Universität Dresden	Medizinische Fakultät Carl Gustav Carus / Ethik-Kommission	Dresden 01307<br>Germany
9015	Technische Universität Dresden	Medizinische Fakultät Carl Gustav Carus / Ethik-Kommission	Dresden 01307<br>Germany
9018	Technische Universität Dresden	Medizinische Fakultät Carl Gustav Carus / Ethik-Kommission	Dresden 01307<br>Germany
9019	Technische Universität Dresden	Medizinische Fakultät Carl Gustav Carus / Ethik-Kommission	Dresden 01307<br>Germany
9020	Technische Universität Dresden	Medizinische Fakultät Carl Gustav Carus / Ethik-Kommission	Dresden 01307<br>Germany
9021	Technische Universität Dresden	Medizinische Fakultät Carl Gustav Carus / Ethik-Kommission	Dresden 01307<br>Germany
9022	Technische Universität Dresden	Medizinische Fakultät Carl Gustav Carus / Ethik-Kommission	Dresden 01307<br>Germany
3000	Medical Research Council	Ethics Committee for Clinical Pharmacology (ECCP)	Budapest 1054<br>Hungary
3001	Medical Research Council	Ethics Committee for Clinical Pharmacology (ECCP)	Budapest 1054<br>Hungary
2050	Institutional Ethics Committee, P.D. Hinduja Hospital and Medical Research Center, Veer Savarkar Marg, Mahim	Neurology	Mumbai Maharashtra 400016<br>India
2051	Ethics Committee for Research on Human Subjects	Seth G.S Medical College & KEM Hospital, Parel	Mumbai Maharashtra 400012<br>India
2052	NIMS Institutional Ethics Committee	Nizam's Institute of Medical Sciences	Hyderabad Telangana 500018<br>India
2053	Institutional Ethics committee, Deenanath Mangeshkar Hospital and Research Center, Erandwane, Pune	Neurology	Pune Maharashtra 411004<br>India
2054	Institutional Ethics committee, Topiwala National medical college & BYL Nair, CHS. Hospital, A. L, Niar Road, Mumbai Central	Neurology	Mumbai Maharashtra 400008<br>India
2055	Ethics Committee	Sir Ganga Ram Hospital	New Delhi<br>Delhi 110060<br>India

Center No.	Ethics Committee or Institutional Review Board	Department / Organization	Address
2057	Institutional Ethics Committee	Christian Medical College & Hospital	Ludhiana<br>Punjab 141008<br>India
2070	Comitato Etico Regionale Della Liguria C/O Irccs Aou San		Genova 16132<br>Italy
2072	Comitato Etico Seconda Universita' Degli Studi Di Napoli - Aou Sun-Aorn Ospedale Dei Colli		Napoli 80131<br>Italy
2073	Comitato Etico Indipendente Presso La Fondazione Ptv Policlinico Tor Vergata Di Roma		Roma 00166<br>Italy
2076	Comitato Etico Regione Lombardia	Carlo Besta	Milano 20133<br>Italy
2077	Ce Sper.Comitato Etico Per La Sperimentazione Clinica Della Provincia Di Padova Presso Aou	AOU Padova	Padova 35128<br>Italy
2078	Comitato Etico Lazio 2	ASL Roma2	Roma 00142<br>Italy
7020	Ethics Committee For Clinical Research At Pauls Stradins Clinical University Hospital		Riga LV-1002<br>Latvia
7021	Ethics Committee For Clinical Research At Pauls Stradins Clinical University Hospital		Riga LV-1002<br>Latvia
7022	Ethics Committee For Clinical Research At Pauls Stradins Clinical University Hospital		Riga LV-1002<br>Latvia
7041	Lithuanian Bioethics Committee	Central EC for Lithuania	Vilnius N/A LT-03219<br>Lithuania
7042	Lithuanian Bioethics Committee	Central EC for Lithuania	Vilnius LT-03219<br>Lithuania
3050	Hospital Angeles Chihuahua	IRB	Chihuahua<br>Chihuahua 31238<br>Mexico
7060	REK	Vest	Bergen 5020 Norway
3070	Comite de Etica en Investigacion Biomedica del Hospital Nacional Dos de Mayo		Cercado de Lima<br>Lima 15003<br>Peru
3071	Hospital Nacional Guillermo Almenara Irigoyen		La Victoria Lima 15033<br>Peru
3072	Comite Institucional de Etica en Investigacion de la Clinica Anglo Americana		San isidro Lima 15073<br>Peru
4010	Komisja Bioetyczna przy Okregowej izbie Lekarskiej	ul. Dekerta 2	Rzeszow Poland 35-030<br>Poland
4011	Komisja Bioetyczna przy Okregowej izbie Lekarskiej	ul. Dekerta 2	Rzeszow Poland 35-030<br>Poland
4012	Komisja Bioetyczna przy Okregowej izbie Lekarskiej	ul. Dekerta 2	Rzeszow Poland 35-030<br>Poland
4013	Komisja Bioetyczna przy Okregowej izbie Lekarskiej	ul. Dekerta 2	Rzeszow Poland 35-030<br>Poland

Center No.	Ethics Committee or Institutional Review Board	Department / Organization	Address
4017	Komisja Bioetyczna przy Okregowej izbie Lekarskiej	ul. Dekerta 2	Rzeszow Poland 35-030 Poland
4040	Comissão de Ética para a Investigação Clínica		Lisboa 1749-004 Portugal
4041	Comissão de Ética para a Investigação Clínica		Lisboa 1749-004 Portugal
4042	Comissão de Ética para a Investigação Clínica		Lisboa 1749-004 Portugal
4043	Comissão de Ética para a Investigação Clínica		Lisboa 1749-004 Portugal
4045	Comissão de Ética para a Investigação Clínica		Lisboa 1749-004 Portugal
4046	Comissão de Ética para a Investigação Clínica		Lisboa 1749-004 Portugal
4047	Comissão de Ética para a Investigação Clínica		Lisboa 1749-004 Portugal
4048	Comissão de Ética para a Investigação Clínica		Lisboa 1749-004 Portugal
4049	Comissão de Ética para a Investigação Clínica		Lisboa 1749-004 Portugal
4050	Comissão de Ética para a Investigação Clínica		Lisboa 1749-004 Portugal
8080	Local Committee of City Clinical Hospital #31		Saint-Petersburg 197110 Russia
8081	Local Committee of Institute of the Human Brain of the Russian Academy of Sciences		Saint-Petersburg 197376 Russia
8082	Local Ethics Committee at Republican Clinical Hospital of Rehabilitation Treatment of Ministry of Health of Tatarstan Republic		Kazan 420021 Russia
8083	LEC LLC Medis		Nizhny Novgorod 603137 Russia
8085	LEC Sverdlovsk Region Clinical Hospital#1		Ekaterinburg 620102 Russia
8086	LEC State Novosibirsk Regional Clinical Hospital		Novosibirsk 630087 Russia
8087	LEC Siberian Clinical Centre of FMBA		Krasnoyarsk 660037 Russia
8088	City Clinical Hospital #24	LEC	Moscow 127015 Russia
8090	LEC of State Pavlov Medical University		Saint-Petersburg 197101 Russia
4060	Eticka Komisia Lekarskej Fakulty Univerzity Komenskeho A Univerzitnej Nemocnica Bratislava	Nemocnica Stare Mesto	Bratislava 85107 Slovakia (Slovak Republic)
4061	Etická komisia Univerzitnej nemocnice Martin	Kollárova 2	Martin Slovakia 036 59 Slovakia (Slovak Republic)
4062	Ústredná vojenská nemocnica SNP Ružomberok – FN	Eticka komisia	Ruzomberok Slovakia 03426 Slovakia (Slovak Republic)

Center No.	Ethics Committee or Institutional Review Board	Department / Organization	Address
8000	Pharma-Ethics		123 Amcor Road<br>Lyttelton Manor 0157<br>South Africa
8003	Pharma-Ethics		123 Amcor Road<br>Lyttelton Manor 0157<br>South Africa
9050	Hospital Virgen Macarena		Sevilla Andalucia 41071<br>Spain
9051	Hospital Virgen Macarena		Sevilla Andalucia 41071<br>Spain
9052	Hospital Virgen Macarena		Sevilla Andalucia 41071<br>Spain
9053	Hospital Virgen Macarena		Sevilla Andalucia 41071<br>Spain
9055	Hospital Virgen Macarena		Sevilla Andalucia 41071<br>Spain
9056	Hospital Virgen Macarena		Sevilla Andalucia 41071<br>Spain
9057	Hospital Virgen Macarena		Sevilla Andalucia 41071<br>Spain
9059	Hospital Virgen Macarena		Sevilla Andalucia 41071<br>Spain
9060	Hospital Virgen Macarena		Sevilla Andalucia 41071<br>Spain
6000	Ethikkommission Nordwest- und Zentralschweiz (EKNZ)		Basel 4056<br>Switzerland
8021	Institutional Review Board	National Cheng Kung Univ. Hospital	Tainan 704<br>Taiwan
9080	Dokuz Eylul University	Ethics Committee	Izmir 35340<br>Turkey
9081	Dokuz Eylul University	Ethics Committee	Izmir 35340<br>Turkey
9083	Dokuz Eylul University	Ethics Committee	Izmir 35340<br>Turkey
9085	Dokuz Eylul University	Ethics Committee	Izmir 35340<br>Turkey
8040	NRES Committee South Central - Hampshire B Research Ethics Committee	Bristol REC Centre, Level 3 Block B	Bristol BS1 2NT<br>United Kingdom
8043	NRES Committee South Central - Hampshire B Research Ethics Committee	Bristol REC Centre, Level 3 Block B	Bristol BS1 2NT<br>United Kingdom
8044	NRES Committee South Central - Hampshire B Research Ethics Committee	Bristol REC Centre, Level 3 Block B	Bristol BS1 2NT<br>United Kingdom
8045	NRES Committee South Central - Hampshire B Research Ethics Committee	Bristol REC Centre, Level 3 Block B	Bristol BS1 2NT<br>United Kingdom
5001	Schulman Associates, IRB		Cincinnati<br>OH 45242<br>United States

Center No.	Ethics Committee or Institutional Review Board	Department / Organization	Address
5002	WIRB		Olympia<br>WA 98502-5010<br>United States
5004	Schulman Associates, IRB		Cincinnati<br>OH 45242<br>United States
5005	Schulman Associates, IRB		Cincinnati<br>OH 45242<br>United States
5007	Schulman Associates, IRB		Cincinnati<br>OH 45242<br>United States
5010	Schulman Associates, IRB		Cincinnati<br>OH 45242<br>United States
5012	Schulman Associates, IRB		Cincinnati<br>OH 45242<br>United States
5013	Christiana Care IRB Helen F. Graham Cancer Center & Research Institute		Newark<br>Delaware 19713<br>United States
5014	Schulman Associates, IRB		Cincinnati<br>OH 45242<br>United States
5015	Schulman Associates, IRB		Cincinnati<br>OH 45242<br>United States
5018	Schulman Associates, IRB		Cincinnati<br>OH 45242<br>United States
5019	Schulman Associates, IRB		Cincinnati<br>OH 45242<br>United States
5020	Schulman Associates, IRB		Cincinnati<br>OH 45242<br>United States
5021	Schulman Associates, IRB		Cincinnati<br>OH 45242<br>United States
5022	Schulman Associates, IRB		Cincinnati<br>OH 45242<br>United States
5026	Schulman Associates, IRB		Cincinnati<br>OH 45242<br>United States
5027	Schulman Associates, IRB		Cincinnati<br>OH 45242<br>United States
5028	WIRB		Olympia<br>WA 98502-5010<br>United States

Center No.	Ethics Committee or Institutional Review Board	Department / Organization	Address
5029	Providence Health & Service IRB		Portland<br>OR 97213<br>United States
5030	Schulman Associates, IRB		Cincinnati<br>OH 45242<br>United States
5031	WIRB		Olympia<br>WA 98502-5010<br>United States
5032	Schulman Associates, IRB		Cincinnati<br>OH 45242<br>United States
5033	Schulman Associates, IRB		Cincinnati<br>OH 45242<br>United States
5036	Schulman Associates, IRB		Cincinnati<br>OH 45242<br>United States
5038	Schulman Associates, IRB		Cincinnati<br>OH 45242<br>United States
5039	Schulman Associates, IRB		Cincinnati<br>OH 45242<br>United States
5041	Schulman Associates, IRB		Cincinnati<br>OH 45242<br>United States
5042	Schulman Associates, IRB		Cincinnati<br>OH 45242<br>United States
5049	Schulman Associates, IRB		Cincinnati<br>OH 45242<br>United States
5050	University of Colorado Health IRB		Fort Collins<br>CO 80525<br>United States
5051	Henry Ford Health System<br>Research Administration		Detroit<br>MI 48202<br>United States
5055	Schulman Associates, IRB		Cincinnati<br>OH 45242<br>United States
5056	Schulman Associates, IRB		Cincinnati<br>OH 45242<br>United States
5057	Schulman Associates, IRB		Cincinnati<br>OH 45242<br>United States
5058	Schulman Associates, IRB		Cincinnati<br>OH 45242<br>United States

Center No.	Ethics Committee or Institutional Review Board	Department / Organization	Address
5059	University of Utah IRB		Salt Lake City<br>UT 84112<br>United States
5060	Schulman Associates, IRB		Cincinnati<br>OH 45242<br>United States
5061	Schulman Associates, IRB		Cincinnati<br>OH 45242<br>United States
5064	Schulman Associates, IRB		Cincinnati<br>OH 45242<br>United States
5066	Schulman Associates, IRB		Cincinnati<br>OH 45242<br>United States
5067	Schulman Associates, IRB		Cincinnati<br>OH 45242<br>United States
5069	Univ. of New Mexico Health Sciences Office of Research		Albuquerque<br>NM 87131<br>United States
5073	Schulman Associates, IRB		Cincinnati<br>OH 45242<br>United States
5074	Schulman Associates, IRB		Cincinnati<br>OH 45242<br>United States
5075	WIRB		Olympia<br>WA 98502-5010<br>United States
5076	Schulman Associates, IRB		Cincinnati<br>OH 45242<br>United States
5079	Pro Health Care IRB Research Institute		Waukesha<br>WI 53188<br>United States
5080	Schulman Associates, IRB		Cincinnati<br>OH 45242<br>United States
5081	Schulman Associates, IRB		Cincinnati<br>OH 45242<br>United States
5089	Schulman Associates, IRB		Cincinnati<br>OH 45242<br>United States
5091	Schulman Associates, IRB		Cincinnati<br>OH 45242<br>United States
5095	Schulman Associates, IRB		Cincinnati<br>OH 45242<br>United States

Center No.	Ethics Committee or Institutional Review Board	Department / Organization	Address
5097	Schulman Associates, IRB		Cincinnati<br>OH 45242<br>United States
5098	Schulman Associates, IRB		Cincinnati<br>OH 45242<br>United States
5099	WIRB		Olympia<br>WA 98502-5010<br>United States
5100	Schulman Associates, IRB		Cincinnati<br>OH 45242<br>United States
5103	Schulman Associates, IRB		Cincinnati<br>OH 45242<br>United States
5104	Schulman Associates, IRB		Cincinnati<br>OH 45242<br>United States
5105	Schulman Associates, IRB		Cincinnati<br>OH 45242<br>United States
5110	Schulman Associates, IRB		Cincinnati<br>OH 45242<br>United States

ASSESS (CFTY720D2312)

Independent Ethics Committees (IECs) or Institutional Review Boards (IRBs) by study center

Center No.	Ethics Committee or Institutional Review Board	Department / Organization	City, State/Province, Postal Code Country
**ARGENTINA**			
0101	Comité Independiente de Ética en Investigación Biomédica- Fundación Neurológica de Buenos Aires		CABA, Buenos Aires<br>C1015ABR<br>Argentina
0106	Comité de Ética en Investigación del Complejo Médico Policial Churruca-Visca		CABA, Buenos Aires<br>Cl437JCP<br>Argentina
**BRAZIL**			
0301	Comitê de Ética em Pesquisa em Seres Humanos da Santa Casa de Misericórdia de Belo Horizonte		Belo Horizonte<br>Minas Gerais 30150-240<br>Brazil
	Comissão Nacional de Ética em Pesquisa – CONEP		Brasília<br>Distrito Federal 70750-521<br>Brazil
0302	Comitê de Ética em Pesquisa da Pontifica Universidade Católica do Rio Grande do Sul		Porto Alegre<br>Rio Grande do Sul 90610-000<br>Brazil
	Comissão Nacional de Ética em Pesquisa – CONEP		Brasília<br>Distrito Federal 70750-521<br>Brazil
0303	Comitê de Ética em Pesquisa em Seres Humanos do Hospital São José		Joinville, Santa Catarina 89202- 050<br>Brazil
	Comissão Nacional de Ética em Pesquisa – CONEP		Brasília<br>Distrito Federal 70750-521<br>Brazil

Center No.	Ethics Committee or Institutional Review Board	Department / Organization	City, State/Province, Postal Code Country
0304	Comitê de Ética em Pesquisa em Seres Humanos do Hospital Universitário Gaffrée e Guinle/ HUGG/ UNIRIO		Rio de Janeiro, Rio de Janeiro 20.270-004 Brazil
	Comissão Nacional de Etica em Pesquisa – CONEP		Brasilia Distrito Federal 70750-521 Brazil
0305	Comitê de Ética em Pesquisa – Universidade de Passo Fundo		Passo Fundo Rio Grande do Sul 99052-900 Brazil
	Comissão Nacional de Ética em Pesquisa – CONEP		Brasília Distrito Federal 70750-521 Brazil
0307	Comitê de Ética em Pesquisa em Seres Humanos Hospital das Clínicas da Universidade Federal de Goiás / GO		Goiãnia, Goiás 74605-050 Brazil
	Comissão Nacional de Ética em Pesquisa – CONEP		Brasília Distrito Federal 70750-521 Brazil
0309	Comitê de Ética em Pesquisa em Seres Humanos Hospital Israelita Albert Einstein		São Paulo, São Paulo 05652-000 Brazil
	Comissão Nacional de Ética em Pesquisa – CONEP		Brasília Distrito Federal 70750-521 Brazil
0310	Comitê de Ética em Pesquisa em Seres Humanos Hospital Angelina Caron		Campina Grande do Sul, Paraná 83.430-000 Brazil
	Comissão Nacional de Ética em Pesquisa – CONEP		Brasília Distrito Federal 70750-521 Brazil
**CANADA**			
0403	Capital Health Research Ethics Board		Halifax, Nova Scotia B3H 1V7 Canada
0404	Ottawa Health Science Network Research Ethics Board		Ottawa Ontario K1Y 4E9 Canada
0405	Health Research Ethics Board – Biomedical Panel		Edmonton Alberta T6G 1K8 Canada
0407	The McGill University Health Centre Research Ethics Board		Montreal, Quebec, H3A 2B4 Canada
	Previous: Montreal Neurological Institute and Hospital		Montreal Quebec, H3A 2B4 Canada

Center No.	Ethics Committee or Institutional Review Board	Department / Organization	City, State/Province, Postal Code Country
0408	Comité d'éthique de la recherche de l'installation de l'Hôpital Maisonneuve-Rosemont		Montréal<br>Québec H1T 2M4<br>Canada
0411	Comité d'ethique de la recherche du Centre intégré universitaire de santé et de service sociaux du Saguenay-Lac-Saint-Jean		Chicoutimi<br>Quebec G7H 5H6<br>Canada
0413	Fraser Health Authority, Department of Evaluation and Research Services		Surrey<br>British Columbia V3T 0H1<br>Canada
**CHILE**			
0501	Comité de Etica Científíco Servicio de Salud Metropolitano Central		Santiago<br>Región Metropolitana 832143<br>Chile
	Comité de Etica en Investigación Escuela de Medicina. Pontificia Universidad Católica de Chile		Santiago<br>Región Metropolitana 8330024<br>Chile
0502	Comité de Etica de la Investigación<br>Servicio de Salud Metropolitano Norte		Santiago, Chile
**MEXICO**			
1001	Comité de Ética en Investigación de Christus Muguerza del Parque S.A. de C.V.		Chihuahua, Chihuahua, CP. 31000<br>Mexico
1002	Comité de Ética en Investigacion del Grupo Médico Camino		Ciudad de México, DF 03310<br>Mexico
1004	Comité de Ética e Investigación. Unidad de Investigación en Salud de Chihuahua, S.C.		Col. San Felipe, Chihuahua 31230<br>México
1005	Comité de Ética en Investigacion. Accelerium S de R.L. de C.V.		Monterrey<br>Nuevo León 64000<br>México
1008	Comité de Ética en Investigacion. Accelerium S de R.L. de C.V.		Monterrey<br>Nuevo León 64000<br>Mexico
1009	Estimulación Magnética Transcraneal de México		Mexico City, Mexico City 11000<br>Mexico
	Previous:<br>Comite de Etica, Investigacion y Bioseguridad Privada de Aguascalientes, S.C. (CEIBIPA)		Aguascalientes, Aguascalientes 20120<br>Mexico

Center No.	Ethics Committee or Institutional Review Board	Department / Organization	City, State/Province, Postal Code Country
1010	Comité de Ética en Investigacion del Hospital Central "Dr. Ignacio Morones Prieto"		San Luis Potosí, San Luis Potosí, C.P. 78240 Mexico
1012	Comite de Ética en Investigación, Clinical Research Institute, S.C.		Tlalnepantla de Baz, Estado de México 54055 Mexico
**UNITED STATES OF AMERICA**			
5002 5006 5007 5009 5010 5012 5014 5018 5026 5027 5030 5031 5039 5040 5046 5047 5052 5053 5057 5059 5061 5063 5065 5070 5074 5075 5080 5089 5096 5098 5100 5105 5106 5108 5113 5120 5122 5126 5128 5131 5133 5134 5137 5138 5140 5143	Quorum Review IRB		Seattle, WA 98101 United States

Center No.	Ethics Committee or Institutional Review Board	Department / Organization	City, State/Province, Postal Code Country
5146<br>5147<br>5152<br>5158<br>5159<br>5163<br>5164<br>5170<br>5171<br>5175			
5017<br>5043<br>5045<br>5068<br>5072<br>5084<br>5111<br>5127<br>5162	Western Institutional Review Board		Puyallup, WA 98374-2115<br>United States<br><br>Previous Address:<br>Olympia, WA 98502<br>United States
5020	Institutional Review Board - Baylor College of Medicine		Houston, TX 77030<br>United States
5024	Henry Ford Health System	Research Administration	Detroit, MI 48202<br>United States
5025	University of California, Davis IRB Administration		Sacramento, CA 95817<br>United States
5037	Western Institutional Review Board<br><br>Previous:<br>Quorum Review IRB		Puyallup, WA 98374<br>United States<br><br>Seattle, WA 98101<br>United States
5048	Human Subjects Committee, University of Kansas Medical Center		Kansas City, KS 66160<br>United States
5054	Wayne State University Institutional Review Board		Detroit, MI 48201<br>United States
5069	SUNY Upstate Medical University IRB<br><br>Previous:<br>Institutional Review Board for the Protection of Human Subjects, SUNY Upstate Medical University		Syracuse, NY 13210<br>United States
5071	Wake Forest University Health Sciences Institutional Review Board		Winston-Salem, NC 27157<br>United States
5076	Vanderbilt Institutional Review Board		Nashville, TN 37232<br>United States
5078	Christiana Care Institutional Review Board<br>Helen F. Graham Cancer Center		Newark, DE 19713<br>United States

Center No.	Ethics Committee or Institutional Review Board	Department / Organization	City, State/Province, Postal Code Country
5082	Committee on Research Involving Human Subjects (CORIHS)<br>Melville Library, SUNY Stony Brook		Stony Brook, NY 11794-3368<br>United States
5083	Health Sciences Institutional Review Board; General Hospital		Los Angeles, CA 90033<br>United States
5085	Institutional Review Board for Health Sciences Research	University of Virginia	Charlottesville, VA 22908<br>United States
5086	University of Colorado Health Institutional Review Board / Poudre Valley Health System Institutional Review Board		Fort Collins, CO 80525<br>United States
5090	OMRF Institutional Review Board		Oklahoma City, OK 73104<br>United States
5092	Committee for the Protection of Human Subjects		Houston, TX 77030<br>United States
5094	Georgetown University Institutional Review Board		Washington, DC 20057<br>United States
5097	Northshore University Health System Institutional Review Board		Evanston, IL 60201<br>United States
5101	Saint Luke's Institutional Review Board		Kansas City, MO 64111<br>United States
5102	Western Institutional Review Board<br>Previous:<br>CentraState Medical Center Institutional Review Board		Puyallup, WA 98374-2115<br>United States<br>Freehold, NJ 07728<br>United States
5109	Indiana University Institutional Review Board	Office of Research Administration	Indianapolis, IN 46202<br>United States
5112	University at Buffalo Institutional Review Board<br>Previous:<br>Health Sciences Institutional Review Board	Clinical and Translational Research Center	Buffalo, NY 14203<br>United States<br>Buffalo, NY 14214<br>United States
5119	Mercy Medical Center Des Moines Institutional Review Board		Des Moines, IA 50314<br>United States
5121	Cleveland Clinic Institutional Board		Cleveland, OH 44195<br>United States
5123	Western Institutional Review Board<br>Previous:<br>Mercy Hospital St. Louis Institutional Review Board		Puyallup, WA 98374-2115<br>United States<br>St. Louis, MO 63141<br>United States

Center No.	Ethics Committee or Institutional Review Board	Department / Organization	City, State/Province, Postal Code Country
5124	UT Biomedical Institutional Review Board (IRB) - University of Toledo, Health Science Campus		Toledo, OH 43614<br>United States
5125	Committee on Clinical Investigations		Boston, MA 02215<br>United States
5132	Einstein Medical Center – IRB<br><br>Previous:<br>Albert Einstein Healthcare Network IRB		Philadelphia, PA 19246<br>United States<br><br>Philadelphia, PA 19141<br>United States
5136	Saint Louis University Institutional Review Board		St. Louis, MO 63104<br>United States
5142	St Joseph's Hospital and Medical Center Institutional Review Board for Human Research		Phoenix, AZ 85013<br>United States
5157	Cleveland Clinic Foundation Institutional Review Board		Cleveland, OH 44195<br>United States
5160	University of North Carolina IRB / Office of Human Research Ethics		Chapel Hill, NC 27599-7097<br>United States
5161	Mercy Health - Saint Mary's Institutional Review Board		Grand Rapids, MI 49503<br>United States
5165	Providence Health & Services Institutional Review Board		Portland, OR 97213<br>United States
5169	Ascension Wisconsin IRB<br><br>Previous:<br>Wheaton Franciscan Healthcare -IRB		Glendale, WI 53212<br>United States

EXPAND (CBAF312A2304)

Independent Ethics Committees (IEC) or Institutional Review Boards (IRB) by study center

Center No.	Ethics Committee or Institutional Review Board	Department / Organization	City, State/Province, Postal Code Country
1004	CEPI – Comité de Etica de Protocolos de Investigación	Hospital Italiano – Departamento de Docencia e Investigación	Buenos Aires, 1181, Argentina
1005	CIEIS Prof. Dr. Marcelino Rusculleda	N/A	Córdoba, X5003DCE, Argentina
1007	CEIB - Comité de Etica en Investigaciones Biomedicas	FLENI	Buenos Aires, C1428AQK, Argentina
1008	CEIB – FNBA Comité de Etica e Investigación Biomedica	Fundación Neurologica de Buenos Aires	Buenos Aires, C1015ABR, Argentina
1009	Comité Independiente de Ética Para Ensayos En Farmacologia Clinica	Fundación De Estudios Farmacologia y de Medicamentos FEFyM	Buenos Aires, C1027AAP, Argentina
1010	Comité Independiente de Ética Para Ensayos En Farmacologia Clinica	Fundación De Estudios Farmacologia y de Medicamentos FEFyM	Buenos Aires, C1027AAP, Argentina
1021	Melbourne Health Human Research Ethics Committee	Research	Melbourne, VIC, 3050, Australia
1023	Melbourne Health Human Research Ethics Committee	Research	Melbourne, VIC, 3050, Australia
1024	Melbourne Health Human Research Ethics Committee	Research	Melbourne, VIC, 3050, Australia
1025	Melbourne Health Human Research Ethics Committee	Research	Melbourne, VIC, 3050, Australia
1026	Melbourne Health Human Research Ethics Committee	Research	Melbourne, VIC, 3050, Australia
1041	Ethik-Kommission der Medizinischen Universität Wien	Ethik-Kommission	Wien, 1090, Austria
1042	Ethik-Kommission der Medizinischen Universität Wien	Ethik-Kommission	Wien, 1090, Austria
1043	Ethik-Kommission der Medizinischen Universität Wien	Ethik-Kommission	Wien, 1090, Austria
1061	Ethisch Comité UZA	Ethisch Comité	Edegem, 2650, Belgium
1062	Ethisch Comité UZA	Ethisch Comité	Edegem, 2650, Belgium

Center No.	Ethics Committee or Institutional Review Board	Department / Organization	City, State/Province, Postal Code Country
1063	Ethisch Comité UZA	Ethisch Comité	Edegem, 2650, Belgium
1064	Ethisch Comité UZA	Ethisch Comité	Edegem, 2650, Belgium
1065	Ethisch Comité UZA	Ethisch Comité	Edegem, 2650, Belgium
1066	Ethisch Comité UZA	Ethisch Comité	Edegem, 2650, Belgium
1067	Ethisch Comité UZA	Ethisch Comité	Edegem, 2650, Belgium
1068	Ethisch Comité UZA	Ethisch Comité	Edegem, 2650, Belgium
2001	Ethics Committee for Multicentre Trials	N/A	Sofia, 1000, Bulgaria
2002	Ethics Committee for Multicentre Trials	N/A	Sofia, 1000, Bulgaria
2003	Ethics Committee for Multicentre Trials	N/A	Sofia, 1000, Bulgaria
2004	Ethics Committee for Multicentre Trials	N/A	Sofia, 1000, Bulgaria
2005	Ethics Committee for Multicentre Trials	N/A	Sofia, 1000, Bulgaria
2021	McGill University Health Centre	Research Ethics Board	Montreal, Quebec, H3A 2B4, Canada
2023	Capital Health Research Ethics Board	N/A	Halifax, NS, B3H 1V7, Canada
2024	Health Research Ethics Board	N/A	Edmonton, AB, T6G 1K8, Canada
2026	Queen's University Health Sciences and Affiliated Teaching Hospitals	Research Ethics Board	Kingston, Ontario, K7L 3N6, Canada
2028	Centre hospitalier de l'université de Montréal	Comité d'éthique de la recherche	Montréal, QC, H2W 1Y5, Canada
2029	Ottawa Health Science Network Research Ethics Board	N/A	Ottawa, ON, K1Y 4E9, Canada
2030	IRB Services	N/A	Aurora, Ontario, L4G 0A5, Canada
2031	Sunnybrook	Research Ethics Board	Toronto, Ontario, M4N 3M5, Canada
2036	Fraser Health Research Ethics board	FHA evaluation and research services	Surrey, BC, V3T 0H1, Canada
2038	Coinjoint Health Research ethics board	N/A	Calgary, AB, T2N 1N4, Canada
4060	Peking Union Medical College Hospital	IRB	Beijing, Beijing, 100010, China
4061	IRB of Beijing Hospital	IRB	Beijing, Beijing, 100730, China
4062	Chinese PLA General Hospital	IRB	Beijing, Beijing, 100853, China
4066	Peking University First Hospital	Beijing	Beijing, Beijing, 100034, China
4068	Huashan Hospital Affiliated to Fudan University	IRB	Shanghai, Shanghai, 200040, China
4070	The First Hospital of Jilin University	IRB	Changchun, Jilin, 130021, China
4071	IRB of West China Hospital	IRB	Chengdu, Sichuan, 610041, China

4072	Tangdu Hospital Affiliated to The Fourth Military Medical University of PLA	IRB	Xian, Shanxi, 710038, China
4073	The First Hospital of Shanxi Medical University	IRB	Taiyuan, Shanxi, 030001, China

**Center No.**	**Ethics Committee or Institutional Review Board**	**Department / Organization**	**City, State/Province, Postal Code Country**
2051	Eticka komise Vseobecne fakultni nemocnice v Praze	N/A	Praha 2, 128 08, Czech Republic
2052	Eticka komise pro multicentricke klinicke hodnoceni Fakultni nemocnice v Motole	N/A	Praha 5, 150 06 , Czech Republic
2053	Eticka komise Krajska zdravotni,<br>a.s. - Nemocnice Teplice	N/A	Teplice, 415 29, Czech Republic
2054	Eticka komise Fakultni nemocnice Brno	N/A	Brno, 625 00, Czech Republic
2055	Eticka komise Nemocnice Jihlava	N/A	Jihlava, 586 33, Czech Republic
2071	Tallinn Medical Research Ethics Committee (TMREC)	National Institute for Health Development	Tallinn, 11619, Estonia
2072	Tallinn Medical Research Ethics Committee (TMREC)	National Institute for Health Development	Tallinn, 11619, Estonia
3001	Comité de Protection des Personnes Est III	Hôpital de Brabois	Vandoeuvre-Les-Nancy, 54511, France
3002	Comité de Protection des Personnes Est III	Hôpital de Brabois	Vandoeuvre-Les-Nancy, 54511, France
3003	Comité de Protection des Personnes Est III	Hôpital de Brabois	Vandoeuvre-Les-Nancy, 54511, France
3004	Comité de Protection des Personnes Est III	Hôpital de Brabois	Vandoeuvre-Les-Nancy, 54511, France
3005	Comité de Protection des Personnes Est III	Hôpital de Brabois	Vandoeuvre-Les-Nancy, 54511, France
3006	Comité de Protection des Personnes Est III	Hôpital de Brabois	Vandoeuvre-Les-Nancy, 54511, France
3007	Comité de Protection des Personnes Est III	Hôpital de Brabois	Vandoeuvre-Les-Nancy, 54511, France
3009	Comité de Protection des Personnes Est III	Hôpital de Brabois	Vandoeuvre-Les-Nancy, 54511, France
3010	Comité de Protection des Personnes Est III	Hôpital de Brabois	Vandoeuvre-Les-Nancy, 54511, France
3011	Comité de Protection des Personnes Est III	Hôpital de Brabois	Vandoeuvre-Les-Nancy, 54511, France
3012	Comité de Protection des Personnes Est III	Hôpital de Brabois	Vandoeuvre-Les-Nancy, 54511, France
4001	Ethikkommission der Ärztekammer Westfalen-Lippe und der Medizinischen Fakultät der Westfälischen Wilhelms-	N/A	Münster, Germany, 48147, Germany

	Universität Münster		
4002	Ethikkommission der Ärztekammer Westfalen-Lippe und der Medizinischen Fakultät der Westfälischen Wilhelms-<br>Universität Münster	N/A	Münster, Germany, 48147, Germany

Center No.	Ethics Committee or Institutional Review Board	Department / Organization	City, State/Province, Postal Code Country
4003	Ethikkommission der Ärztekammer Westfalen-Lippe und der Medizinischen Fakultät der Westfälischen Wilhelms- Universität Münster	N/A	Münster, Germany, 48147, Germany
4004	Ethikkommission der Ärztekammer Westfalen-Lippe und der Medizinischen Fakultät der Westfälischen Wilhelms-<br>Universität Münster	N/A	Münster, Germany, 48147, Germany
4005	Ethikkommission der Ärztekammer Westfalen-Lippe und der Medizinischen Fakultät der Westfälischen Wilhelms-<br>Universität Münster	N/A	Münster, Germany, 48147, Germany
4006	Ethikkommission der Ärztekammer Westfalen-Lippe und der Medizinischen Fakultät der Westfälischen Wilhelms-<br>Universität Münster	N/A	Münster, Germany, 48147, Germany
4007	Ethikkommission der Ärztekammer Westfalen-Lippe und der Medizinischen Fakultät der Westfälischen Wilhelms-<br>Universität Münster	N/A	Münster, Germany, 48147, Germany
4008	Ethikkommission der Ärztekammer Westfalen-Lippe und der Medizinischen Fakultät der Westfälischen Wilhelms-<br>Universität Münster	N/A	Münster, Germany, 48147, Germany
4009	Ethikkommission der Ärztekammer Westfalen-Lippe und der Medizinischen Fakultät der Westfälischen Wilhelms-<br>Universität Münster	N/A	Münster, Germany, 48147, Germany
4010	Ethikkommission der Ärztekammer Westfalen-Lippe und der Medizinischen Fakultät der Westfälischen Wilhelms- Universität Münster	N/A	Münster, Germany, 48147, Germany

4011	Ethikkommission der Ärztekammer Westfalen-Lippe und der Medizinischen Fakultät der Westfälischen Wilhelms-<br><br>Universität Münster	N/A	Münster, Germany, 48147, Germany
4012	Ethikkommission der Ärztekammer Westfalen-Lippe und der Medizinischen Fakultät der Westfälischen Wilhelms- Universität Münster	N/A	Münster, Germany, 48147, Germany

Center No.	Ethics Committee or Institutional Review Board	Department / Organization	City, State/Province, Postal Code Country
4013	Ethikkommission der Ärztekammer Westfalen-Lippe und der Medizinischen Fakultät der Westfälischen Wilhelms- Universität Münster	N/A	Münster, Germany, 48147, Germany
4015	Ethikkommission der Ärztekammer Westfalen-Lippe und der Medizinischen Fakultät der Westfälischen Wilhelms-<br><br>Universität Münster	N/A	Münster, Germany, 48147, Germany
4016	Ethikkommission der Ärztekammer Westfalen-Lippe und der Medizinischen Fakultät der Westfälischen Wilhelms-<br><br>Universität Münster	N/A	Münster, Germany, 48147, Germany
4017	Ethikkommission der Ärztekammer Westfalen-Lippe und der Medizinischen Fakultät der Westfälischen Wilhelms-<br><br>Universität Münster	N/A	Münster, Germany, 48147, Germany
4018	Ethikkommission der Ärztekammer Westfalen-Lippe und der Medizinischen Fakultät der Westfälischen Wilhelms-<br><br>Universität Münster	N/A	Münster, Germany, 48147, Germany
4019	Ethikkommission der Ärztekammer Westfalen-Lippe und der Medizinischen Fakultät der Westfälischen Wilhelms-<br><br>Universität Münster	N/A	Münster, Germany, 48147, Germany
4022	Ethikkommission der Ärztekammer Westfalen-Lippe und der Medizinischen Fakultät der Westfälischen Wilhelms-<br><br>Universität Münster	N/A	Münster, Germany, 48147, Germany
4023	Ethikkommission der Ärztekammer Westfalen-Lippe und der Medizinischen Fakultät der Westfälischen Wilhelms- Universität Münster	N/A	Münster, Germany, 48147, Germany

Center No.	Ethics Committee or Institutional Review Board	Department / Organization	City, State/Province, Postal Code Country
4024	Ethikkommission der Ärztekammer Westfalen-Lippe und der Medizinischen Fakultät der Westfälischen Wilhelms- Universität Münster	N/A	Münster, Germany, 48147, Germany
4025	Ethikkommission der Ärztekammer Westfalen-Lippe und der Medizinischen Fakultät der Westfälischen Wilhelms- Universität Münster	N/A	Münster, Germany, 48147, Germany
4026	Ethikkommission der Ärztekammer Westfalen-Lippe und der Medizinischen Fakultät der Westfälischen Wilhelms- Universität Münster	N/A	Münster, Germany, 48147, Germany
4027	Ethikkommission der Ärztekammer Westfalen-Lippe und der Medizinischen Fakultät der Westfälischen Wilhelms- Universität Münster	N/A	Münster, Germany, 48147, Germany
4028	Ethikkommission der Ärztekammer Westfalen-Lippe und der Medizinischen Fakultät der Westfälischen Wilhelms- Universität Münster	N/A	Münster, Germany, 48147, Germany
4029	Ethikkommission der Ärztekammer Westfalen-Lippe und der Medizinischen Fakultät der Westfälischen Wilhelms- Universität Münster	N/A	Münster, Germany, 48147, Germany
4030	Ethikkommission der Ärztekammer Westfalen-Lippe und der Medizinischen Fakultät der Westfälischen Wilhelms- Universität Münster	N/A	Münster, Germany, 48147, Germany
4031	Ethikkommission der Ärztekammer Westfalen-Lippe und der Medizinischen Fakultät der Westfälischen Wilhelms- Universität Münster	N/A	Münster, Germany, 48147, Germany
4032	Ethikkommission der Ärztekammer Westfalen-Lippe und der Medizinischen Fakultät der Westfälischen Wilhelms- Universität Münster	N/A	Münster, Germany, 48147, Germany
4033	Ethikkommission der Ärztekammer Westfalen-Lippe und der Medizinischen Fakultät der Westfälischen Wilhelms- Universität Münster	N/A	Münster, Germany, 48147, Germany

4034	Ethikkommission der Ärztekammer Westfalen-Lippe und der Medizinischen Fakultät der Westfälischen Wilhelms-<br><br>Universität Münster	N/A	Münster, Germany, 48147, Germany
4035	Ethikkommission der Ärztekammer Westfalen-Lippe und der Medizinischen Fakultät der Westfälischen Wilhelms- Universität Münster	N/A	Münster, Germany, 48147, Germany

Center No.	Ethics Committee or Institutional Review Board	Department / Organization	City, State/Province, Postal Code Country
4036	Ethikkommission der Ärztekammer Westfalen-Lippe und der Medizinischen Fakultät der Westfälischen Wilhelms- Universität Münster	N/A	Münster, Germany, 48147, Germany
4037	Ethikkommission der Ärztekammer Westfalen-Lippe und der Medizinischen Fakultät der Westfälischen Wilhelms-<br><br>Universität Münster	N/A	Münster, Germany, 48147, Germany
4038	Ethikkommission der Ärztekammer Westfalen-Lippe und der Medizinischen Fakultät der Westfälischen Wilhelms-<br><br>Universität Münster	N/A	Münster, Germany, 48147, Germany
4039	Ethikkommission der Ärztekammer Westfalen-Lippe und der Medizinischen Fakultät der Westfälischen Wilhelms-<br><br>Universität Münster	N/A	Münster, Germany, 48147, Germany
4040	Ethikkommission der Ärztekammer Westfalen-Lippe und der Medizinischen Fakultät der Westfälischen Wilhelms-<br><br>Universität Münster	N/A	Münster, Germany, 48147, Germany
4042	Ethikkommission der Ärztekammer Westfalen-Lippe und der Medizinischen Fakultät der Westfälischen Wilhelms-<br><br>Universität Münster	N/A	Münster, Germany, 48147, Germany
4043	Ethikkommission der Ärztekammer Westfalen-Lippe und der Medizinischen Fakultät der Westfälischen Wilhelms-<br><br>Universität Münster	N/A	Münster, Germany, 48147, Germany
4044	Ethikkommission der Ärztekammer Westfalen-Lippe und der Medizinischen Fakultät der Westfälischen Wilhelms- Universität Münster	N/A	Münster, Germany, 48147, Germany

4045	Ethikkommission der Ärztekammer Westfalen-Lippe und der Medizinischen Fakultät der Westfälischen Wilhelms-<br><br>Universität Münster	N/A	Münster, Germany, 48147, Germany
4046	Ethikkommission der Ärztekammer Westfalen-Lippe und der Medizinischen Fakultät der Westfälischen Wilhelms- Universität Münster	N/A	Münster, Germany, 48147, Germany

Center No.	Ethics Committee or Institutional Review Board	Department / Organization	City, State/Province, Postal Code Country
4047	Ethikkommission der Ärztekammer Westfalen-Lippe und der Medizinischen Fakultät der Westfälischen Wilhelms- Universität Münster	N/A	Münster, Germany, 48147, Germany
4048	Ethikkommission der Ärztekammer Westfalen-Lippe und der Medizinischen Fakultät der Westfälischen Wilhelms-<br><br>Universität Münster	N/A	Münster, Germany, 48147, Germany
4049	Ethikkommission der Ärztekammer Westfalen-Lippe und der Medizinischen Fakultät der Westfälischen Wilhelms-<br><br>Universität Münster	N/A	Münster, Germany, 48147, Germany
3031	National Ethics Committee	N/A	Athens, GR-15562, Greece
3032	National Ethics Committee	N/A	Athens, GR-15562, Greece
3033	National Ethics Committee	N/A	Athens, GR-15562, Greece
3051	Medical Research Council, Ethics Committee for Clinical Pharmacology (ECCP)	Central Ethics Committee	Budapest, Arany J. u. 6-8., 1051, Hungary
3053	Medical Research Council, Ethics Committee for Clinical Pharmacology (ECCP)	Central Ethics Committee	Budapest, Arany J. u. 6-8., 1051, Hungary
3054	Medical Research Council, Ethics Committee for Clinical Pharmacology (ECCP)	Central Ethics Committee	Budapest, Arany J. u. 6-8., 1051, Hungary
3055	Medical Research Council, Ethics<br><br>Committee for Clinical Pharmacology (ECCP)	Central Ethics Committee	Budapest, Arany J. u. 6-8., 1051, Hungary
3056	Medical Research Council, Ethics Committee for Clinical<br><br>Pharmacology (ECCP)	Central Ethics Committee	Budapest, Arany J. u. 6-8., 1051, Hungary
3057	Medical Research Council, Ethics Committee for Clinical<br><br>Pharmacology (ECCP)	Central Ethics Committee	Budapest, Arany J. u. 6-8., 1051, Hungary

3058	Medical Research Council, Ethics Committee for Clinical Pharmacology (ECCP)	Central Ethics Committee	Budapest, Arany J. u. 6-8., 1051, Hungary
3059	Medical Research Council, Ethics Committee for Clinical Pharmacology (ECCP)	Central Ethics Committee	Budapest, Arany J. u. 6-8., 1051, Hungary
3060	Medical Research Council, Ethics Committee for Clinical Pharmacology (ECCP)	Central Ethics Committee	Budapest, Arany J. u. 6-8., 1051, Hungary
3061	Medical Research Council, Ethics Committee for Clinical Pharmacology (ECCP)	Central Ethics Committee	Budapest, Arany J. u. 6-8., 1051, Hungary
3062	Medical Research Council, Ethics Committee for Clinical Pharmacology (ECCP)	Central Ethics Committee	Budapest, Arany J. u. 6-8., 1051, Hungary

**Center No.**	**Ethics Committee or Institutional Review Board**	**Department / Organization**	**City, State/Province, Postal Code Country**
3071	St Vincent's Healthcare	Ethics and Medical Research Committee	Elm Park, Dublin 4, Ireland
3072	St Vincent's Healthcare	Ethics and Medical Research Committee	Elm Park, Dublin 4, Ireland
3090	The Chaim Sheba Medical Center Ethics Commitee	N/A	Ramat Gan, 52621, Israel
3091	Hadassah Medical Organization Ethics Committee	N/A	Jerusalem, 91120, Israel
7001	Comitato etico dell'irccs ospedale san raffaele di milano	N/A	Milano, 20132, Italy
7002	Comitato etico dell'universita' cattolica del s. Cuore	N/A	Roma, 00168, Italy
7003	Comitato Etico dell'Università Sapienza	N/A	Roma, 00161, Italy
7004	Comitato etico Lazio 1 presso azienda ospedaliera san	N/A	Roma, 00152, Italy
7005	Comitato etico indipendente presso la fondazione PTV	N/A	Roma, 00133, Italy
7006	Fond. IRCCS CA' Granda	Ospedale Maggiore Policlinico	Milano, 20122, Italy
7007	Comitato etico indipendente dell'irccs Fondazione Santa	N/A	Roma, 00179, Italy
7008	Comitato etico per le attivita' biomediche carlo romanodell'universita' degli studi federico ii di Napoli	N/A	Napoli, 80131, Italy
7009	Comitato Etico dell'Az. Ospedaliero-Univeristaria San Martino di Genova	N/A	Genova, 16132, Italy
7010	Comitato etico Palermo 1 c/o AOU	N/A	Palermo, 90127, Italy
6050	Aomori Prefectural Central Hospital IRB	N/A	Aomori-city, Aomori, 030-8553, Japan

6052	Keio University Hospital IRB	N/A	Shinjuku-ku, Tokyo, 160-8582, Japan
6054	Chiba University Hospital IRB	N/A	Chiba-city, Chiba, 260-8677, Japan
6055	Ehime University Hospital IRB	N/A	Toon-city, Ehime, 791-0295, Japan
6056	Osaka University Hospital IRB	N/A	Suita-city, Osaka, 565-0871, Japan
6057	Iwate Medical University Hospital IRB	N/A	Morioka-city, Iwate, 020-8505, Japan
6058	Tokyo Woman's Medical University Hospital IRB	N/A	Shinjuku-ku, Tokyo, 162-8666, Japan
6060	Sone Clinic IRB	Sone Clinic	Shinjuku-ku, Tokyo, Japan
6061	Kyoto Min-iren Chuo Hospital IRB	N/A	Kyoto-city, Kyoto, 604-8453, Japan
6063	Saitama Medical Center IRB	N/A	Kawagoe-city, Saitama, 350-8550, Japan
6064	Ebara Hospital IRB	N/A	Ota-ku, Tokyo, 145-0065 , Japan
6065	National Hospital Organization Asahikawa Medical Center IRB	N/A	Asahikawa-city, Hokkaido, 070-8644, Japan

**Center No.**	**Ethics Committee or Institutional Review Board**	**Department / Organization**	**City, State/Province, Postal Code Country**
6067	National Center of Neurology and Psychiatry IRB	N/A	Kodaira-city, Tokyo, 187-8551, Japan
6068	National Defence Medical College Hospital IRB	N/A	Tokorozawa-city, Saitama, 359-8513, Japan
7031	Pauls Stradins Clinical University Hospital Development Society	N/A	Riga, LV-1002, Latvia
7032	Pauls Stradins Clinical University Hospital Development Society	N/A	Riga, LV-1002, Latvia
7033	Pauls Stradins Clinical University Hospital Development Society	N/A	Riga, LV-1002, Latvia
7041	Lithuanian Bioethics Committee	Central EC for Lithuania	Vilnius, LT-01128, Lithuania
7042	Lithuanian Bioethics Committee	Central EC for Lithuania	Vilnius, LT-01128, Lithuania
7043	Lithuanian Bioethics Committee	Central EC for Lithuania	Vilnius, LT-01128, Lithuania
7061	VUmc	Raad van Bestuur	Amsterdam, 1081 BT, Netherlands
7062	Orbis Medisch Centrum	Directie	Sittard-Geleen, 6162 BG, Netherlands
7063	Catharina Ziekenhuis	METC	Eindhoven, 5623 EJ, Netherlands
7064	Elisabeth-TweeSteden Ziekenhuis	Raad van Bestuur	Tilburg, 5022 GC, Netherlands
7067	Amphia Ziekenhuis	Directiecomite	Breda, 4818 CK, Netherlands
7069	Westfriesgasthuis	Raad van Bestuur	Hoorn, 1624 NP, Netherlands
7081	Komisja Bioetyki Uniwersytetu Medycznego w Łodzi	N/A	Łódź, 90419, Poland
7082	Komisja Bioetyki Uniwersytetu Medycznego w Łodzi	N/A	Łódź, 90419, Poland
7084	Komisja Bioetyki Uniwersytetu Medycznego w Łodzi	N/A	Łódź, 90419, Poland

7085	Komisja Bioetyki Uniwersytetu Medycznego w Łodzi	N/A	Łódź, 90419, Poland
7086	Komisja Bioetyki Uniwersytetu Medycznego w Łodzi	N/A	Łódź, 90419, Poland
7087	Komisja Bioetyki Uniwersytetu Medycznego w Łodzi	N/A	Łódź, 90419, Poland
7088	Komisja Bioetyki Uniwersytetu Medycznego w Łodzi	N/A	Łódź, 90419, Poland
7089	Komisja Bioetyki Uniwersytetu Medycznego w Łodzi	N/A	Łódź, 90419, Poland
8001	Comissão de Ética para a Investigação Clínica	N/A	Lisboa, 1749-004, Portugal
8002	Comissão de Ética para a Investigação Clínica	N/A	Lisboa, 1749-004, Portugal
8003	Comissão de Ética para a Investigação Clínica	N/A	Lisboa, 1749-004, Portugal
8004	Comissão de Ética para a Investigação Clínica	N/A	Lisboa, 1749-004, Portugal
8005	Comissão de Ética para a Investigação Clínica	N/A	Lisboa, 1749-004, Portugal

**Center No.**	**Ethics Committee or Institutional Review Board**	**Department / Organization**	**City, State/Province, Postal Code Country**
8021	Comisia Nationala de Etica pentru Studiul Clinic al Medicamentului	N/A	Bucharest, 011478, Romania
8022	Comisia Nationala de Etica pentru Studiul Clinic al Medicamentului	N/A	Bucharest, 011478, Romania
8023	Comisia Nationala de Etica pentru Studiul Clinic al Medicamentului	N/A	Bucharest, 011478, Romania
8024	Comisia Nationala de Etica pentru Studiul Clinic al Medicamentului	N/A	Bucharest, 011478, Romania
8025	Comisia Nationala de Etica pentru Studiul Clinic al Medicamentului	N/A	Bucharest, 011478, Romania
8027	Comisia Nationala de Etica pentru Studiul Clinic al Medicamentului	N/A	Bucharest, 011478, Romania
8028	Comisia Nationala de Etica pentru Studiul Clinic al Medicamentului	N/A	Bucharest, 011478, Romania
8041	LEC of State Budgetary Healthcare institution "Regional Clinical Hospital"	N/A	Barnaul, 656024, Russia
8042	Local Ethic Committee of Bashkir State Medical University	N/A	UFA, 450005, Russia
8044	Local Committee of Institute of the Human Brain of the Russian Academy of Sciences	N/A	Saint-Petersburg, 197376, Russia
8045	Local Committee on Ethics at City Clinical Hospital #1 named after N.i. Pirogov	N/A	Moscow, 119049, Russia

8046	LEC of Millitary Medical academy<br><br>n.a. S.M.Kirov	N/A	Saint-Petersburg, 194044, Russia
8048	Local Committee of City Clinical Hospital #31	N/A	Saint-Petersburg, 197110, Russia
8050	Independent Ethics Committee at State Budget Healthcare Institution of Moscow City "City Clinical<br><br>Hospital #11 HD of Moscow"	N/A	Moscow, 127018, Russia
8051	Local Ethic Committee of Siberian District Medical Centre of Federal Medical-Biological Agency of Russia	N/A	Novosibirsk, 630007, Russia
8052	Local Ethics Commitee at Republican Clinical Hospital of Rehabilitation Treatment of Ministry of Health of Tatarstan<br><br>Republic	N/A	Kazan, 420021, Russia
8071	EK FN a LF UK Bratislava	N/A	Bratislava, 813 69 , Slovakia (Slovak Republic)
8072	Eticka komisia UN Bratislava	N/A	Bratislava, 821 01, Slovakia (Slovak Republic)
8073	Eticka komisia FN Trnava	N/A	Trnava, 917 75, Slovakia (Slovak Republic)

Center No.	Ethics Committee or Institutional Review Board	Department / Organization	City, State/Province, Postal Code Country
8074	Eticka Komisia pri FNsP F.D.Roosevelta	N/A	Banska Bystrica, 97517, Slovakia (Slovak Republic)
8075	Etická komisia Univerzitnej nemocnice Martin	N/A	Martin, 036 59, Slovakia (Slovak Republic)
8076	Etická komisia pri FN Nitra	N/A	Nitra, 950 01 , Slovakia (Slovak Republic)
8077	Eticka komisia FNsP J.A.Reimana	N/A	Presov, 08001, Slovakia (Slovak Republic)
8078	Eticka komisia FNsP J.A.Reimana	N/A	Presov, 08001, Slovakia (Slovak Republic)
9001	CEIC Hospital Univ. Vall D’Hebron	13-Hospital<br><br>Maternoinfantil Institut Recerca HUVH	Barcelona, 08035, Spain
9002	CEIC Hospital Univ. Vall D’Hebron	13-Hospital<br><br>Maternoinfantil Institut Recerca HUVH	Barcelona, 08035, Spain
9003	CEIC Hospital Univ. Vall D’Hebron	13-Hospital Maternoinfantil Institut<br><br>Recerca HUVH	Barcelona, 08035, Spain
9004	CEIC Hospital Univ. Vall D’Hebron	13-Hospital Maternoinfantil Institut Recerca HUVH	Barcelona, 08035, Spain

9005	CEIC Hospital Univ. Vall D'Hebron	13-Hospital Maternoinfantil Institut Recerca HUVH	Barcelona, 08035, Spain
9006	CEIC Hospital Univ. Vall D'Hebron	13-Hospital Maternoinfantil Institut Recerca HUVH	Barcelona, 08035, Spain
9007	CEIC Hospital Univ. Vall D'Hebron	13-Hospital Maternoinfantil Institut Recerca HUVH	Barcelona, 08035, Spain
9008	CEIC Hospital Univ. Vall D'Hebron	13-Hospital Maternoinfantil Institut Recerca HUVH	Barcelona, 08035, Spain
9009	CEIC Hospital Univ. Vall D'Hebron	13-Hospital Maternoinfantil Institut Recerca HUVH	Barcelona, 08035, Spain
9011	CEIC Hospital Univ. Vall D'Hebron	13-Hospital Maternoinfantil Institut Recerca HUVH	Barcelona, 08035, Spain
9012	CEIC Hospital Univ. Vall D'Hebron	13-Hospital Maternoinfantil Institut Recerca HUVH	Barcelona, 08035, Spain
9013	CEIC Hospital Univ. Vall D'Hebron	13-Hospital Maternoinfantil Institut Recerca HUVH	Barcelona, 08035, Spain
9014	CEIC Hospital Univ. Vall D'Hebron	13-Hospital Maternoinfantil Institut Recerca HUVH	Barcelona, 08035, Spain

Center No.	Ethics Committee or Institutional Review Board	Department / Organization	City, State/Province, Postal Code Country
9015	CEIC Hospital Univ. Vall D'Hebron	13-Hospital Maternoinfantil Institut Recerca HUVH	Barcelona, 08035, Spain
9016	CEIC Hospital Univ. Vall D'Hebron	13-Hospital Maternoinfantil Institut Recerca HUVH	Barcelona, 08035, Spain
9031	Regionala etikprövningsnämnden i Stockholm	FE289	Stockholm, 17177, Sweden
9032	Regionala etikprövningsnämnden i Stockholm	FE289	Stockholm, 17177, Sweden
9033	Regionala etikprövningsnämnden i Stockholm	FE289	Stockholm, 17177, Sweden
9041	Ethics comittee EKNZ	N/A	Basel , Basel, 4056, Switzerland
9042	Kantonale Ethikkommission Zürich	N/A	Zürich, 8090, Switzerland
9043	Comission Cantonale d'éthique	N/A	Lausanne, Vaud, 1012, Switzerland
9044	Ethics comittee EKNZ	N/A	Basel , Basel, 4056, Switzerland
9045	Ethikkommission des kanton St Gallen	N/A	St Gallen, 9007, Switzerland
9046	Comitato etico cantonale	N/A	Bellinzona, 6501, Switzerland
9047	Ethics comittee EKNZ	N/A	Basel , Basel, 4056, Switzerland

9051	Kocaeli University Clinical Research Ethics Committee	Kocaeli University Medical Faculty	Kocaeli, Turkey, 41380 , Turkey
9052	Kocaeli University Clinical Research Ethics Committee	Kocaeli University Medical Faculty	Kocaeli, Turkey, 41380 , Turkey
9053	Kocaeli University Clinical Research Ethics Committee	Kocaeli University Medical Faculty	Kocaeli, Turkey, 41380 , Turkey
9054	Kocaeli University Clinical Research Ethics Committee	Kocaeli University Medical Faculty	Kocaeli, Turkey, 41380 , Turkey
9055	Kocaeli University Clinical Research Ethics Committee	Kocaeli University Medical Faculty	Kocaeli, Turkey, 41380 , Turkey
9056	Kocaeli University Clinical Research Ethics Committee	Kocaeli University Medical Faculty	Kocaeli, Turkey, 41380 , Turkey
9057	Kocaeli University Clinical Research Ethics Committee	Kocaeli University Medical Faculty	Kocaeli, Turkey, 41380 , Turkey
9062	NRES Committee South Central - Southampton B	Bristol REC Centre, Level 3 Block B	Bristol, BS1 2NT, United Kingdom
9063	NRES Committee South Central - Southampton B	Bristol REC Centre, Level 3 Block B	Bristol, BS1 2NT, United Kingdom
9064	NRES Committee South Central - Southampton B	Bristol REC Centre, Level 3 Block B	Bristol, BS1 2NT, United Kingdom
9065	NRES Committee South Central - Southampton B	Bristol REC Centre, Level 3 Block B	Bristol, BS1 2NT, United Kingdom
9066	NRES Committee South Central - Southampton B	Bristol REC Centre, Level 3 Block B	Bristol, BS1 2NT, United Kingdom
9068	NRES Committee South Central - Southampton B	Bristol REC Centre, Level 3 Block B	Bristol, BS1 2NT, United Kingdom

**Center No.**	**Ethics Committee or Institutional Review Board**	**Department / Organization**	**City, State/Province, Postal Code Country**
9069	NRES Committee South Central - Southampton B	Bristol REC Centre, Level 3 Block B	Bristol, BS1 2NT, United Kingdom
9070	NRES Committee South Central - Southampton B	Bristol REC Centre, Level 3 Block B	Bristol, BS1 2NT, United Kingdom
9071	NRES Committee South Central - Southampton B	Bristol REC Centre, Level 3 Block B	Bristol, BS1 2NT, United Kingdom
9072	NRES Committee South Central - Southampton B	Bristol REC Centre, Level 3 Block B	Bristol, BS1 2NT, United Kingdom
5001	Quorum Review, Inc.	N/A	Seattle, WA, 98101, United States
5004	University of Vermont Research Protections Office	Office pf Sponsored Programs	Bjrlington, VT, 05401, United States
5005	Quorum Review, Inc.	N/A	Seattle, WA, 98101, United States
5006	Quorum Review, Inc.	N/A	Seattle, WA, 98101, United States
5009	WIRB	N/A	Olympia, WA, 98502-5010, United States
5011	Lifespan IRB	N/A	Providence, RI, 02903, United States
5013	Office of Regulatory Affairs	IRB	Philadelphia, PA, 19104, United States

5015	Quorum Review, Inc.	N/A	Seattle, WA, 98101, United States
5017	Quorum Review, Inc.	N/A	Seattle, WA, 98101, United States
5018	Quorum Review, Inc.	N/A	Seattle, WA, 98101, United States
5021	WIRB	N/A	Olympia, WA, 98502-5010, United States
5022	UC Davis Office of Research	IRB Administration	Sacramento, CA, 95817, United States
5028	Committees on Research Involving Human Subjects	Office of Research Compliance	Stony Brook, NY, 11794-3368, United States
5030	Quorum Review, Inc.	N/A	Seattle, WA, 98101, United States
5032	Cleveland Clinic Institutional Review Board	N/A	Cleveland, OH, 44195, United States
5035	Alta Bates Summit IRB	N/A	Berkeley, CA, 94705, United States
5036	Quorum Review, Inc.	N/A	Seattle, WA, 98101, United States
5042	WIRB	N/A	Olympia, WA, 98502-5010, United States
5043	Quorum Review, Inc.	N/A	Seattle, WA, 98101, United States
5047	Providence Health & Services IRB	N/A	Portland, OR, 97225, United States
5052	Wayne State University IRB	N/A	Detroit, MI, 48201, United States
5054	NorthShore University Healthsystem IRB	N/A	Evanston, IL, 60201, United States
5056	Henry Ford Hospital IRB	N/A	Detroit, MI, 48202, United States
5057	Quorum Review, Inc.	N/A	Seattle, WA, 98101, United States
5059	University of Michigan Medical School IRB	N/A	Ann Arbor, MI, 48109, United States
5062	Quorum Review, Inc.	N/A	Seattle, WA, 98101, United States
5063	NYU School of Medicine	IRB Human Research Protection Program	New York, NY, 10016, United States

Center No.	Ethics Committee or Institutional Review Board	Department / Organization	City, State/Province, Postal Code Country
5068	Johns Hopkins Institutional Review Board	N/A	Baltimore, MD, 21205-1911, United States
5069	Quorum Review, Inc.	N/A	Seattle, WA, 98101, United States
5071	Copernicus Group IRB	N/A	Durham, NC, 27713, United States
5073	Quorum Review, Inc.	N/A	Seattle, WA, 98101, United States
5074	Quorum Review, Inc.	N/A	Seattle, WA, 98101, United States
5075	Quorum Review, Inc.	N/A	Seattle, WA, 98101, United States
5079	Quorum Review, Inc.	N/A	Seattle, WA, 98101, United States
5080	Quorum Review, Inc.	N/A	Seattle, WA, 98101, United States
5085	Wheaton Franciscan Healthcare IRB	N/A	Glendale, WI, 53212, United States
5088	Quorum Review, Inc.	N/A	Seattle, WA, 98101, United States
5092	Quorum Review, Inc.	N/A	Seattle, WA, 98101, United States
5096	University of Southern California Health Sciences Campus IRB	N/A	Los Angeles, CA, 90033, United States
5099	Rush University Medical Center	N/A	Chicago, IL, 60612, United States
5100	VA North Texas Health Care System IRB#1	N/A	Dallas, TX, 75216, United States
5101	WIRB	N/A	Olympia, WA, 98502-5010, United States
5103	Quorum Review, Inc.	N/A	Seattle, WA, 98101, United States
5108	Quorum Review, Inc.	N/A	Seattle, WA, 98101, United States
5109	Quorum Review, Inc.	N/A	Seattle, WA, 98101, United States
5112	Quorum Review, Inc.	N/A	Seattle, WA, 98101, United States
5114	Quorum Review, Inc.	N/A	Seattle, WA, 98101, United States
5115	Quorum Review, Inc.	N/A	Seattle, WA, 98101, United States
5117	Quorum Review, Inc.	N/A	Seattle, WA, 98101, United States
5118	CentraState IRB	N/A	Freehold, NJ, 07728, United States
5123	Quorum Review, Inc.	N/A	Seattle, WA, 98101, United States
5125	Quorum Review, Inc.	N/A	Seattle, WA, 98101, United States

FREEDOMS I (FTY720D 2301)

Independent Ethics Committees or Institutional Review Boards by study center

Center No.	Ethics Committee or Institutional Review Board	Address	Country
252	Human Research Ethics Committee - D St Vincent's Health Melbourne	PO Box 2900<br>St Vincent's Health<br>3065<br>Fitzroy<br>Vic	Australia
251	Austin Health Human Research Ethics Committee	300 Waterdale Road<br>Research Ethics Unit<br>Ground Floor<br>North Wing<br>3081<br>Heidelberg West<br>Vic	Australia
253	Central Northern Adelaide Health Services Ethics of Human Research Committee (TQEH & LMH)	28 Woodville Road<br>5011<br>Woodville South<br>SA	Australia
256	Northern Sydney Central Coast Health Coast Human Research Ethics Committee	Locked Bag 2915<br>Central Coast Business Centre<br>2250<br>Gosford<br>NSW	Australia
255	Northern Sydney Central Coast Health Coast Human Research Ethics Committee	Locked Bag 2915<br>Central Coast Business Centre<br>2250<br>Gosford<br>NSW	Australia
101	Hôpital Erasme - Comité d'Ethique	Route de Lennik 808<br>1070<br>Bruxelles	Belgium
102	Heilig-Hartkliniek - Ethisch comité	Moeie 18<br>Campus Eeklo<br>9900<br>Eeklo	Belgium
104	Mariaziekenhuis Noord-Limburg VZW - Ethisch comité	Maesensveld 1<br>3900<br>Overpelt	Belgium
105	AZ Sint-Jan AV - Ethisch comité	Ruddershove 10	Belgium

Center No.	Ethics Committee or Institutional Review Board	Address	Country
		8000<br>Brugge	
107	UZ Leuven - Ethisch comité	Herestraat 49<br>UZ Gasthuisberg<br>3000<br>Leuven	Belgium
108	Regionaal Ziekenhuis Sint-Trudo VZW - Ethisch comité	Diestersteenweg 100<br>Campus Sint-Jozef<br>3800<br>Sint-Truiden	Belgium
109	CHU de Charleroi - Hôpital André Vésale - Comité d'éthique médicale de l'ISPPC	Rue de Gozée 706<br>Hôpital André Vésale<br>6110<br>Montigny-le-Tilleul	Belgium
202	Capital Health Research Ethics Board	5790 University Avenue<br>Room 118<br>Center for Clinical Research<br>B3H 1V7<br>Halifax<br>Nova Scotia	Canada
201	Institutional Review Board Services	372 Hollandview Trail<br>Suite 300<br>L4G 0A5<br>Aurora<br>Ontario<br>**and**<br>14845-6 Yonge Street<br>Suite 328<br>L4G 6H8<br>Aurora<br>Ontario	Canada
204	Queen's University Health Services and Affiliated Teaching Hospitals Reseach Ethics Board	Queen's University<br>Office of Research Services<br>Room 307<br>Fleming Hall - Jemmett Wing<br>K7L 3N6<br>Kingston<br>Ontario	Canada
203	University of British Columbia Clinical Research Ethics Board	828 West 10th Avenue<br>Office of the UBC Clinical Research Ethics Board<br>Room 210	Canada

Center No.	Ethics Committee or Institutional Review Board	Address	Country
		Research Pavilion<br>V5Z 1L8<br>Vancouver<br>British Columbia	
210	University of Western Ontario Health Sciences Research Ethics Board	The University of Western Ontario<br>Dental Sciences Building<br>Room 00045<br>N6A 5C1<br>London<br>Ontario	Canada
211	Comite d'ethique de la recerche de l'Hopital Maisonneuve-Rosemont	5415 Boulevard de L'Assomption<br>H1T 2M4<br>Montreal<br>Quebec	Canada
206	St. Michael's Hospital Research Ethics Board	2 Queen Street East<br>9th Floor<br>Suite 913<br>M5B-1W8<br>Toronto<br>Ontario	Canada
213	Institutional Review Board Services	372 Hollandview Trail<br>Suite 300<br>L4G 0A5<br>Aurora<br>Ontario	Canada
205	University of Saskatchewan Biomedical Research Ethics Board	117 Science Place<br>Kirk Hall<br>Room 305<br>Ethics Room<br>S7N 5C8<br>Saskatoon<br>Saskatchewan<br>**and**<br>Research Ethics Office<br>University of Saskatchewan<br>Box 5000 RPO University<br>1607-110 Gymnasium Place<br>S7N 4J8<br>Saskatoon	Canada

Center No.	Ethics Committee or Institutional Review Board	Address	Country
		Saskatchewan	
302	Eticka komise pri Pardubicke krajske nemocnici, a.s.	Kyjevska 44<br>532 03<br>Pardubice	Czech Republic
303	Eticka komise Vseobecne fakultni nemocnice v Praze	Na Bojisti 1<br>128 08<br>Praha 2	Czech Republic
310	Eticka komise Fakultni nemocnice Ostrava	17. listopadu 1790<br>708 52<br>Ostrava	Czech Republic
312	Eticka komise NEUROPS s.r.o.	Jiraskova 1389<br>516 01<br>Rychnov nad Kneznou	Czech Republic
301	Eticka komise Fakultni nemocnice Plzen	Dr. Edvarda Benese 13<br>305 99<br>Plzen - Bory	Czech Republic
304	Eticka komise Fakultni nemocnice Olomouc a LF UP v Olomouci	I. P. Pavlova 6<br>775 20<br>Olomouc	Czech Republic
305	Eticka komise Fakultni nemocnice v Motole	V Uvalu 84<br>150 06<br>Praha 5	Czech Republic
306	Eticka komise Vojenske nemocnice Brno	Zabrdovicka 3<br>636 00<br>Brno	Czech Republic
307	Eticka komise Fakultni nemocnice u sv. Anny v Brne	Pekarska 53<br>656 91<br>Brno<br>**and**<br>Vystavni 17/19<br>656 91<br>Brno	Czech Republic
309	Eticka komise Krajske zdravotni, a.s. - Nemocnice Teplice, o.z.	Duchcovska 53<br>415 01<br>Teplice	Czech Republic
453	CPP Ouest V - Hôpital Pontchaillou	2 rue Henri Le Guilloux<br>Pavillon Clémenceau<br>Cedex<br>35033<br>Rennes	France
455	CPP Ouest V - Hôpital Pontchaillou	2 rue Henri Le Guilloux<br>Pavillon Clémenceau	France

Center No.	Ethics Committee or Institutional Review Board	Address	Country
		Cedex<br>35033<br>Rennes	
402	Ethik-Kommission der Medizinischen Fakultät der Ludwig-Maximilians Universität, München	Marchioninistraße 15<br>81377<br>München	Germany
226	Egeszsegugyi Tudomanyos Tanacs Klinikai Farmakologiai Etikai Bizottsaga	Arany János utca 6-8.<br>1051<br>Budapest	Hungary
230	Egeszsegugyi Tudomanyos Tanacs Klinikai Farmakologiai Etikai Bizottsaga	Arany János utca 6-8.<br>1051<br>Budapest	Hungary
229	Egeszsegugyi Tudomanyos Tanacs Klinikai Farmakologiai Etikai Bizottsaga	Arany János utca 6-8.<br>1051<br>Budapest	Hungary
151	Institutional Helsinki Committee, The Chaim Sheba Medical Center	Tel-Hashomer 52621<br>52621<br>Ramat-Gan	Israel
154	Institutional Helsinki Committee, Medical Center 'Ziv' Safed	P.O. Box 1008<br>13100<br>Safed	Israel
153	Institutional Helsinki Committee, Barzilai Medical Center	3 Histadrut Street<br>78278<br>Ashkelon	Israel
152	Institutional Helsinki Committee, The Lady Davis Carmel Medical Center	7 Michal Street<br>34362<br>Haifa<br>**and**<br>2 Horev Street<br>34362<br>Haifa	Israel
657	VUmc METC	De Boelelaan 1118<br>Frontoffice<br>PK 6 Z 202<br>1081 HV<br>Amsterdam	Netherlands
652	VUmc METC	De Boelelaan 1118<br>Frontoffice<br>PK 6 Z 202<br>1081 HV<br>Amsterdam	Netherlands

Center No.	Ethics Committee or Institutional Review Board	Address	Country
651	VUmc METC	De Boelelaan 1118<br>Frontoffice<br>PK 6 Z 202<br>1081 HV<br>Amsterdam	Netherlands
653	VUmc METC	De Boelelaan 1118<br>Frontoffice<br>PK 6 Z 202<br>1081 HV<br>Amsterdam	Netherlands
656	VUmc METC	De Boelelaan 1118<br>Frontoffice<br>PK 6 Z 202<br>1081 HV<br>Amsterdam	Netherlands
654	VUmc METC	De Boelelaan 1118<br>Frontoffice<br>PK 6 Z 202<br>1081 HV<br>Amsterdam	Netherlands
703	Komisja Bioetyczna przy Akademii Medycznej w Poznaniu	Fredry 10<br>61-701<br>Poznan	Poland
701	Komisja Bioetyczna przy Akademii Medycznej w Poznaniu	Fredry 10<br>61-701<br>Poznan	Poland
702	Komisja Bioetyczna przy Akademii Medycznej w Poznaniu	Fredry 10<br>61-701<br>Poznan	Poland
704	Komisja Bioetyczna przy Akademii Medycznej w Poznaniu	Fredry 10<br>61-701<br>Poznan	Poland
705	Komisja Bioetyczna przy Akademii Medycznej w Poznaniu	Fredry 10<br>61-701<br>Poznan	Poland
706	Komisja Bioetyczna przy Akademii Medycznej w Poznaniu	Fredry 10<br>61-701<br>Poznan	Poland
707	Komisja Bioetyczna przy Akademii Medycznej w Poznaniu	Fredry 10<br>61-701<br>Poznan	Poland
708	Komisja Bioetyczna przy Akademii	Fredry 10	Poland

Center No.	Ethics Committee or Institutional Review Board	Address	Country
	Medycznej w Poznaniu	61-701<br>Poznan	
710	Komisja Bioetyczna przy Akademii Medycznej w Poznaniu	Fredry 10<br>61-701<br>Poznan	Poland
711	Komisja Bioetyczna przy Akademii Medycznej w Poznaniu	Fredry 10<br>61-701<br>Poznan	Poland
751	Ethics Committee within Moscow Regional Research Clinical Institute n.a. M.F.Vladimirskiy	Ulitsa Shchepkina, 61/2<br>129110<br>Moscow	Russia
752	Ethics Committee within Russian Medical Military Academy n.a. S.M.Kirov	Ulitsa Academika Lebedeva, 6<br>194044<br>St. Petersburg	Russia
753	Ethics Committee within Education and Research Medical Center of Russian President Administration	Ulitsa Marshala Timoshenko, 15<br>121356<br>Moscow	Russia
754	Ethics Committee within Institute of Human Brain RAN<br>**and**<br>Ethics Committee within Institution of Russian Academy of Science Institute of Human Brain RAS	Ulitsa Academika Pavlova, 9<br>197376<br>St. Petersburg<br>**and**<br>Ulitsa Academika Pavlova, 9<br>197376<br>St. Petersburg	Russia
755	Ethics Committee within Research Medical Complex "Vashe Zdorovie"	Ulitsa Pionerskaya, 14<br>420029<br>Kazan	Russia
756	Ethics Committee within Research Institute of Neurology of RAMS	Volokolamskoye Shosse, 80<br>125367<br>Moscow	Russia
757	Ethics Committee withinClinical Hospital #122 n.a. L.G. Sokolov	Prospect Kultury, 4<br>194291<br>St. Petersburg	Russia
758	Ethics Committee within Association of Medical and Pharmaceutical Higher Educational Establishments	Ulitsa Trubetskaya, 8 stroenie 2<br>119992<br>Moscow	Russia
351	Eticka komisia Martinskej fakultnej nemocnice	Kollarova 2<br>036 59<br>Martin	Slovak Republic
354	Eticka komisia pri Fakultnej nemocnici s	Mickiewiczova 13	Slovak

Center No.	Ethics Committee or Institutional Review Board	Address	Country
	poliklinikou Bratislava, pracovisko Stare Mesto	813 69<br>Bratislava	Republic
355	Eticka komisia pri Nemocnici s poliklinikou Zilina	Vojtecha Spanyola 43<br>012 07<br>Zilina	Slovak Republic
951	South African Medical Association Research Ethics Committee (SAMAREC)	Nossob Street<br>Block F, Castle Walk Corporate Park<br>Erasmuskloof Ext 3<br>153<br>Pretoria<br>Gauteng	South Africa
952	South African Medical Association Research Ethics Committee (SAMAREC)	Nossob Street<br>Block F, Castle Walk Corporate Park<br>Erasmuskloof Ext 3<br>153<br>Pretoria<br>Gauteng	South Africa
953	University of Cape Town Faculty of Health Sciences Research Ethics Committee	Room E52, Number 24 Old Main Building, Groote Schuur Hospital<br>7925<br>Cape Town<br>Western Cape	South Africa
852	SPUK für Spezialfächer	Lenggstrasse 31<br>UniversitätsSpital Zürich<br>8032<br>Zürich	Switzerland
851	Ethikkommission beider Basel (EKBB)	Hebelstraße 53<br>4056<br>Basel	Switzerland
853	Commission d'Ethique de la Recherche clinique de la Faculté de Biologie et de Médecine	Rue du Bugnon 21<br>Faculté de Médecine<br>Secrétariat central<br>1005<br>Lausanne	Switzerland
601	Institutional Ethics Committee, Istanbul University Medical Faculty	122 Millet Street<br>Neurology Department<br>34390<br>Istanbul	Turkey
602	Institutional Ethics Committee, Dokuz Eylul University Medical Faculty	Mithatpasa Street<br>35340	Turkey

Center No.	Ethics Committee or Institutional Review Board	Address	Country
		Izmir	
603	Institutional Ethics Committee, Ege University Medical Faculty	University Street<br>35100<br>Izmir	Turkey
604	Institutional Ethics Committee, Istanbul University Cerrahpasa Medical Faculty	181 Cerrahpasa Street<br>34098<br>Istanbul	Turkey
605	Institutional Ethics Committee, Uludag University Medical Faculty	Gorukle<br>Office of the Dean<br>Ethice Committee<br>16059<br>Bursa	Turkey
606	Institutional Ethics Committee, Ministry of Health Izmir Tepecik Training and Research Hospital	Gaziler Street<br>Personnel Service<br>Ethics Committee<br>Yenisehir<br>35000<br>Izmir	Turkey
607	Institutional Ethics Committee, Hacettepe University Medical Faculty	Ahmet Adnan Saygun Main Street<br>Sihhiye<br>6100<br>Ankara	Turkey
608	Institutional Ethics Committee, Gazi University Medical Faculty	Bahriye Ucok Main Street<br>6500<br>Ankara	Turkey
609	Institutional Ethics Committee, Bakirkoy Hospital for Psychiatric and Nervous Diseases	Office of the Medical Director<br>Bakirkoy<br>34147<br>Istanbul	Turkey
610	Institutional Ethics Committee, Ministry of Health Goztepe Training and Research Hospital	Office of the Medical Director<br>Goztepe<br>Office of the Medical Director<br>Ethics Committee<br>34722<br>Istanbul	Turkey
611	Institutional Ethics Committee, Gaziantep University Medical Faculty	University Boulevard<br>27310<br>Gaziantep	Turkey
612	Institutional Ethics Committee, Mersin University Medical Faculty	Ciftlikkoy Campus<br>33169<br>Mersin	Turkey

Center No.	Ethics Committee or Institutional Review Board	Address	Country
901	Oxfordshire Research Ethics Committee B	Granville Way<br>Astral House<br>2nd Floor<br>Chaucer Business Park<br>OX26 4JT<br>Bicester	United Kingdom
903	Oxfordshire Research Ethics Committee B	Granville Way<br>Astral House<br>2nd Floor<br>Chaucer Business Park<br>OX26 4JT<br>Bicester	United Kingdom
904	Oxfordshire Research Ethics Committee B	Granville Way<br>Astral House<br>2nd Floor<br>Chaucer Business Park<br>OX26 4JT<br>Bicester	United Kingdom
906	Oxfordshire Research Ethics Committee B	Granville Way<br>Astral House<br>2nd Floor<br>Chaucer Business Park<br>OX26 4JT<br>Bicester	United Kingdom
908	Oxfordshire Research Ethics Committee B	Granville Way<br>Astral House<br>2nd Floor<br>Chaucer Business Park<br>OX26 4JT<br>Bicester	United Kingdom
909	Oxfordshire Research Ethics Committee B	Granville Way<br>Astral House<br>2nd Floor<br>Chaucer Business Park<br>OX26 4JT<br>Bicester	United Kingdom

FREEDOMS II (FTY720D 2309)

Independent Ethics Committees or Institutional Review Boards by study center

Center No.	Ethics Committee or Institutional Review Board	Department / Organization	Address Country
102	The Ottawa Hospital Research Ethics Board		Ottawa ON K1H 8L6 Canada
103	Institutional Review Board Services		Aurora Ontario L4G 0A5 Canada
153	Ethik-Kommission Der Medizinischen Universitat Wien Und Des		Wien 1090 Austria
201	Istanbul University Istanbul Medical Faculty Ethics Committee		İstanbul Turkey
202	Dokuz Eylul University Istanbul Medical Faculty Ethics Committee		İzmir Turkey
203	Istanbul University Cerrahpasa Medical Faculty Ethics Committee		İstanbul Turkey
204	S.B. Izmir Tepecik Training and Research Hospital Ethics Committee		İzmir Turkey
205	Bakirkoy Psychiatric Hospital Ethics Committee		İstanbul Turkey
301	Komisja Biotyczna przy OIL		Białystok 15-082 Poland
303	Komisja Biotyczna przy OIL		Białystok 15-082 Poland
304	Komisja Biotyczna przy OIL		Białystok 15-082 Poland
401	Comisia Nationala de Etica pentru Studiul Clinic al Medicamentului		48 Aviator Sanatescu street Bucharest 011478 Romania
402	Comisia Nationala de Etica pentru Studiul Clinic al Medicamentului		48 Aviator Sanatescu street Bucharest 011478 Romania
404	Comisia Nationala de Etica pentru Studiul Clinic al Medicamentului		48 Aviator Sanatescu street Bucharest 011478 Romania
501	Wake Forest University Health Science	Office of Reasearch	Winston-Salem NC 27157

Center No.	Ethics Committee or Institutional Review Board	Department / Organization	Address Country
502	Western, Institutional Review Board		Olympia WA 98502
503	Quorom Review Inc.		Seattle WA 98101
504	University of Maryland, Baltimore IRB	Human Research Protection Office	Baltimore MD 21201
505	Aurora IRB		Milwaukee WI 53201-0342
507	Quorum Review Inc.		Seattle WA 98101
508	Quorum Review, Inc		Seattle WA 98101
510	Chesapeake Research Review Inc.		Columbia MD 21046-3403
510	Human Assurance Committee	Medical College of Georgia	Augusta GA 30912
511	Quorum Review Inc.		Seattle WA 98101
512	Quorum Review Inc.		Seattle WA 98101
513	Vanderbilt University Institutional Review Board		Nashville TN 37232-4315
514	The Committee for the Protection of Human Subjects		Houston TX 77030<br>United States
515	Committee on Research Involving Human Subjects	Melville Library	Stony Brook NY 11794-3368<br>United States
516	University of Louisville Institutional Review Board	Human Subjects Protection Program Office	Louisville KY 40202<br>United States
517	Quorum Review Inc		Seattle WA 98101<br>United States
518	Quorum Review Inc		Seattle WA 98101<br>United States
519	Western Institutional Review Board		Olympia WA 98502-5810<br>United States
519	Human Subjects research office		Miami FL, 33136<br>United States
520	Rush University Medical Center	Institutional Review Board	Chicago IL 60612<br>United States
521	IRB/OSA		Philadelphia PA 19107<br>United States
522	Henry Ford Health System	Research Administration	Detroit MI 48202<br>United States

Center No.	Ethics Committee or Institutional Review Board	Department / Organization	Address Country
523	Office of Regulatory Affairs		Philadelphia PA 19104 United States
524	St. Joseph's Hospital and Medical Center	Institutional Review Board for Human Research	Phoenix AZ 85013 United States
525	University of New Mexico Health Sciences Center	Human Research Review Committee	Albuquerque NM 87131 United States
526	IUPUI Institutional Review Board		Indianapolis IN 46202 United States
528	Office of Research and Administration	Sponsored Projects	Irvine CA 92697-7600 United States
529	Alta Bates Summit Medical Center Institutional Review Board		Berkeley CA 94705 United States
531	Mount Sinai School of Medicine	Institutional Review Board	NY NY 10029 United States
533	Quorum Review Inc		Seattle WA 98101United States
535	University of Vermont Research Protections Office	Office of Sponsored Programs	Colchester VT 05446 United States
536	Western Institutional Review Board		Olympia WA 98502-9810 United States
536	Quorum Review		Seattle WA 98101 United States
537	Mercy Medical Center-Des Moines	Institutional Review Committee	Des Moines IA 50314 United States
538	Quorum Review Inc		Seattle WA 98101 United States
539	Western Institutional Review Board		Olympia WA 98502-9810 United States
541	Michigan State University Office of regulatory affairs	Human Research Protection Programs	East Lansing MI 48825 United States
543	Northwestern University	Institutional Review Board	Chicago IL 60611 United States
545	University of Kansas Medical Center	Human Subjects Committee	Kansas City KS 66160 United States
546	Western Institutional Review Board		Olympia WA 98502-9810 United States
547	University of Chicago Medical Center	Institutional Review Board	Chicago IL 60637 United States
548	Weill Medical College of Cornell University	Committee on Human Rights and Research Institutional Review Board	NY NY 10021 United States
549	Western Institutional Review Board		Olympia WA 98502-9810 United States

Center No.	Ethics Committee or Institutional Review Board	Department / Organization	Address Country
550	Quorum Review Inc		Seattle WA 98101<br>United States
551	Quorum Review Inc		Seattle WA 98101<br>United States
552	University of Wisconsin	Health Schiences Institutional Review Boards	Madison WI 53705<br>United States
553	Quorum Review Inc		Seattle WA 98101<br>United States
554	Biomedical Research Alliance of NY, LLC	IRB	Great Neck NY 11021<br>United States
555	Western Institutional Review Board		Olympia WA 98502-9810<br>United States
556	Quorum Review Inc		Seattle WA 98101<br>United States
557	Quorum Review Inc		Seattle WA 98101United States
558	Quorum Review Inc		Seattle WA 98101<br>United States
559	UC Davis Office of Research	IRB Administration	Sacramento CA 95817<br>United States
561	Committee on Human Research	Office Research	San Francisco CA 94118<br>United States
563	Methodist Hospital IRB	Arcadia	CA 91066-6016
565	Western Institutional Review Board		Olympia WA 98502-5010<br>United States
566	Human Investigation Committee	Yale University School of Med	New Haven CT 06520<br>United States
567	Mecry Health System's Review Board		Oklahoma City 73120
569	Western Institutional Review Board		Olympia WA 98502<br>United States
570	Dukle University Health System IRB		Durham NC 27705<br>United States
572	IRBMED		Ann Arbor MI 48103-4943<br>United States
574	Quorum Review Inc		Seattle WA 98101<br>United States
575	Quorum Review Inc		Seattle WA 98101<br>United States
576	University of South Alabama	IRB	Mobile AL<br>United States
577	SUNY - Upstate Medical University	IRB for the Protection of Human Subjects	Syracuse NY<br>United States
578	Quorum Review Inc.		Seattle WA 98101<br>United States

Center No.	Ethics Committee or Institutional Review Board	Department / Organization	Address Country
579	Quorum Review Inc.		Seattle WA 98101 United States
580	Quorum Review Inc.		Seattle WA 98101 United States
581	Western Institutional Review Board		Olympia WA 98502 United States
583	IRB for Health Sciences Research	University of Virginia	Charlottesville VA 22908 United States
584	Georgetown University IRB		Washington DC United States
585	Quorum Review Inc		Seattle WA 98101United States
586	Committee for the Protection of Human Subjects	Office of Sponsored Projects	Hanover NH 03755 United States
587	Western Institutional Review Board		Olympia WA 98502 United States
588	Quorum Review		Seattle WA 98101 United States
589	Quorum Reveiw Inc		Seattle WA 98101 United States
590	Quorum Review Inc		Seattle WA 98101 United States
591	Renown Regional Medical Center IRB		Reno NV 89502 United States
592	Johns Hopkins IRB	Office of Human Subjects Research	Baltimore MD 21205-1911 United States
593	Human Investigation Committee	Wayne State University	Detroit MI 48201 United States
594	Office of Human Research Ethics, Biomedical IRB	Universtiy of North Carolina, Chapel Hill	Chapel Hill NC 27599 United States
595	Quorum Review Inc		Seattle WA 74137 United States
597	Quorum Review Inc		Seattle WA 98101 United States
598	Quorum Review Inc		Seattle WA 98101 United States
599	Quorum Review Inc		Seattle WA 98101 United States
600	Southwestern Vermont Healtcare IRB		Bennington VT 05201 United States
601	Quorum Review Inc		Seattle WA 98101 United States
602	Alexan Brothers Hospital Network IRB		Arlington Heights IL 60005 United States

Center No.	Ethics Committee or Institutional Review Board	Department / Organization	Address Country
603	Quorum Review Inc		Seattle WA 98101 United States
604	Quorum Review Inc		Seattle WA 98101 United States
605	Legacy IRB	Legacy Clinical Research and Technology Center	Portland OR 97232 United States
606	Quorum Review Inc.		Seattle WA 98101 United States
607	Saint Luke's Hospital IRB		Kansas City MO United States
608	Quroum Review Inc		Seattle WA 98101 United States
609	Cedars Sinai Medical Center IRB		Los Angeles CA United States
610	West Virginia University	Office of Research Compliance	Morgantown MV 26506 United States
612	Quorum Review Inc		Seattle WA 98101 United States
615	Allegheny General Hospital IRB		Pittsburgh PA United States
616	Quorum Review Inc		Seattle WA 98101 United States
618	Caritas St. Elizabeth's Medical Center IRB		Boston MA 02135 United States
619	Human Research and Investigation Committee		Newton MA 02462 United States
620	University of MA medical School IRB		Worcester MA 01655 United States
621	Dean IRB		Middletown WI 53562 United States
626	Quorum Review Inc.		Seattle WA 98101 United States
701	Northern Sydney Central Coast Human Research Ethics Committee	Level 2, Building 51, Royal North Shore Hospital, Pacific Highway, St Leonards	Sydney NSW 2065 Australia
901	Frenchay Research Ethics Committee	Southmead Hospital	Bristol BS10 5NB United Kingdom

INFORMS (FTY720D 2306)

Independent Ethics Committees or Institutional Review Boards by study center

Center No.	Ethics Committee or Institutional Review Board	Department / Organization	Address Country
0102	National Multiple Sclerose Centrum v.z.w.	Ethische Commissie	Melsbroek 1820 Belgium
0103	UZ Leuven - Campus Gasthuisberg	Commissie Medische Ethiek - Toetsingscommissie	Leuven 3000 Belgium
0104	CHU de Charleroi - Hôpital André Vésale	Comité d’Ethique I.S.P.P.C.	Montigny-le-Tilleul 6110 Belgium
0106	UZ Antwerpen	Commissie Medische Ethiek	Edegem 2650 Belgium
0107	CHU Liège	Comité d'Ethique Hospitalo-Facultaire Universitaire de Liège	Liège 4000 Belgium
0121	MNI Research Ethics	Montreal Neurological Institute	Montreal Quebec H3A 2B4 Canada
0122	Capital Health Research Ethics Board	Centre for clinical research	Halifax NS B3H 1V7 Canada
0124	The University of British Columbia	Office of Research (Services) Ethics	Vancouver BC V5Z 1L8 Canada
0125	Ottawa Hospital Research Ethics Board		Ottawa ON K1Y 4E9 Canada
0126	Ontario IRB		Aurora Ontario L4G A05 Canada
0127	St. Michael's Hospital Research Ethics Office		Toronto ON M5B 1W8 Canada
0128	Fraser Health Research Ethics Board		Surrey BC V3R 7P8 Canada
0129	Conjoint Health Research Ethics Board		Calgary AB T2N 4N1 Canada

Center No.	Ethics Committee or Institutional Review Board	Department / Organization	Address Country
0130	University of Saskatchewan Biomedical Research Ethics Board		Saskatoon SK S7N 4J8<br>Canada
0131	Committe of Ethics for Research		Montreal Quebec H9X 0A9<br>Canada
0132	Health Research Ethics Board (Biomedical Panel)		Edmonton AB AB T6G 1K8<br>Canada
0133	Institutional Review Board Services		Aurora Ontario L4G 0A5<br>Canada
0135	Sunnybrook Health Sciences Centre REB		Toronto ON M4N 3M5<br>Canada
0161	Eticka Komise	Fakultní nemocnice Plzeň, E. Beneše 13, 305 99 Plzeň	Plzeň Plzeň<br>Czech Republic
0162	Eticka Komise	Všeobecná fakultní nemocnice v Praze<br>Na Bojišti , 128 08 Praha 2	Praha Praha 12808<br>Czech Republic
0166	Eticka Komise	Fakultní nemocnice u svaté Anny<br>Pekařská 53<br>656 91 Brno	Brno Brno 65691<br>Czech Republic
0168	Eticka Komise	Nemocnice Teplice<br>Duchovská 53<br>415 29 Teplice	Teplice Teplice 41529<br>Czech Republic
0169	Eticka Komise	Fakultní nemocnice Ostrava Poruba<br>17.listopadu 1790<br>Ostrava 70853	Ostrava Ostrava 70853<br>Czech Republic
0170	Eticka Komise	Fakultní nemocnice Hradec Králové, Sokolská 581, 500 05 Hradec Králové	Hradec Kralové Hradec Králové 500 05<br>Czech Republic
0201	HUS Tutkimuseettiset toimikunnat	Medisiininen eettinen toimikunta	Helsinki 00029<br>Finland
0202	HUS Tutkimuseettiset toimikunnat	Medisiininen eettinen toimikunta	Helsinki 00029<br>Finland
0203	HUS Tutkimuseettiset toimikunnat	Medisiininen eettinen toimikunta	Helsinki 00029<br>Finland
0221	CPP Ile-de-France VI	Groupe Hospitalier Pitié-Salpêtrière<br>4 Pavillon de la Force	PARIS 75651<br>France
0222	CPP Ile-de-France VI	Groupe Hospitalier Pitié-Salpêtrière<br>4 Pavillon de la Force	PARIS 75651<br>France

Center No.	Ethics Committee or Institutional Review Board	Department / Organization	Address Country
0223	CPP Ile-de-France VI	Groupe Hospitalier Pitié-Salpêtrière 4 Pavillon de la Force	PARIS 75651 France
0224	CPP Ile-de-France VI	Groupe Hospitalier Pitié-Salpêtrière 4 Pavillon de la Force	PARIS 75651 France
0225	CPP Ile-de-France VI	Groupe Hospitalier Pitié-Salpêtrière 4 Pavillon de la Force	PARIS 75651 France
0226	CPP Ile-de-France VI	Groupe Hospitalier Pitié-Salpêtrière 4 Pavillon de la Force	PARIS 75651 France
0227	CPP Ile-de-France VI	Groupe Hospitalier Pitié-Salpêtrière 4 Pavillon de la Force	PARIS 75651 France
0228	CPP Ile-de-France VI	Groupe Hospitalier Pitié-Salpêtrière 4 Pavillon de la Force	PARIS 75651 France
0251	Ethik-Kommission	Julius-Maximilians-Universtität Würzburg / Medizinische Fakultät, Institut für Pharmakologie und Toxikologie	Wurzburg na Germany
0252	Ethik-Kommission	Landesamt für Gesundheit und Soziales des Landes Berlin	Berlin na Germany
0253	Ethik-Kommission	Friedrich-Alexander-Universität Erlangen-Nürnberg / Medizinische Fakultät	Erlangen na Germany
0254	Ethik-Kommission	Medizinische Fakultät der Ludwig-Maximilians-Universität	München na Germany
0255	Ethik-Kommission	Universitätsklinikum Düsseldorf	Düsseldorf na Germany
0256	Ethik-Kommission	Albert-Ludwigs-Universität Freiburg	Freiburg na Germany
0257	Ethik-Kommission	Landesärztekammer Brandenburg	Cottbus na Germany
0258	Ethik-Kommission	Landesärztekammer Brandenburg	Cottbus na Germany
0259	Ethik-Kommission	Landesamt für Gesundheit und Soziales des Landes Berlin	Berlin na Germany
0261	Geschäftsstelle der Ethik-Kommission	Technische Universität Dresden / Medizinische Fakultät "Carl Gustav Carus"	Dresden na Germany
0262	Ethik-Kommission	Medizinische Fakultät der Technischen Universität München / Klinikum rechts der Isar	München na Germany

Center No.	Ethics Committee or Institutional Review Board	Department / Organization	Address Country
0263	Ethik-Kommission	Landesärztekammer Rheinland-Pfalz	Mainz na<br>Germany
0264	Ethik-Kommission	Ärztekammer Westfalen-Lippe und der Medizinischen Fakultät der WWU-Münster	Münster na<br>Germany
0265	Ethik-Kommission	Otto-von-Guericke-Universität Magdeburg<br>Medizinische Fakultät	Magdeburg na 39120<br>Germany
0266	Ethik-Kommission	Universitätsklinikum Essen<br>Medizinische Fakultät der Universität Duisburg-Essen	Essen na<br>Germany
0266	Ethik-Kommission	Universitätsklinikum Essen<br>Medizinische Fakultät der Universität Duisburg-Essen	Essen na<br>Germany
0267	Ethik-Kommission	Ruhr-Universität Bochum<br>Medizinische Fakultät	Bochum na<br>Germany
0267	Ethik-Kommission	Ruhr-Universität Bochum<br>Medizinische Fakultät	Bochum na<br>Germany
0268	Ethik-Kommission	Medizinische Hochschule Hannover	Hannover na<br>Germany
0269	Ethik-Kommission	Landesärztekammer Rheinland-Pfalz	Mainz na<br>Germany
0301	Comitato Etico	COMITATO ETICO DELL'IRCCS FONDAZIONE SAN RAFFAELE DEL MONTE TABOR DI MILANO- Via Olgettina 60	Milano 20132<br>Italy
0303	Comitato Etico Idipendente Locale	COMITATO ETICO DELL`AZIENDA OSPEDALIERA POLICLINICO CONSORZIALE DI BARI<br>Piazza Giulio Cesare, 11	Bari 70124<br>Italy
0304	Comitato de Etica	COMITATO ETICO DELL'UNIVERSITA' DEGLI STUDI GABRIELE D'ANNUNZIO E<br>DELLA ASL 2 LANCIANO-VASTO-CHIETI di CHIETI<br>VIA DEI VESTINI, 31	Chieti 66100<br>Italy
0305	Comitato de Etica	COMITATO ETICO DELL'AZIENDA OSPEDALIERA S.ANTONIO ABATE DI GALLARATE<br>LARGO BOITO 2	Gallarate 21013<br>Italy

Center No.	Ethics Committee or Institutional Review Board	Department / Organization	Address Country
0306	Comitato de Etica Aziendale	COMITATO ETICO DELL'AZIENDA OSPEDALIERA UNIVERSITARIA SAN MARTINO DI GENOVA Largo Benzi Rosanna, 10	Genova 16132 Italy
0307	Comitato de Etica Aziendale	COMITATO ETICO AZIENDA OSPEDALIERA SPEDALI CIVILI DI BRESCIA PIAZZALE SPEDALI CIVILI, 1	Brescia 25123 Italy
0308	Comitato de Etica Sperimentazione	COMITATO ETICO PER LA SPERIMENTAZIONE DELL'AZIENDA OSPEDALIERA DI PADOVA - VENETO VIA NICOLO' GIUSTINIANI, 2	Padova 35128 Italy
0309	Ospedale Maggiore Policlinico	COMITATO ETICO DELLA FONDAZIONE IRCCS CA' GRANDA OSPEDALE MAGGIORE POLICLINICO - PALAZZO UFFICI - 2° piano - c.a. dr.ssa Patti VIA FRANCESCO SFORZA, 28	Milano 20122 Italy
0310	Comitato de Etica Indipendiente	COMITATO ETICO INDIPENDENTE presso la FONDAZIONE PTV POLICLINICO TOR VERGATA DI ROMA VIALE OXFORD, 81	Roma 00133 Italy
0311	Comitato de Etica Indipendiente	COMITATO ETICO DELL'AZIENDA OSPEDALIERA SANT'ANDREA DI ROMA VIA DI GROTTAROSSA, 1035-1039	Roma 00189 Italy
0312	Comitato Etico	COMITATO ETICO DELL'IRCCS FONDAZIONE ISTITUTO NEUROLOGICO CASIMIRO MONDINO DI PAVIA VIA C. MONDINO, 2	Pavia 27100 Italy
0313	Comitato Etico	POLICLINICO-VITTORIO EMANUELE DI CATANIA per il POU Policlinico G. Rodolico dell'Università di Catania VIA SANTA SOFIA, 78	Catania 95123 Italy

Center No.	Ethics Committee or Institutional Review Board	Department / Organization	Address Country
0314	Comitato Etico	COMITATO ETICO DELLA FONDAZIONE ISTITUTO S. RAFFAELE GIUSEPPE GIGLIO DI CEFALU' Contrada Pietropollastra - Pisciotto	Cefalù 90015 Italy
0315	Comitato Etico	COMITATO ETICO DELL´AZIENDA OSPEDALIERA UNIVERSITARIA S. LUIGI GONZAGA DI ORBASSANO Via Regione Gonzole, 10	Orbassano 10043 Italy
0361	METC VUmc	N/A	Amsterdam N/A 1007 MB Netherlands
0361	METC VUmc	N/A	Amsterdam N/A 1007 MB Netherlands
0362	METC VUmc	N/A	Amsterdam N/A 1007 MB Netherlands
0363	METC VUmc	N/A	Amsterdam N/A 1007 MB Netherlands
0364	METC VUmc	N/A	Amsterdam N/A 1007 MB Netherlands
0366	METC VUmc	N/A	Amsterdam N/A 1007 MB Netherlands
0367	METC VUmc	N/A	Amsterdam N/A 1007 MB Netherlands
0401	CEIC HOSPITAL UNIV. VALL D’HEBRON		BARCELONA 08035 Spain
0402	CEIC Hospital Univ. Germans Trias i Pujol		BADALONA Barcelona 08915 Spain
0403	CEIC Hospital de Girona “Dr. Josep Trueta”		GIRONA GIRONA 17007 Spain
0404	CEIC Hospital Univ. Virgen de la Macarena Comité Autonómico Andalucía (CAEC)	COMITÉ COORDINADOR DE ETICA EN INVESTIGACION BIOMEDICA EN ANDALUCIA Consejeria de Salud	SEVILLA Sevilla 41092 41020 Spain

Center No.	Ethics Committee or Institutional Review Board	Department / Organization	Address Country
0405	CEIC Área 7 - Hospital Clínico San Carlos de Madrid		MADRID MADRID 28040 Spain
0406	CEIC Hospital Universitario La Fe de Valencia	Secretaría del CEIC (Comité Ético de Investigación Clínica	Valencia Valencia 46026 Spain
0407	CEIC Área 6 - Hospital Universitario Puerta de Hierro Majadahonda	Secretaría Técnica del CEIC	MAJADAHONDA MADRID 28222 Spain
0408	CEIC Hospital de Basurto<br>CEIC Euskadi	Secretaría del Comité Ético de Investigación Clínica<br>Dirección de Farmacia<br>Dep. Sanidad Gobierno Vasco	VITORIA BILBAO ALAVA VIZCAYA 01010 48013 Spain
0409	CEIC Hospital de Lleida “Arnau de Vilanova”		LLEIDA Lleida 25198 Spain
0411	CEIC Hospital Universitari de Bellvitge	Secretaria administrativa	L'Hospitalet de Llobregat Barcelona 08907 Spain
0412	CEIC Área 5 - Hospital Universitario La Paz	Secretaría Técnica del Comité Ético de Investigación Clínica	MADRID MADRID 28046 Spain
0413	CEIC Hospital Clínic i Provincial	Agencia de Ensayos Clínicos<br>Servicio de Farmacia	BARCELONA BARCELONA 08036 Spain
0441	Regionala etikprövningsnämnden i Stockhlom<br>FE 289		Stockholm<br>Sweden
0442	Regionala etikprövningsnämnden i Stockhlom<br>FE 289		Stockholm<br>sweden
0461	Ethikkommission Nordwest- und Zentralschweiz (EKNZ)		Basel<br>Switzerland
0462	Kantonale Ethikkommission Zürich		Zurich<br>Switzerland
0462	Kantonale Ethikkommission Zürich		Zurich<br>Switzerland
0463	Commission cantonale d'éthique de la recherche sur l'être humain		Lausanne<br>Switzerland

Center No.	Ethics Committee or Institutional Review Board	Department / Organization	Address Country
0464	Kantonale Ethikkommission Bern		Bern Switzerland
0465	Comitato etico cantonale c/o Ufficio di sanità		Bellinzona Switzerland
0481	Hacettepe University Tıp Fakültesi Dekanlığı		Ankara Turkey
0482	Hacettepe University Tıp Fakültesi Dekanlığı		Ankara Turkey
0483	Hacettepe University Tıp Fakültesi Dekanlığı		Ankara Turkey
0484	Hacettepe University Tıp Fakültesi Dekanlığı		Ankara Turkey
0485	Hacettepe University Tıp Fakültesi Dekanlığı		Ankara Turkey
0502	Quorum Review, Inc.		Seattle WA. USA
0503	Committie for Protection of Human Subjects		Houston TX USA
0504	University of Maryland School of Medicine	Human Research Protections Office	Baltimore MD USA
0505	Johns Hopkins Institutional Review Board	Office of Human Subjects Research	Baltimore MD 21205-1911 USA
0506	Partners Human Research Committee		Boston MA. USA
0507	UTSW Institutional Review Board		Dallas TX 75390-8843 USA
0508	Mount Sinai School of Medicine	Institutional Review Board	New York NY 10029-6754 USA
0509	UC Davis Office of Research	IRB Administration	Sacramento CA USA
0510	Wetern Institutional Review Board		Olympia WA USA
0511	Cleveland Clinic Institutional Review Board WB-2		Cleveland Ohio USA
0512	Human Subjects Research Office		Miami Florida USA
0514	Human Subjects Committee	University of Kansas Medical Center	Kansas City Kansas USA
0515	Office of Research Services		Chicago Il. USA

Center No.	Ethics Committee or Institutional Review Board	Department / Organization	Address Country
0516	Quorum Review, Inc.		Seattle WA USA
0517	Office of Protection of Research Subjects	Institutional Review Board	Chicago IL. USA
0518	Committee on Research Involving Human Subjects (CORIHS)		Stony Brook NY 11794-3368 USA
0519	Western Institutional Review Board (WIRB)		Olympia WA 98502-5010 USA
0520	Institutional Review Board for health Sciences Research		Charlottesville VA. USA
0521	Copernicus Group Institutional Review Board		Resaerch Triangle Park NC. USA
0522	Human Investigation Committee	Wayne State University	Detroit MI USA
0523	Human research Protection Office	Washington University School of Medicine	St. Louis M0. USA
0524	Quorum Review ,Inc.		Seattle WA. USA
0528	Research/Human Subjects Committee	Caritas St. Elizabeth's Medical Center	Boston MA. USA
0529	Quorum Review, Inc.		Seattle WA. USA
0530	Western Institutional Review Board		Olympia WA. USA
0531	Quorum Review, Inc.		Seattle WA. USA
0532	Western Institutional Review Board		Olympia WA. 98508-2029 USA
0533	WIRB		Olympia WA. USA
0534	University of Vermont Research Protections Office	Office of Sponsored Programs	Colchester VT. USA
0535	Quorum Review. Inc.		Seattle WA. USA
0536	Western Institutioanl Review Board		Olmypia Wa. 98502-5010 USA

Center No.	Ethics Committee or Institutional Review Board	Department / Organization	Address Country
0537	Duke University Health System Institutional Review Board		Durham NC<br>USA
0538	Quorum Review, Inc.		Setaale WA.<br>USA
0539	Vanderbilt University Institutional Review Board		Nashville TN. 37232-4315<br>USA
0601	NRES Committee: London – South East Bristol REC Centre	Level 3 Block B<br>Whitefriars<br>Lewins Mead	Bristol BS1 2NT<br>United Kingdom
0602	NRES Committee: London – South East Bristol REC Centre	Level 3 Block B<br>Whitefriars<br>Lewins Mead	Bristol BS1 2NT<br>United Kingdom
0603	NRES Committee: London – South East Bristol REC Centre	Level 3 Block B<br>Whitefriars<br>Lewins Mead	Bristol BS1 2NT<br>United Kingdom
0604	NRES Committee: London – South East Bristol REC Centre	Level 3 Block B<br>Whitefriars<br>Lewins Mead	Bristol BS1 2NT<br>United Kingdom
0606	NRES Committee: London – South East Bristol REC Centre	Level 3 Block B<br>Whitefriars<br>Lewins Mead	Bristol BS1 2NT<br>United Kingdom
0607	NRES Committee: London – South East Bristol REC Centre	Level 3 Block B<br>Whitefriars<br>Lewins Mead	Bristol BS1 2NT<br>United Kingdom
0608	NRES Committee: London – South East Bristol REC Centre	Level 3 Block B<br>Whitefriars<br>Lewins Mead	Bristol BS1 2NT<br>United Kingdom
0609	NRES Committee: London – South East Bristol REC Centre	Level 3 Block B<br>Whitefriars<br>Lewins Mead	Bristol BS1 2NT<br>United Kingdom
0610	NRES Committee: London – South East Bristol REC Centre	Level 3 Block B<br>Whitefriars<br>Lewins Mead	Bristol BS1 2NT<br>United Kingdom
0621	SSWAHS Ethics Review Committee	Royal Prince Alfred Hospital Zone	Camperdown NSW<br>Australia
0622	SSWAHS Ethics Review Committee	Royal Prince Alfred Hospital Zone	Camperdown NSW<br>Australia
0623	Melbourne Health Human Research Ethics Committee	N/A	Parkville VIC<br>Australia
0624	Eastern Health Research and Ethics Committee	N/A	Boxhill VIC<br>Australia

Center No.	Ethics Committee or Institutional Review Board	Department / Organization	Address Country
0625	Austin Health Human Research Ethics Committee	N/A	Heidelberg VIC Australia
0626	University of Tasmania Human Research Ethics Committee	N/A	Hobart Tasmania Australia
0641	Medical Research Council	Ethics Committee for Clinical Pharmacology	Budapest NA Hungary
0642	Medical Research Council	Ethics Committee for Clinical Pharmacology	Budapest NA Hungary
0643	Medical Research Council	Ethics Committee for Clinical Pharmacology	Budapest NA Hungary
0644	Medical Research Council	Ethics Committee for Clinical Pharmacology	Budapest NA Hungary
0645	Medical Research Council	Ethics Committee for Clinical Pharmacology	Budapest NA Hungary
0645	Medical Research Council	Ethics Committee for Clinical Pharmacology	Budapest NA Hungary
0646	Medical Research Council	Ethics Committee for Clinical Pharmacology	Budapest NA Hungary
0702	Den Videnskabestiske Komité For Region Hovedstaden		Kongens Vænge 2 Denmark
0703	Den Videnskabestiske Komité For Region Hovedstaden		Kongens Vænge 2 Denmark
0751	Komisja Bioetyki Uniwersytetu Medycznego w Łodzi		Łódź 90-419 Poland
0752	Komisja Bioetyczna przy Instytucie Psychiatrii i Neurologii		Warszawa 02-957 Poland
0753	Komisja Bioetyczna Warszawskiego Uniwersytetu Medycznego w Warszawie		Warszawa 02-091 Poland
0754	Komisja Bioetyczna przy Uniwersytecie Medycznym w Lublinie		Lublin 20-059 Poland

TRANSFORMS (FTY720D 2302)

Independent Ethics Committees or Institutional Review Boards by study center

Center No.	Ethics Committee or Institutional Review Board	Department / Organization	Address Country
0101	CEIC Hospitals Vall dHebron		Barcelona Barcelona 08035 Spain
0102	CEIC Ciutat Sanitària i Universitària de Bellvitge		l'Hospitalet de Llobregat Barcelona 08907 Spain
0103	CEIC Hospital Universitario Carlos Haya		Málaga Málaga 29010 Spain
0104	CEIC Hospital Universitario Virgen Macarena		Sevilla Sevilla 41071 Spain
0105	CEIC Área 7 - Hospital Clínico San Carlos de Madrid		Madrid Madrid 28040 Spain
0106	CEIC Hospital Universitario La Fé		Valencia Valencia 46009 Spain
0107	CEIC Área 6 - Hospital Universitario Puerta de Hierro de Majadahonda		Majadahonda Madrid 28222 Spain
0108	CEIC del Hospital de Basurto		Bilbao Vizcaya 48013 Spain
0121	CPP Sud Méditerranée II	Hôpital Salvator	249 Boulevard Sainte Marguerite MARSEILLE Cédex 09 13274 France
0122	CPP Sud Méditerranée II	Hôpital Salvator	249 Boulevard Sainte Marguerite MARSEILLE Cédex 09 13274 France
0123	CPP Sud Méditerranée II	Hôpital Salvator	249 Boulevard Sainte Marguerite MARSEILLE Cédex 09 13274 France
0124	CPP Sud Méditerranée II	Hôpital Salvator	249 Boulevard Sainte Marguerite MARSEILLE Cédex 09 13274 France
0125	CPP Sud Méditerranée II	Hôpital Salvator	249 Boulevard Sainte Marguerite MARSEILLE Cédex 09 13274 France
0126	CPP Sud Méditerranée II	Hôpital Salvator	249 Boulevard Sainte Marguerite MARSEILLE Cédex 09 13274 France
0141	UCL - St. Luc	Comité d'ethique - Avenue Hippocrate 55.14, Tour Harvey, niveau 0	Bruxelles 1200 Belgium
0142	National Multiple Sclerose Centrum v.z.w.	Ethische Commissie - Vanheylenstraat 16	Melsbroek 1820 Belgium
0144	UZ BRUSSEL	Ethische Commissie - Laarbeeklaan 101	Brussel 1090 Belgium
0145	St. Trudo Ziekenhuis - ethische commissie	Diestersteenweg 100 -	Sint Truiden 3800 Belgium
0201	Comitato Etico dell'IRCCS Fondazione San Raffaele del Monte Tabor di Milano	Fondazione San Raffaele Via Olgettina, 60	Milano Milano 20132 Italy
0202	Comitato Etico dell'Azienda	Azienda Ospedaliera di	Gallarate Varese 21013

Center No.	Ethics Committee or Institutional Review Board	Department / Organization	Address Country
	Ospedaliera S. Antonio Abate di Gallarate	Gallarate, Largo Boito 2	Italy
0203	Comitato Etico dell'Azienda Ospedaliera Universitaria San Martino di Genova	Azienda Ospedaliera Universitaria San Martino, Largo Benzi Rosanna 10	Genova Genova 16132 Italy
0204	Comitato Etico dell' Ospedale S. Andrea di Roma	Ospedale S. Andrea, Via di Grottarossa 1035	Roma Roma 00189 Italy
0205	Comitato Etico dell'Azienda Ospedaliero-Universitaria Ospedali Riuniti Umberto I-GM Lancisi-G-Salesi di Ancona	Azienda Ospedaliero-Universitaria Ospedali Riuniti Umberto I- GM Lancisi-G-Salesi, Via Conca, Torrette	Ancona Ancona 60020 Italy
0206	Comitato Etico dell'IRCCS Fondazione Istituto Neurologico Casimiro Mondino di Pavia	IRCCS Fondazione Istituto Neurologico Casimiro Mondino, Via C. Mondino 2	Pavia Pavia 27100 Italy
0207	Comitato Etico dell'IRCCS Ospedale Maggiore Policlinico Mangiagalli e Regina Elena di Milano	Ospedale Maggiore Policlinico Mangiagalli e Regina Elena, Via F. Sforza, 35	Milano Milano 20122 Italy
0208	Comitato Etico per la Sperimentazione dell'Azienda Ospedaliera di Padova-Veneto	Azienda Ospedaliera di Padova- Veneto, Via Giustiniani 2	Padova Padova 35128 Italy
0209	Comitato Etico dell'azienda Ospedaliera Policlinico Consorziale di Bari	azienda Ospedaliera Policlinico Consorziale di Bari, Piazza Giulio Cesare 11	Bari Bari 70124 Italy
0210	Comitato Etico Interaziendale della ASO S. Luigi Gonzaga di Orbassano (TO) e delle ASL 5 di Collegno, 6 di Ciriè, 7 di Chivasso, 8 di Chieri, 9 di Ivrea e 10 di Pinerolo	ASO San Luigi Gonzaga, Via Regione Gonzole 10	Orbassano Torino 10043 Italy
0211	Comitato Etico Azienda Spedali Civili di Brescia	Azienda Spedali Civili di Brescia, Piazzale Spedali Civili 1	Brescia Brescia 25123 Italy
0212	Comitato Etico per la Sperimentazione Clinica dei Medicinali dell'Azienda Ospedaliera Universitaria Careggi di Firenze	Azienda Ospedaliera Universitaria Careggi, Viale G. Pieraccini 17	Firenze Firenze 50139 Italy
0213	Comitato di etica per la Ricerca Biomedica dell'Università degli Studi G. D'Annunzio di Chieti e della ASL di Chieti	Università degli Studi G. D'Annunzio, Via dei Vestini 31	Chieti Stazione Chieti 66013 Italy
0214	Comitato Etico dell'ASL di Cagliari	ASL di Cagliari, Via Logudoro 17	Cagliari Cagliari 09127 Italy
0215	Comitato Bioetico dell'Azienda Ospedaliero-Universitaria Policlinico di Catania	Azienda Ospedaliero-Universitaria Policlinico, Via Santa Sofia 78	Catania Catania 95123 Italy
0216	Comitato Etico pe rle attività biomediche dell'Università degli studi Federico II di Napoli	Università degli studi Federico II, Via S. Pansini 5	Napoli Napoli 80131 Italy
0217	Comitato Etico Unico per la	Via Gramsci 14	Parma Parma 43100

Center No.	Ethics Committee or Institutional Review Board	Department / Organization	Address Country
	Provincia di Parma		Italy
0218	Comitato Etico Scientifico dell'Azienda Ospedaliera Niguarda Ca' Granda di Milano	Azienda Ospedaliera Niguarda Ca' Granda, Piazza Ospedale Maggiore	Milano Milano 20162 Italy
0219	Comitato Etico dell'IRCCS Istituto Neurologico Mediterraneo Neuromed di Pozzilli	Neuromed, Via Atinense 18	Pozzilli Isernia 86077 Italy
0220	Comitato Etico dell'Azienda Ospedaliero-Universitaria Policlinico Tor Vergata	Azienda Ospedaliero-Universitaria Policlinico Tor Vergata, Via Oxford 81	Roma Roma 00133 Italy
0221	Comitato Etico della provincia di Ferrara	Corso Giovecca 203	Ferrara Ferrara 44100 Italy
0222	Comitato Etico dell'AUSL di Bologna	Via Castiglione 29	Bologna Bologna 40124 Italy
0251	Egyptian Society of Medical Ethics	Prof. Dr. Haidar Ghaleb	Cairo 59, Ramsis St., Egypt
0252	Egyptian Society of Medical Ethics	Prof. Dr. Haidar Ghaleb	Cairo 59, Ramsis St., Egypt
0253	Egyptian Society of Medical Ethics	Prof. Dr. Haidar Ghaleb	Cairo 59, Ramsis St., Egypt
0254	Egyptian Society of Medical Ethics	Prof. Dr. Haidar Ghaleb	Cairo 59, Ramsis St., Egypt
0255	Egyptian Society of Medical Ethics	Prof. Dr. Haidar Ghaleb	Cairo 59, Ramsis St., Egypt
0301	Universitäts-Klinikum	Ethik-Kommission	Düsseldorf Germany
0303	Landesärztekammer Brandenburg	Ethik-Kommission	Cottbus Germany
0304	Technische Universität München	Ethik-Kommission der Medizinischen Fakultät	München Germany
0305	Ärztekammer Westfalen-Lippe	Ethik-Kommission	Münster Germany
0306	Landesamt für Gesundheit und Soziales Berlin	Geschäftsstelle der Ethik-Kommission des Landes Berlin	Berlin Germany
0307	Universität Duisburg-Essen	Ethik-Kommission der Medizinischen Fakultät	Essen Germany
0308	Universität Greifswald	Ethik-Kommission der Ärztekammer Mecklenburg/Vorpommern	Greifswald Germany
0310	Landesärztekammer Rheinland-Pfalz	Ethik-Kommission	Mainz Germany
0311	Ärztekammer Saarland	Ethik-Kommission	Saarbrücken Germany
0312	Bayerische Landesärztekammer	Ethik-Kommission	München Germany
0313	Landesärztekammer Rheinland-Pfalz	Ethik-Kommission	Mainz Germany
0314	Sächsische Landesärztekammer	Ethik-Kommission	Dresden Germany
0315	Universität Würzburg	Ethik-Kommission	Würzburg Germany
0316	Landesärztekammer Baden-	Ethik-Kommission	Stuttgart

Center No.	Ethics Committee or Institutional Review Board	Department / Organization	Address Country
	Württemberg		Germany
0318	Ärztekammer Nordrhein	Ethik-Kommission	Düsseldorf Germany
0319	Ärztekammer Niedersachsen	Ethik-Kommission	Hannover Germany
0321	Bayerische Landesärztekammer	Ethik-Kommission	München Germany
0322	Zentrum für Ethik und Recht in der Medizin	Ethik-Kommission	Freiburg Germany
0323	Landesärztekammer Hessen	Ethik-Kommission	Frankfurt Germany
0324	Landesamt für Gesundheit und Soziales Berlin	Geschäftsstelle der Ethik-Kommission des Landes Berlin	Berlin Germany
0326	Ethik-Kommission des Landes Bremen		Bremen Germany
0327	Medizinische Hochschule Hannover	Ethik-Kommission	Hannover Germany
0328	Universität Ulm	Ethik-Kommission	Ulm Germany
0329	Universität Tübingen	Ethik-Kommission der Medizinischen Fakultät	Tübingen Germany
0330	Medizinische Fakultät Heidelberg	Ethik-Kommission	Heidelberg Germany
0331	Technische Universität Dresden	Ethik-Kommission der Medizinischen Fakultät	Dresden Germany
0332	Landesärztekammer Brandenburg	Ethik-Kommission	Cottbus Germany
0333	Landesärztekammer Brandenburg	Ethik-Kommission	Cottbus Germany
0361	Ethik-Kommission der Med. Universität Wien		Vienna A-1090 Austria
0362	Ethik-Kommission der Med. Universität Wien		Vienna A-1090 Austria
0363	Ethik-Kommission der Med. Universität Wien		Vienna A-1090 Austria
0364	Ethik-Kommission der Med. Universität Wien		Vienna A-1090 Austria
0365	Ethik-Kommission der Med. Universität Wien		Vienna A-1090 Austria
0366	Ethik-Kommission der Med. Universität Wien		Vienna A-1090 Austria
0381	Ethikkommission beider Basel (EKBB)		Basel Switzerland
0382	Kantonale Ethikkommission des Kantons Zuerich	Spezialisierte Unterkommission Psychiatrie, Neurologie, Neurochirurgie	Zuerich Switzerland
0401	Comité de Etica en Investigaciones Biomédicas		Buenos Aires Buenos Aires C1428AQK Argentina
0402	Comité de Docencia e Investigación INEBA		Buenos Aires Buenos Aires C1192AAW Argentina
0403	Comisión de Bioetica,Comité de Docencia e Investigación		Buenos Aires Buenos Aires C1221ADC

Center No.	Ethics Committee or Institutional Review Board	Department / Organization	Address Country
	del Hospital General de Agudos Ramos Mejía		Argentina
0404	Comité de Etica de Protocolos de Investigación		Buenos Aires Buenos Aires 1181 Argentina
0406	Comité de Etica, Investigación y Docencia: Sanatorio Prof. León S. Morra S.A."		Córdoba Córdoba X5009BIN Argentina
0407	Comité de Etica/ Comité de Docencia de la Fundación Rosarina de Neurorehabilitación		Rosario Santa Fe 2000 Argentina
0408	Comité de Docencia e Investigación		Rosario Santa Fe 2000 Argentina
0421	Comitê de Ética do Hospital Universitário Gaffree e Guinle		Rio de Janeiro RJ 20270-004 Brazil
0422	Comitê de Ética em pesquisa do Hospital dos Servidores do Estado do Rio de Janeiro/HSE		Rio de Janeiro RJ 20221-903 Brazil
0423	Comitê de Ética em Pesquisa - UNICAMP		Campinas SP 13084-971 Brazil
0424	Comitê de Ética em Pesquisa da Irmandade da Santa Casa de Misericordia de Porto Alegre		Porto Alegre RS 90020-090 Brazil
0425	Comitê de Ética do Hospital Sao Rafael		Salvador BA 41253-900 Brazil
0426	Comitê de Ética em Pesquisa HC e FMRP-USP		Riberão Preto SP 14048-900 Brazil
0441	Sydney South West Area Health Service Ethics Review Committee (RPAH Zone)	Research Development Office, Royal Prince Alfred Hospital	Camperdown NSW 2050 Australia
0442	Sydney South West Area Health Service Human Research Ethics Committee (Western Zone)	NSW Health - Sydney South West Area Health Service	Liverpool NSW 1871 Australia
0443	South Eastern Sydney Area Health Service - Human Research Ethics Committee - Northern Network	NSW Health - South East Health	Randwick NSW 2031 Australia
0444	Melbourne Health - Human Research Ethics Committee	Melbourne Health - Research Directorate	Parkville Victoria 3050 Australia
0445	Eastern Health Research & Ethics Committee	Eastern Health	Box Hill Victoria 3128 Australia
0446	Sydney Adventist Hospital Human Research Ethics Committee	-	Wahroonga NSW 2076 Australia
0447	Sydney Adventist Hospital Human Research Ethics Committee	-	Wahroonga NSW 2076 Australia
0502	Quorum Review, Inc		Seattle Washington 98101 US
0504	Quorum Review, Inc		Seattle Washington 98101 US
0505	Quorum Review, Inc		Seattle Washington 98101

Center No.	Ethics Committee or Institutional Review Board	Department / Organization	Address Country
			US
0506	Quorum Review, Inc		Seattle Washington 98101 US
0507	Quorum Review, Inc		Seattle Washington 98101 US
0508	Quorum Review, Inc		Seattle Washington 98101 US
0509	University of Kansas Medical Center	Human Subjects Committee	Kansas City Kansas 66160 US
0510	Quorum Review, Inc		Seattle Washington 98101 US
0512	Quorum Review, Inc		Seattle Washington 98101 US
0513	Western Institutional Review Board		Olympia Washington 98502 US
0514	Quorum Review, Inc		Seattle Washington 98101 US
0516	Western Institutional Review Board		Olympia Washington 98502 US
0517	Quorum Review, Inc		Seattle Washington 98101 US
0518	Quorum Review, Inc		Seattle Washington 98101 US
0520	Copernicus Group IRB		Cary North Carolina 27511 US
0521	Quorum Review, Inc		Seattle Washington 98101 US
0522	Quorum Review, Inc		Seattle Washington 98101 US
0524	Quorum Review, Inc		Seattle Washington 98101 US
0525	Quorum Review, Inc		Seattle Washington 98101 US
0526	The Cleveland Clinic	IRB-WB2	Cleveland Ohio 44195 US
0527	Western Institutional Review Board		Olympia Washington 98502 US
0528	Quorum Review, Inc		Seattle Washington 98101 US
0529	Western Institutional Review Board		Olympia Washington 98502 US
0530	West Virginia University	Office of Research Compliance	Morgantown West Virginia 26506 US
0533	Quorum Review, Inc		Seattle Washington 98101 US
0535	Quorum Review, Inc		Seattle Washington 98101 US
0536	Quorum Review, Inc		Seattle Washington 98101 US
0537	Quorum Review, Inc		Seattle Washington 98101 US
0538	St. Joseph's Hospital and Medical Center	IRB for Human Research	Phoneix Arizona 85013 US
0539	Aurora IRB		Milwaukee Wisconsin 53201 US

Center No.	Ethics Committee or Institutional Review Board	Department / Organization	Address Country
0540	Quorum Review, Inc		Seattle Washington 98101<br>US
0543	Quorum Review, Inc		Seattle Washington 98101<br>US
0544	Yale University School of Medicine	Human Investigation Committee	New Haven CT 06520<br>US
0545	Baylor College of Medicine	Office of Research, IRB for Human Subjects Research	Houston Texas 77030<br>US
0546	Quorum Review, Inc		Seattle Washington 98101<br>US
0601	McGill University Health Ctr	Neurosciences REB	Quebec QC H3A 2B4<br>CA
0602	University of BC	Office of Research Services	Vancouver BC V5Z 1L8<br>CA
0603	Ottawa Hospital	Research Ethics Board	Ottawa ON K1H 8L6<br>CA
0604	Hôpital Charles LeMoyne		Greenfield Park QC J4V 2H1<br>CA
0606	CHUS et de L'Université de Sherbrooke		Sherbrooke QC J1G 2E8<br>CA
0607	St. Michael's Hospital office of Research Administaration		Toronto ON M5C 3G7<br>CA
0608	Fraser Health Research Ethics Board		Surrey BC V3R 7P8<br>CA
0609	Saint-Luc du CHUM		Montreal QC H2W 1Y5<br>CA
0610	IRB Services		Aurora ON L4G 0A5<br>CA
0721	Medical Research Council	Ethics Committee for Clinical Pharmacology	Budapest<br>Hungary
0722	Medical Research Council	Ethics Committee for Clinical Pharmacology	Budapest<br>Hungary
0723	Medical Research Council	Ethics Committee for Clinical Pharmacology	Budapest<br>Hungary
0724	Medical Research Council	Ethics Committee for Clinical Pharmacology	Budapest<br>Hungary
0725	Medical Research Council	Ethics Committee for Clinical Pharmacology	Budapest<br>Hungary
0726	Medical Research Council	Ethics Committee for Clinical Pharmacology	Budapest<br>Hungary
0801	Leeds (West) research Ethics Committee	A/B Floor, Old Site<br>Leeds General Infirmary<br>Great George Street	Leeds LS1 3EX<br>England, UK
0802	Leeds (West) research Ethics Committee	A/B Floor, Old Site<br>Leeds General Infirmary<br>Great George Street	Leeds LS1 3EX<br>England, UK
0803	Leeds (West) research Ethics Committee	A/B Floor, Old Site<br>Leeds General Infirmary<br>Great George Street	Leeds LS1 3EX<br>England, UK
0804	Leeds (West) research Ethics Committee	A/B Floor, Old Site<br>Leeds General Infirmary<br>Great George Street	Leeds LS1 3EX<br>England, UK
0821		IRB	Goyang Kyunggi 411- 769<br>Korea, Republic of
0822		IRB	Seoul Korea 135-710

Center No.	Ethics Committee or Institutional Review Board	Department / Organization	Address Country
			Korea, Republic of
0824		IRB	Seoul Seoul 120-752 Korea, Republic of
0825		IRB	Taegu Taegu 700-721 Korea, Republic of
0901	Local EC of Errikos Denan Hospital / National EC /HA Address: Mesogeion Avenue 284, Holargos, Athens GR-115-62, Greece		ATHENS 115-62 Greece
0902	Local EC of Errikos Denan Hospital / National EC /HA Address: Mesogeion Avenue 284, Holargos, Athens GR-115-62, Greece		ATHENS 115-62 Greece
0903	Local EC of Gennimatas Hospital / National EC /HA Address: Mesogeion Avenue 284, Holargos, Athens GR-115-62, Greece		ATHENS G 15699/ 11562 Greece
0904	Local EC of Ahepa Hospital / National EC /HA Address: Mesogeion Avenue 284, Holargos, Athens GR-115-62, Greece		Thessaloniki GR 54636/ 11562 Greece
0905	Local EC of Ahepa Hospital / National EC /HA Address: Mesogeion Avenue 284, Holargos, Athens GR-115-62, Greece		Thessaloniki GR 54636/ 11562 Greece
0906	Local EC of Hospital of Heraklion/ National EC /HA Address: Mesogeion Avenue 284, Holargos, Athens GR-115-62, Greece		Heraklion Crete GR 711/ 11562 Greece
0915	Comissão de Ética para a Investigação Clínica		Lisboa 1749-004 Portugal
0916	Comissão de Ética para a Investigação Clínica		Lisboa 1749-004 Portugal
0917	Comissão de Ética para a Investigação Clínica		Lisboa 1749-004 Portugal
0918	Comissão de Ética para a Investigação Clínica		Lisboa 1749-004 Portugal
0919	Comissão de Ética para a Investigação Clínica		Lisboa 1749-004 Portugal

The IECs and IRBs listed in this table have been extracted from the clinical study reports of the corresponding clinical trials.
IEC, independent ethics committee; IRB, institutional review board.
