# Supplementary material for: AI-driven reclassification of multiple sclerosis progression
Source: Nat Med. 2025 Aug 20;31(10):3414–24. doi: 10.1038/s41591-025-03901-6 (PMC12532606; doi:10.1038/s41591-025-03901-6)
Supplement: Supplementary file 2 — Reporting Summary [file 41591_2025_3901_MOESM2_ESM.pdf]

nature portfolio

Corresponding author(s): Habib Ganjgahi and Heinz Wiendl

Last updated by author(s): Jun 11, 2025

Reporting Summary

Nature Portfolio wishes to improve the reproducibility of the work that we publish. This form provides structure for consistency and transparency in reporting. For further information on Nature Portfolio policies, see our Editorial Policies and the Editorial Policy Checklist.

Statistics

For all statistical analyses, confirm that the following items are present in the figure legend, table legend, main text, or Methods section.

n/a	Confirmed	
☐	☒	The exact sample size (*n*) for each experimental group/condition, given as a discrete number and unit of measurement
☐	☒	A statement on whether measurements were taken from distinct samples or whether the same sample was measured repeatedly
☐	☒	The statistical test(s) used AND whether they are one- or two-sided<br>*Only common tests should be described solely by name; describe more complex techniques in the Methods section.*
☐	☒	A description of all covariates tested
☐	☒	A description of any assumptions or corrections, such as tests of normality and adjustment for multiple comparisons
☐	☒	A full description of the statistical parameters including central tendency (e.g. means) or other basic estimates (e.g. regression coefficient) AND variation (e.g. standard deviation) or associated estimates of uncertainty (e.g. confidence intervals)
☐	☒	For null hypothesis testing, the test statistic (e.g. *F*, *t*, *r*) with confidence intervals, effect sizes, degrees of freedom and *P* value noted<br>*Give P values as exact values whenever suitable.*
☐	☒	For Bayesian analysis, information on the choice of priors and Markov chain Monte Carlo settings
☒	☐	For hierarchical and complex designs, identification of the appropriate level for tests and full reporting of outcomes
☒	☐	Estimates of effect sizes (e.g. Cohen's *d*, Pearson's *r*), indicating how they were calculated

Our web collection on statistics for biologists contains articles on many of the points above.

Software and code

Policy information about availability of computer code

Data collection	No software was used for data collection
Data analysis	SIENA/X part of FSL 6.0 available at https://fsl.fmrib.ox.ac.uk/fsl/ was used to derive normalised brain volume and percentage brain volume change. The FAHHM code used in this work is available at: https://github.com/habib61/FAHMM

For manuscripts utilizing custom algorithms or software that are central to the research but not yet described in published literature, software must be made available to editors and reviewers. We strongly encourage code deposition in a community repository (e.g. GitHub). See the Nature Portfolio guidelines for submitting code & software for further information.

Data

Policy information about availability of data

All manuscripts must include a data availability statement. This statement should provide the following information, where applicable:

- Accession codes, unique identifiers, or web links for publicly available datasets
- A description of any restrictions on data availability
- For clinical datasets or third party data, please ensure that the statement adheres to our policy

For NO.MS, the reader is able to request the raw data (anonymized) and related documents (e.g., protocol, reporting and analysis plan, clinical study report) of all the studies that underlie the modelling results reported in this article by connecting to CSDPR (https://www.clinicalstudydatarequest.com) and signing a Data Sharing Agreement with Novartis. The data will be made available to researchers, with requests reviewed and approved by an independent review panel of CSDR.

For Roche MS including phase 3 ocrelizumab trial data used for the clinical trial validation, qualified researchers can request access to patient-level data by making a request via Vivli.org
The anonymized MS PATHS dataset used for the real-world validation can be obtained for purpose of replicating this work findings by contacting Prof Wiendl at heinz.wiendl@uniklinik-freiburg.de

Human research participants

Policy information about studies involving human research participants and Sex and Gender in Research.

Reporting on sex and gender	Sex reported for each participant of each individual trial who provided informed consent.
Population characteristics	Demographics and MS feature distributions are compared across datasets in Extended Data Table 2 and Supplementary Fig. 1.2. Demographics and MS disease characteristics from NO.MS are provided for the four clinical (meta-) states in Table 2.
Recruitment	This study uses data from already published clinical trials, no data collection
Ethics oversight	Alta Bates Summit IRB; Asahikawa Medical Center IRB; Ascension Wisconsin IRB; Aurora IRB; Baltimore IRB; Biomedical IRB; CentraState IRB; Central Ethics Committee; Chiba University Hospital IRB; Christiana Care IRB Helen F.; Copernicus Group IRB; Crescent City IRB; Dean IRB; Ebara Hospital IRB; Ehime University Hospital IRB; Georgetown University IRB; Health Sciences Institutional Review Boards; Health Sciences Campus IRB; Health System IRB; Healthcare -IRB; Henry Ford Hospital IRB; Hospital IRB; IRB University of California Davis; IRB of Beijing Hospital; IRB of West China Hospital; IRB-WB2; IRB/OSA; IRBMED; Institutional Ethics Committee, Bakirkoy; Institutional Ethics Committee, Dokuz Eylul; Institutional Ethics Committee, Ege; Institutional Ethics Committee, Gazi; Institutional Ethics Committee, Gaziantep; Institutional Ethics Committee, Hacettepe; Institutional Ethics Committee, Istanbul; Institutional Ethics Committee, Mersin; Institutional Ethics Committee, Uludag; Iwate Medical University Hospital IRB N/A; Johns Hopkins IRB; Keio University Hospital IRB; Kyoto Min-iren Chuo Hospital IRB; Lifespan IRB; Local Ethics Committee of AHEPA; Multicentric Ethics Committee IKEM; NIMS Institutional Ethics Committee; National Ethics Committee; Network IRB; Osaka University Hospital IRB; Pro Health Care IRB Research; Providence Health & Service IRB; Providence Health & Services IRB; Psychiatry IRB; Quorum Review IRB; Research Ethics Committee; Saitama Medical Center IRB; Schulman Associates, IRB; Sone Clinic IRB; The Ethics Committee of Sri; University IRB; University of Colorado Health IRB; University of Utah IRB; WIRB; WakeMed IRB; Wayne State University IRB; Wheaton Franciscan Healthcare IRB N/A

Note that full information on the approval of the study protocol must also be provided in the manuscript.

Field-specific reporting

Please select the one below that is the best fit for your research. If you are not sure, read the appropriate sections before making your selection.

☒ Life sciences ☐ Behavioural & social sciences ☐ Ecological, evolutionary & environmental sciences

For a reference copy of the document with all sections, see nature.com/documents/nr-reporting-summary-flat.pdf

Life sciences study design

All studies must disclose on these points even when the disclosure is negative.

Sample size	This is a retrospective study of MS patients that requires both longitudinal radiological and clinical features of MS patient. In the main analysis and external validation datasets, we used all studies from the NO.MS, Roche MS and MSPATHS subjects that have both radiological and clinical features. This led to use 8023 patients from nine different phase 2 or 3 MS clinical trials from the NO.MS database for the discovery and 2243 patients from Roche phase 3 clinical trials and 2280 patients from the real-world cohort MS PATHS for external validation. Note access to Roche MS and MSPATHS were provided to us after submitting the first version of this paper. The purpose of the analysis is to identify disease states and to estimate transition probabilities and not to estimate a treatment effect - therefore, no sample size calculation is required. NO-MS database is the largest clinical trial database in MS and therefore the best suitable database.
Data exclusions	We did not exclude any patients
Replication	The discovery and replication samples were defined prior to analysis by randomly assigning 80% of patients into validation and remaining into replicating. In addition, we validated the results using pooled data from three independent clinical trials and in a real-world cohort. More details can be found in Methods/Replication and External validation sections.
Randomization	Randomization is used to ensure balanced treatment allocation for all known and unknown difference in the population when estimating a causal treatment effect. Since the purpose of this analysis is to estimate transition probabilities rather than to compare between experimental treatments, no randomization is not needed. This work uses an unsupervised probabilistic machine learning to define a classification of MS using existing clinical trial data. We did not perform intend to treat analysis or evaluate experimental group effect that requires randomisation. However, subjects in each clinical trial data were randomised. Please see table Extended data table 1 for trials used in this study.
Blinding	Blinding is typically used in studies that compare different treatments. The purpose of our analysis is to estimate transition probabilities and to describe treatment evolution over time. No treatment comparison is done and no blinding needed. This is a retrospective analysis of

existing data that uses unsupervised learning to define a new classification for MS.The method clusters MS patient's radiological and clinical features trajectories that have similar pattern without using any labels. Also, the method doesn't use treatment to find the proposed classification and we did not perform intend to treat analysis, hence blinding is not applicable.

Reporting for specific materials, systems and methods

We require information from authors about some types of materials, experimental systems and methods used in many studies. Here, indicate whether each material, system or method listed is relevant to your study. If you are not sure if a list item applies to your research, read the appropriate section before selecting a response.

Materials & experimental systems

n/a	Involved in the study
☒	☐ Antibodies
☒	☐ Eukaryotic cell lines
☒	☐ Palaeontology and archaeology
☒	☐ Animals and other organisms
☐	☒ Clinical data
☒	☐ Dual use research of concern

Methods

n/a	Involved in the study
☒	☐ ChIP-seq
☒	☐ Flow cytometry
☒	☐ MRI-based neuroimaging

Clinical data

Policy information about clinical studies

All manuscripts should comply with the ICMJE guidelines for publication of clinical research and a completed CONSORT checklist must be included with all submissions.

Clinical trial registration	This work uses data from already conducted clinical trials on MS. All individual trial reports have previously been published. The list of studies and registration numbers can be found in Extended Data Table 1
Study protocol	Not applicable, each clinical trial protocol was already published
Data collection	Retrospective analysis of already conducted and published clinical trials
Outcomes	This is a multivariate modeling of all available standard clinical and radiological outcomes in MS clinical trials as described in Extended Data Table 2
